# Identifying areas and centers of endemism in the Gran Chaco with Fabaceae as a diversity indicator

**DOI:** 10.1038/s41598-025-90091-3

**Published:** 2025-03-20

**Authors:** Matías Morales, Angela Lúcia Bagnatori Sartori, Darién Eros Prado, Renée H. Fortunato

**Affiliations:** 1 Instituto de Recursos Biológicos, CIRN–CNIA, Instituto Nacional de Tecnología Agropecuaria (INTA), N. Repetto & Los Reseros s.n., 1686 Hurlingham, Buenos Aires Argentina; 2 Consejo Nacional de Investigaciones Científicas y Técnicas (CONICET), Godoy Cruz 2290, 1425 Buenos Aires, Ciudad Autónoma de Buenos Aires Argentina; 3 Escuela Superior de Ingeniería, Informática y Ciencias Agroalimentarias, Universidad de Morón, Machado 854, 1708 Morón, Buenos Aires Argentina; 4 Universidade Federal de Mato Grosso do Sul, Av. Costa e Silva s/n°, Bairro Universitário, Campo Grande, MS Brazil; 5 IICAR & Facultad de Ciencias Agrarias, Universidad Nacional de Rosario, Campo Experimental Villarino, C.C. 14 (S2125ZAA) Zavalla, Santa Fe Argentina; 6 Instituto de Botánica Darwinion (CONICET/ANCEFYN), Labardén 200, B1642HYD San Isidro, Buenos Aires Argentina

**Keywords:** Ecology, Plant sciences

## Abstract

The Gran Chaco ecoregion is the second largest forest in South America and exhibits the highest deforestation rate in the world, threatened by the expansion of agriculture, lodging and cattle grazing. The Chacoan endemism richness has still been not adequately investigated. In this work, we detected areas of endemism (AEs) and centers of endemism (CEs) using Fabaceae as a proxy, with a novel combination of analyses to explore the endemism distribution pattern of Chacoan plants: NDM-VNDM analyses to detect AEs, and endemic indexes and clustering to detect CEs. We recovered regions with high concentration of endemic and highly restricted taxa: (1) Sierra Chaco; (2) Sierra/Arid Chaco ecotone; (3) the interior Dry Chaco; (4) the upper Paraguay River Basin; and (5) the Low Paraguay–Paraná River Basins. We also determined which taxa contributed to configure the AEs and CEs, the environmental variables explaining their potential distribution, the ecological niche overlap and preferences of habitat between them. Dry Interior Chaco and the Dry/Sierra Chaco ecotone included taxa sharing similar and highly overlapped niches. The remainder, located in Humid and Sierra Chaco, showed restricted and variably overlapped ecological niches, suggesting a complexity of habitats requiring further intensive sampling for conservation measures. Unweighted Pair Group Method with Arithmetic Mean (UPGMA) also recovered grouping patterns contributing to improve the regionalization of the Gran Chaco based on the exclusive species of its biota. These areas are potential biodiversity hotspots and speciation centers for the native flora in subtropical South America and can serve as tools for conservation policies, especially in Sierra Chaco and Dry Chaco.

**Supplementary Information:**

The online version contains supplementary material available at 10.1038/s41598-025-90091-3.

## Introduction

The Gran Chaco ecoregion is the second-largest forest in South America behind Amazon rainforest, the largest dry forest in the region. It covers 1,066,000 km^2^ throughout subtropical South America (eastern Bolivia, southwestern Brazil, western Paraguay, and central-northern Argentina)^1,2^. This region is one of the few vast areas with forests and savannas forming a transition between tropical and temperate areas in the world, and it is one of the most threatened subtropical regions by deforestation^3^. This is presumably one of the most diverse extratropical ecoregions of South America, but its floristic diversity was still not adequately analyzed, especially regarding to the number and proportion of endemics species and its threats. It is still lacking an exhaustive inventory of the native flora, although several initiatives are ongoing by means of regional floras in Argentina (for example, the preliminary notes and chapters of the “Chacoan flora”^4^, and the last volume of^5^), Paraguay^6^, and Brazil^7^.

Although in appearance, the Gran Chaco seems to be a region with relatively few endemic taxa, when compared with adjacent ecoregions such as Atlantic Forests^8^, more recent works have suggested that its diversity is underestimated. For instance, in Paraguay, according to a recent census^9^ at least 20% of the total endemic taxa of the country are restricted to Gran Chaco and its surrounding areas. Several Chacoan sectors still remain to be undersampled^10^ or, at least, they have not been investigated. In spite of these multiple gaps in flora documentation in the Gran Chaco, other South American ecoregions, such as the Atlantic Forests and Pampas, have been historically well explored by botanists and they have now extensive inventories of species^11^.

The Gran Chaco ecoregion can be divided into different districts or subregions, according to different authors: Humid Chaco, Dry Chaco, and Sierra Chaco^10,12^) which differ in climatic regime, vegetation and soils. Humid Chaco comprises the eastern region of the Gran Chaco, exhibiting comparatively higher amount annual of precipitations and more regularly distributed in the year than Dry Chaco, located in the western area of the ecoregion. However, Sierra Chaco, located at southwestern extreme and small sectors of the western area of the ecoregion, exhibits a gradient of precipitations and temperatures according to altitude differences^1^. These differences determine the presence of different types of vegetation and different floristic composition of the formations, as well as different levels of endemism. Consequently, Dry Chaco and Sierra Chaco are the richest subregions with endemic or highly restricted species^10^. A fourth subregion, the Arid Chaco has been also recognized by several authors, becoming the driest area of the Dry Chaco^13^.

The Fabaceae (Leguminosae) is one of the most diversified families in the Gran Chaco. There are 300 species and 20% of their infraspecific and specific taxa are endemic. Furthermore, ca. 60% of the species are related to some endemic lineages^10^. This means that this plant family is a good indicator or “proxy” to understand the ecological basis of the current distribution of the flora in this ecoregion. It is possible because of the ecological dominance of the family in the Gran Chaco ecoregion and the omnipresence and high diversity of its genera in this area^10^. All these facts imply that this family is potentially useful to identify Chacoan hotspots of biodiversity.

Objectives such as improving regionalization and the detection of biodiversity hotspots can be accomplished by means of methodologies based on the distribution of the biota. These approaches include, for example, Areas of Endemism (AE), Centers of Endemism (CE) and hierarchical clustering methods, such as Parsimony Analysis of Endemism (PAE) or Unweighted Pair Group Method with Arithmetic Mean (UPGMA)^14^. Although all of these methods are generally used in biogeography, hierarchical methods are more focused in classification of areas, but they frequently do not reflect the presence of ecotones^15^.

The former method, known as Areas of Endemism (AEs) considers areas inhabited by taxa with congruent patterns of distribution^16^. They are assumed to generate similar responses to different environmental factors, converging to similar ranges of distribution. These areas are expected to exhibit overlapping, since different factors are affecting in different ways different components of the biota. Consequently, it is common to find partial overlapped and nested patterns of AEs^15^. Another approach to study regionalization and species richness is the delimitation of Centers of Endemism (CEs), which are geographic areas where endemic species are common to a whole center of an area or region^17^. The definition of centers of endemism could be useful for regionalization purposes, as well as for planning conservation^18^. It could help identify priority areas to preserve, especially when funds and available territory are restricted^19^.

The objectives of this work were: (1) to identify areas and centers of endemism in the Gran Chaco ecoregion, focusing in the Fabaceae family; (2) to identify the taxa that define these areas and centers; (3) to determine the environmental variables that explain their existence of these areas; (4) to identify implications and applications of this information for further research in conservation and biogeography at regional scale. The main goal was to detect potential biodiversity hotspots considering that Fabaceae is an adequate proxy for the South American biogeographic regionalization and for conservation purposes.

## Materials and methods

### Plant database

Firstly, we built a database with specific and infraspecific endemic and restricted taxa for Fabaceae from the Gran Chaco ecoregion. The criteria for including taxa were based on our previous classification from the Chacoan flora^10^. The database included the specific and infraspecific taxa with the range of “Chacoan-endemic” and having “Chacoan lineage” or “Chacoan/Seasonally Dry Tropical Forests (SDTF) lineage”. We assigned the status of “endemic” to those taxa which were registered exclusively within the boundaries of the Gran Chaco.

We also analyzed the lineage, based on criteria of^20^ with modifications of^10^: the taxa attributed to “Chacoan-lineage” were those registered in the Gran Chaco as well as in the surrounding ecoregions grouped in the Chaco Domain, such as Monte, Pampa, and Espinal. The taxa exhibiting Chacoan/SDTF lineage were recorded either in ecotones between Chacoan and SDTF formations or in mixed Chaco/SDTF formations. The SDTF concept has been explained and mapped in detail by us in previous works^10,21,22^). For our studies about modeling and ecological niches, the dataset was improved by means of removing localities of the same taxa if they were distant up to 5 km in the R Environment, except those taxa with less than 10 occurrences.

We registered the localities of occurrence, based on the information that we previously compiled^10^ but improved by adding new occurrences. For that objective, we visited herbaria with representative specimens of the Chacoan flora (BAB, CGMS, CPAP, CTES, MO, NY, SI), compiled data from the databases of specimens from the taxa occurrences by administrative divisions in Flora del Cono Sur^23^. In addition, we also included information from field observations and recently collected specimens in several field trips across the region in recent years (Argentinean Chaco: 2021, 2022; Brazilian Chaco: 2004–2020). We complemented the information with data from online herbarium databases, such as TROPICOS (https://tropicos.org/3E) or Species Link (https://splink.cria.org.br/3E). However, we adopted these sources only when the specimen’s images were available or determinations were reliable. All the plant material that we used complies with guidelines of our institutions and with the national and international laws, as well is explained in the respective section.

### Study area

The background area included the Gran Chaco ecoregion, with the boundaries previously proposed by us^10^ and surrounding locations, such as the Espinal, Pampas, Monte, Mesopotamic Savannas, Alto Paraná Atlantic Forests, Araucaria Humid Forests, Inter-Andean Dry Valleys and Seasonally Dry Tropical Forest ecoregions^10,12^. Therefore, this area included the entire range of many species that are highly restricted to the Gran Chaco but occurring in the adjacent ecoregions. Our background was based in a stack delimitated by 70°W and 50°W and 16°S and 41°S. The study area was divided in 500 grid cells of 1° × 1°, which was the basis of main analyses of endemism, although for previous screening of the Analysis of Endemism we also mapped grid cells of 1.25° × 1.25° and 0.75° × 0.75°.

In addition, to taking into account the possible subsampling of certain areas of the Gran Chaco ecoregion, we mapped all other records of native flora from the GBIF database, assuming a subsample of 10,000 accessions for the most diverse plant families in the region (Asteraceae, Fabaceae and Poaceae). Later, we calculated the number of records per grid cell, and ranged them according to the deciles 0, 0.1, 0.9 and 1; the grid cells were classified in three categories: (1) not sampled; (2) scarcely sampled; (3) adequately sampled. We used the *raster* package^24^ for reading, writing and manipulating spatial data.

### Determination of areas of endemism (AE)

We estimated the Areas of Endemism (AEs) by means of the Endemicity Analysis using the method of Szumik and Goloboff^16^) in the NDM/VNDM software^25^. We evaluated different grid cell sizes (0.75° × 0.75°, 1° × 1°, 1.25° × 1.25°, and 1.5° × 1.5°). The grid cell sizes were based on previous works (e.g^26–29^). , for the Southern Cone South American region.

We ran analyses with different radius size filling (10, 25, and 50% of cell size). We assumed that these filling rates are adequate, considering, on the one hand, that some areas of Gran Chaco seemed to be scarcely sampled and, on the other hand, that there is a strong environmental gradient in western areas of the region. The conditions of the analyses in NDM/VNDM were the following: swapping a cell at a time, 100 repetitions, saving sets with score higher than 2, discarding superfluous sets as they are found, and replacing a set improved during swapping. We saved the sets with two or more endemic species. Later, we estimated the consensus areas, considering a relaxed consensus rule (30%). We also registered the taxa that contributed to the scores of the AEs. AEs are indicated in the NDM/VNDM software.

### Determination of centers of endemism (CE)

The centers of endemism represent a different way to delimitate areas of high conservation and diversity values to the AEs. They are mainly areas where endemic species are frequently occurring^14^ and they are not focused strictly in the biogeographical history. We defined the centers of endemism in the Gran Chaco region by means of two approaches: the calculation of endemicity indexes and a cluster analysis based on similarity matrices. These approaches have been used in recent works of endemic floras^14,30^). Firstly, we built a matrix of presence/absence of taxa in the area of study, considering a grid cell size of 1° × 1°. We used QGIS 3.14^31^ to join the attributes of the matrices and the maps of the region.

In the first case (endemicity indexes), we calculated the Endemic Richness (ER), the Relative Endemic Richness (RER) and the Weighted Endemic Richness (WER) indexes^18^. ER was calculated as the sum of endemic taxa per grid cell, while considered the “range-restricted” taxa to calculate RER as those occurring in maximally 3 grid cells; WER was calculated in two steps: (1) calculation the inverse of the cells occupied by each species (1/number of grid cells where species occurs in); (2) sum of weighted scores per grid cell.

In the second case (cluster analysis) we built a matrix of similarity, based on the similarity coefficient of Jaccard, which has been largely used in ecology community studies^32,33^) and performed a cluster analysis. We used the functions *dist* and *hcluster* in the R Environment^34^ to build the dissimilarity matrix and to run the cluster analyses, respectively. We selected the UPGMA clustering algorithm instead of PAE, since its performance hass been better to identify CEs of flora in recent works^14^.

For the delimitation and identification of the centers of endemism, we considered them as “cores” of ampler biogeographic regions, based in the occurrence of endemic taxa. Consequently, we performed two cluster analyses: (1) including all endemic taxa, to infer the presence of some regionalization pattern; (2) including a limited number of endemic taxa, assuming the number of taxa that maximizes the number of endemic taxa, but preserving as close as possible to the clustering pattern found in 1).

The representative taxa of the CEs were determined assuming the following criteria: (1) in CEs determined by means UPGMA, we considered those taxa occupying more than 50% of the total cells in each CE; (2) in CEs determined by means the Endemic Richness indexes, we considered the entire set of taxa occurring in these cells.

### Occurrences and distribution models

We developed distributional models in order to mitigate range under-commission in poorly sampled areas and to explore the bioclimatic variables that are influencing the occurrence of the endemic taxa. The primary criterion for selecting taxa was that they contributed to the definition of the endemism areas detected by NDM-VNDM. However, in many cases, the taxa performing these areas had insufficient occurrences to generate reliable models. In this case, we alternatively selected species contributing to the Centers of Endemism detected by other methods with an adequate number of localities giving reliable models.

For selected taxa contributing to AEs partially or not overlapping, we developed models of current distribution by means of the MAXENT algorithm^35^, to explore the areas with suitable environmental conditions for growing endemic Chacoan taxa. In order to prevent methodological restrictions and biases, we only analyzed the endemic and restricted taxa of legumes having more than 5 occurrences.

We initially evaluated the entire set of bioclimatic variables from WORDCLIM 2.1 version^36^ at a resolution of 30 s. For this aim, we made a correlation analysis by means of the calculation of the Pearson correlation coefficient and their significance levels in the *ecospat* package of the R environment^37^. We considered a threshold of 0.75 and significant differences between them.

Finally, we used a set of 6 uncorrelated bioclimatic variables of that were previously considered to influence in the current distribution of the studied taxa. We compared them with the models of the entire set of bioclimatic variables and a mix of bioclimatic and edaphic variables from the SoilGrids project (<https://SoilGrids250m 2.0>), but they were similar to the models of the set of 6 uncorrelated variables. This set of 6 variables was chosen as the set of explanatory variables to run the final models. We ran MAXENT in the ENMEval package^38^, which allows selecting the best model varying the regularization multipliers and the feature classes.

In that package, we evaluated three regularization multipliers: “linear”, “linear + quadratic” and “linear + quadratic + hinge” and three regularization multipliers (1 to 3), excepting in those taxa with less than 10 occurrences; in this case, we used only two features for the evaluation (“linear” and “linear + quadratic”). We partitioned the occurrences using the “Spatial Block” method. The background consisted in 10,000 occurrences that were randomly sampled from the raster stack. We selected the partition algorithm “maxent.jar”. Once the models were run, we selected the best of them considering the lowest Akaike’s information criterion corrected for small samples (AICc). We mapped the logarithmic output and explored the contribution of each variable to the selected models, considering the values after permutation. After the generation of the distribution models, we performed a stack in DIVA–GIS software by summing the models in each endemism area.

### Overlap analysis of ecological niches

Once we obtained the AEs, CEs and the taxa contributing to them, we analyzed the overlap degree and the similarity of the ecological niches of the taxa. It allowed us to define and compare the environmental space that each one of the endemic taxa is occupying in the Gran Chaco, and to find common patterns to help answering questions regarding the environmental conditions delimitating areas with endemisms.

We used the Principal Component Analysis (PCA) method, according to^39^ by means of the ‘*ecospat*’ package in the R Environment^40^to compare the ecological niches of paired taxa contributing to the areas of endemism. We firstly created a grid with occurrence densities along the environmental gradients in each representative taxa of AEs, based on the occurrence data of each taxon, its background (performing a circular buffer area of 400 km around each occurrence) and the background of the stack including the 6 uncorrelated environmental variables in the studied area (this background was extracted for the two first axes of the PCA-environment of the 6-Bioclimatic variables stack).

The two-dimensional environmental space (using all the 6 bioclimatic variables) was then projected onto a 100 × 100 PCA grid of cells restricted by the minimum and maximum PCA values in the background. Then, we calculated the Schoener’s *D* index of niche overlap and run the similarity test between pairs of taxa^41^, from the density occurrences of each pair of taxa contributing to the respective AE. For this purpose, we applied 1000 replications to perform the results. The circle of correlation of variables was also analyzed. The categories of the niche overlap were based on^42^.

In order to quantify the comparison between ecological niches, we calculated the niche expansion, stability and unfilling using the ecospat.niche.dyn.index function in *ecospat*^43^. Although this approach is more frequently used in the case of biological invasions, it is feasible to adopt it for comparing native niches of different species^44^. Niche stability is the proportion of the niche of one species overlapping with a second one; niche unfilling is the proportion of the native species non overlapping with the invader or former species; and niche expansion is the proportion of the invader species non-overlapping with the native.

## Results

### Plant database and study area

**Fig. 1:**
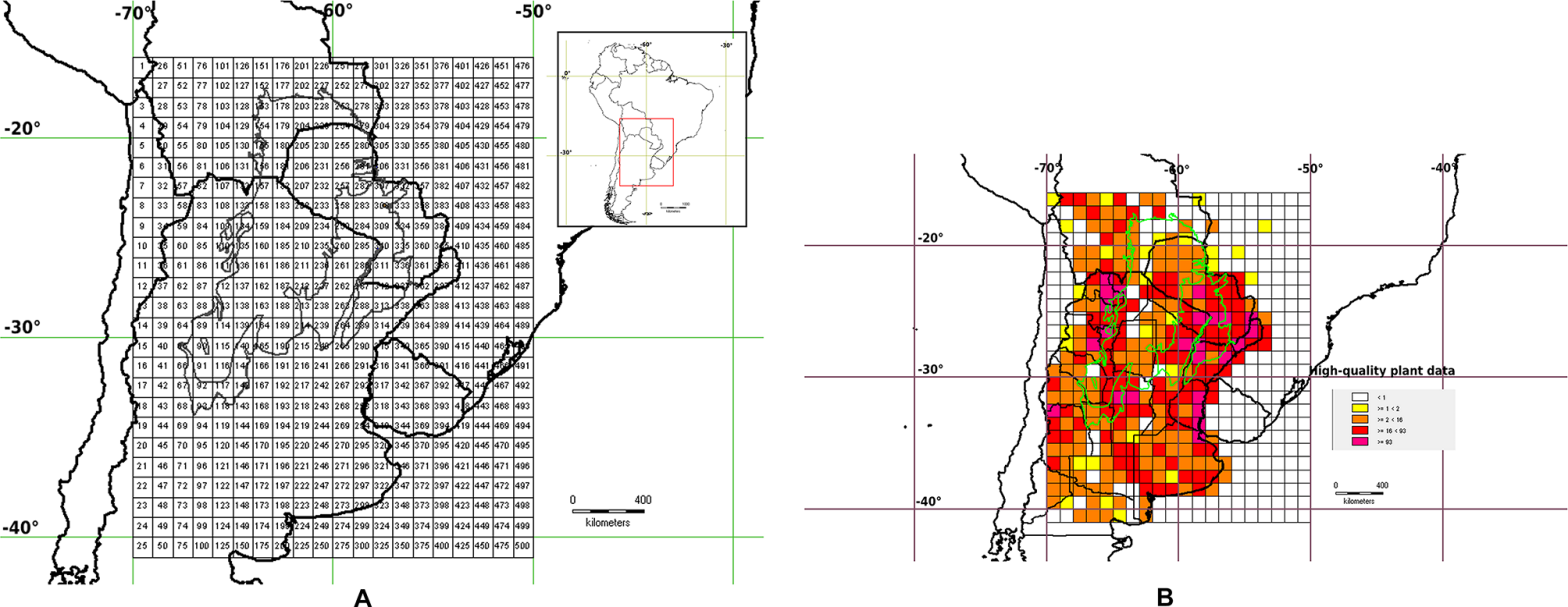
(**A**) Study area and grids. (**B**) Grids indicating quality data of sampling, according to the calculated quartiles.

We assembled data to reach 1,957 localities of the endemic and restricted taxa of Fabaceae whose taxonomic identity and coordinates were adequately checked. Our assessment recovered a checklist with 78 taxa which were endemic, highly restricted and/or exhibited Chacoan lineage but present in the Gran Chaco ecoregion (Table 1). The analyzed specimens were listed in the Suppl. Files (Suppl. Files: Appendix). The area of study comprised the Gran Chaco ecoregion and surrounding areas, which was divided for main analyses in 1° × 1° grid cells (Fig. 1a). According to our registers, Dry Chaco resulted the most undersampled subregion (Fig. 1b).

**Table 1:** Specific and infraspecific taxa of Fabaceae endemic or highly restricted in the Gran Chaco ecoregion and their lineage and endemic status. SDTF: seasonally dry Tropical forests. Endemic are mainly of Chacoan lineage, excepting if it is indicating another.

Taxon	Subfamily	Status, lineage	Occurrences
*Adesmia cordobensis*	Papilionoideae	Endemic	47
*Adesmia macrostachya*	Papilionoideae	No endemic, Chacoan lineage	7
*Aeschynomene magna*	Papilionoideae	Endemic	2
*Aeschynomene paraguayensis*	Papilionoideae	Endemic	2
*Apurimacia dolichocarpa*	Papilionoideae	Endemic	11
*Arachis batizocoi*	Papilionoideae	Endemic	9
*Arachis correntina*	Papilionoideae	Endemic	26
*Arachis duranensis*	Papilionoideae	Endemic	23
*Arachis lignosa*	Papilionoideae	Endemic	5
*Arachis microsperma*	Papilionoideae	Endemic	2
*Arquita mimosifolia*	Caesalpinioideae	No endemic, Chacoan lineage	15
*Astragalus distinens*	Papilionoideae	No endemic, Chacoan lineage	13
*Bauhinia argentinensis*	Cercidoideae	No endemic, Chacoan lineage	16
*Bauhinia hagenbeckii*	Cercidoideae	No endemic, Chacoan/SDTF lineage	15
*Chaetocalyx chacoensis*	Papilionoideae	Endemic	6
*Chamaecrista arachiphylla*	Caesalpinioideae	Endemic	6
*Chloroleucon chacoense*	Caesalpinioideae	Endemic	13
*Clitoria cordobensis*	Papilionoideae	Endemic	3
*Crotalaria chaco-serranensis*	Papilionoideae	Endemic	40
*Dalea elegans*	Papilionoideae	No endemic, Chacoan lineage	72
*Denysophytum stuckertii*	Caesalpinioideae	Endemic	33
*Desmanthus tatuhyensis var. brevipes*	Caesalpinioideae	Endemic	17
*Desmodium burkartii*	Papilionoideae	Endemic	2
*Desmodium intermedium*	Papilionoideae	Endemic	2
*Dolichopsis paraguariensis*	Papilionoideae	Endemic	49
*Erythrostemon argentinus*	Caesalpinioideae	Endemic	9
*Eryrthrostemon coluteifolius*	Caesalpinioideae	Endemic	16
*Galactia glaucophylla*	Papilionoideae	Endemic	36
*Galactia longifolia*	Papilionoideae	No endemic, Chacoan lineage	9
*Indigofera guaranitica*	Papilionoideae	Endemic	4
*Indigofera kurtzii*	Papilionoideae	Endemic	6
*Indigofera parodii*	Papilionoideae	Endemic	90
*Lathyrus nigrivalvis*	Papilionoideae	Chaco	16
*Libidibia paraguariensis*	Caesalpinioideae	Endemic	51
*Lophocarpinia aculeatifolia*	Caesalpinioideae	Endemic	42
*Mimosa castanoclada*	Caesalpinioideae	Endemic	19
*Mimosa centurionis*	Caesalpinioideae	Endemic	3
*Mimosa chacoensis*	Caesalpinioideae	Endemic	13
*Mimosa cordobensis*	Caesalpinioideae	Endemic	8
*Mimosa craspedisetosa*	Caesalpinioideae	Endemic	3
*Mimosa detinens*	Caesalpinioideae	Endemic	70
*Mimosa morongii*	Caesalpinioideae	Endemic	4
*Mimosa pseudopetiolaris*	Caesalpinioideae	Endemic (SDTF)	6
*Mimosa sensibilis*	Caesalpinioideae	Endemic (SDTF)	115
*Mimosa tobatiensis*	Caesalpinioideae	Endemic (SDTF)	4
*Mimosa troncosoae*	Caesalpinioideae	Endemic (SDTF)	1
*Mimozyganthus carinatus*	Caesalpinioideae	Endemic	104
*Neltuma affinis*	Caesalpinioideae	No endemic, Chacoan lineage	65
*Neltuma alba var. alba*	Caesalpinioideae	No endemic, Chacoan lineage	64
*Neltuma campestris*	Caesalpinioideae	Endemic	8
*Neltuma elata*	Caesalpinioideae	Endemic	20
*Neltuma fiebrigii*	Caesalpinioideae	Endemic	17
*Neltuma flexuosa*	Caesalpinioideae	No endemic, Chacoan lineage	32
*Neltuma hassleri*	Caesalpinioideae	Endemic	28
*Neltuma kuntzei*	Caesalpinioideae	Endemic	146
*Neltuma nigra var. longispinna*	Caesalpinioideae	Endemic	2
*Neltuma nigra var. nigra*	Caesalpinioideae	No endemic, Chacoan lineage	69
*Neltuma nigra var. ragonesei*	Caesalpinioideae	Endemic	2
*Neltuma nuda*	Caesalpinioideae	Endemic	12
*Neltuma pugionata*	Caesalpinioideae	Endemic	21
*Neltuma rojasiana*	Caesalpinioideae	Endemic	9
*Neltuma rubriflora*	Caesalpinioideae	Endemic (SDTF)	16
*Neltuma sericantha*	Caesalpinioideae	No endemic, Chacoan lineage	67
*Piptadeniopsis lomentifera*	Caesalpinioideae	Endemic (SDTF)	20
*Senegalia emilioana*	Caesalpinioideae	Endemic	22
*Senegalia praecox*	Caesalpinioideae	No endemic, Chacoan lineage	39
*Senna chacoensis*	Caesalpinioideae	Endemic	13
*Senna chloroclada*	Caesalpinioideae	Endemic	57
*Senna subulata*	Caesalpinioideae	No endemic, Chacoan lineage	21
*Stenodrepanum bergii*	Caesalpinioideae	No endemic, Chacoan lineage	7
*Strombocarpa abbreviata*	Caesalpinioideae	No endemic, Chacoan lineage	7
*Stylosanthes recta*	Papilionoideae	Endemic	6
*Tephrosia hassleri*	Papilionoideae	Endemic	9
*Tephrosia chaquenha*	Papilionoideae	Endemic	6
*Vachellia astringens*	Caesalpinioideae	Endemic	38
*Vachellia caven var. microcarpa*	Caesalpinioideae	Endemic	13
*Vachellia curvifructa*	Caesalpinioideae	Endemic	37
*Vicia epetiolaris*	Papilionoideae	No endemic, Chacoan lineage	28
*Vicia graminea*	Papilionoideae	No endemic, Chacoan lineage	7

### Areas of endemism

The NDM/VNDM analysis of endemism assuming a grid cell size of 0.75° × 0.75° showed scarce AEs and consensus AEs, considering different filling size. The analysis of the AEs assuming the maximum size (1.5° × 1.5°) recovered the maximum number of AEs and consensus AEs (Table 2), but the circumscription of cells was extremely large, with cells including excessive environmental heterogeneity, and it resulted inconvenient for an adequate biogeographic interpretation.

**Table 2:** Consensus areas of Endemism of Fabaceae in the Gran Chaco ecoregion.

Grid size	Filling size		
	10	25	50
0.75° × 0.75°	1	3	3
1° × 1°	4	4	6
1.25° × 1.25°	8(6)	7(6)	8(6)
1.5° × 1,0.5°	11	7	10

() = Not or partially overlapped areas.

**Fig. 2:**
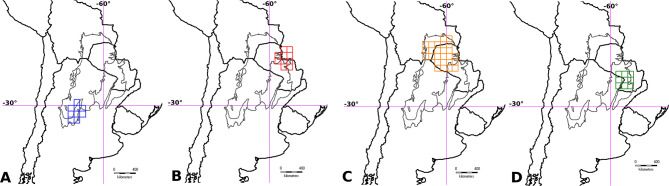
Selected Areas of Endemism in the Gran Chaco Ecoregion based on a grid cell size of 1° × 1°. (**A**) Sierra Chaco; (**B**) Upper Paraguay River Basin; (**C**) Interior Dry Chaco; (**D**) Low Paraguay and Paraná River Basins. Grey thin lines delimitate the three subregions of the Gran Chaco ecoregion.

The analysis of endemism analysis assuming a grid cell size of 1° × 1° and filling 25% of cells recovered 20 areas of endemism throughout the Gran Chaco ecoregion. These areas were grouped in five consensus areas, two in Dry Chaco, two in Humid Chaco, and one in Sierra Chaco (Table 2; Fig. 2). The grid cell size considering a filling of 10 and 50% showed a little higher number of consensus AEs than 25%, but some of them were mainly overlapped and included the same taxa. The taxa contributing to perform each AEs in this grid size belonged mainly to Caesalpinioideae and Papilionoideae families, excepting for *Bauhinia hagenbeckii* Harms in the Upper Paraguay River Basin, which belongs to Cercidoideae (Table 3; Suppl. Files: Table 1).

**Table 3:** Areas of Endemism in the Gran Chaco based on the Fabaceae diversity (grid cell size: 1° × 1°, cell fill 0.25 × 0.25).

Area of endemism	Representative endemic and high restricted taxa
Low Paraguay–Paraná River Basins	*Aeschynomene paraguayensis*,* Desmodium intermedium*,* Mimosa morongii*,* M. pseudopetiolaris*,* Tephrosia hassleri.*
Dry Chaco	*Senegalia emilioana*, *Lophocarpinia aculeatifolia*,* Mimosa castanoclada*,* Piptadeniopsis lomentifera*,* Neltuma nuda*,* Stylosanthes recta*.
Dry Chaco	* Arachis batizocoi*,* Chaetocalyx chacoensis*,* Lophocarpinia aculeatifolia*,* Mimosa castanoclada*,* Piptadeniopsis lomentifera*,* Neltuma nuda*,* Stylosanthes recta, Senegalia emilioana*.
Upper Paraguay River Basin	*Aeschynomene magna*,* Arachis lignosa*,* A. microsperma*,* Neltuma rubriflora*
Sierra Chaco	*Apurimacia dolichocarpa*,* Clitoria cordobensis*,* Indigofera kurtzii*,* Mimosa cordobensis.*

The analysis of endemism assuming a grid cell size of 1.25° × 1.25° and filling 25% of cells recovered 35 areas of endemism throughout the Gran Chaco ecoregion. These areas were grouped in 7 consensus areas (Table 2), three in interior Dry Chaco (but two including ecotones with the Humid Chaco), three in Humid Chaco, one in Sierra Chaco and one in the ecotone of Dry and Sierra Chaco. For this reason, the only different AE identified with this configuration of cells was the ecotone beween Dry and Sierra Chaco (Suppl. Files: Fig. 1).

### Centers of endemism

The centers of endemism (CEs) ranged from 5 to 10, and 7 to 30, respectively. The UPGMA and the ER showed the highest number of cells and CEs, while the WER showed the lowest values in both parameters (Table 4).

**Table 4:** Number of centers of Endemism of Fabaceae in the Gran Chaco ecoregion.

Method	Cells	Centers of Endemism
Unweighted Pair Group Method with Arithmetic Mean (UPGMA)	30	5
Endemic Richness (ER)	17	10
Restricted Endemic Richness (RER)	7	5
Weighted Endemic Richness (WER)	14	7

**Fig. 3:**
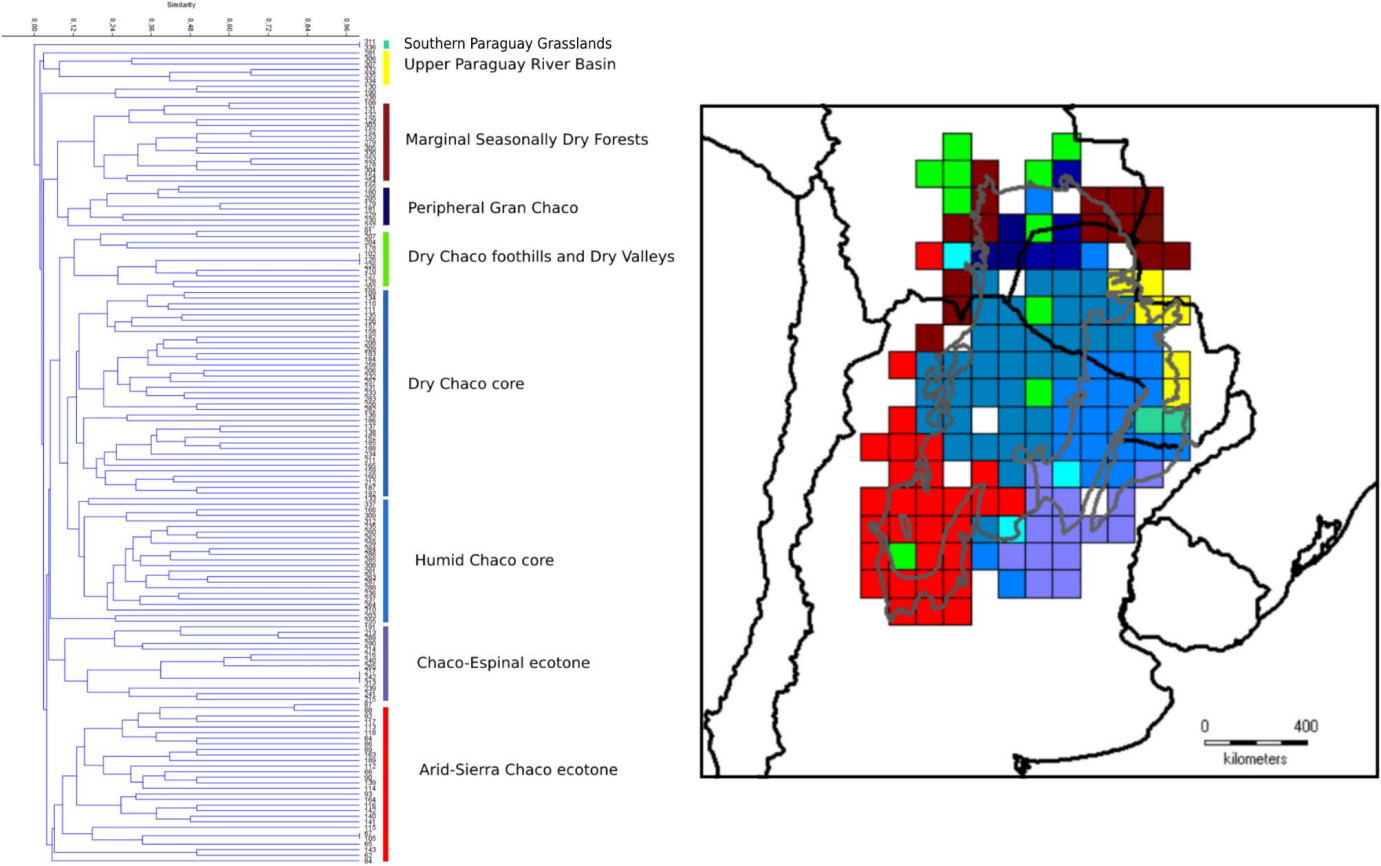
Cluster Analysis (CA) and mapping of cells by means UPGMA of the Gran Chaco Ecoregion based on endemic and highly restricted Fabaceae, calculated with Jaccard similarity index. Cells lacking presences were discarded. Grey thin lines delimitate the three subregions of the Gran Chaco ecoregion. Numbers in terminals of the CA indicate the number of the grid cell.

The UPGMA hierarchical clustering including all cells with at least one taxa showed a clustering with the following groups, which should be potential biogeographic areas: (1) Southern Paraguay Grasslands; (2) Upper Paraguay River Basin; (2) Marginal Dry Seasonally Forest; (3) Peripheral Gran Chaco; (4) Dry Chaco foothills and Dry Valleys; (5) Dry Chaco core”; (6) Humid Chaco “core”; (7) Chaco–Espinal ecotone; (8) Dry/Sierra Chaco ecotone (Fig. 3; Table 5).

**Table 5:** Characterization of the centers of endemisms in the Gran Chaco based on the Fabaceae diversity, detected by means UPGMA hierarchical analyses using Jaccard similarity index.

Center of endemism	Definition	Potential biogeographic area (according to Cluster Analysis)	Representative endemic and high-restricted taxa	Area(s) of endemism
Upper Paraguay River Basin	Brazilian Chaco and adjacent Paraguay	Upper Paraguay River Basin	*Aeschynomene magna*, *Arachis lignosa*,* Neltuma rubriflora*,* Tephrosia chaquenha*	Upper Paraguay River Basin
Dry/Sierra Chaco ecotone	Central Argentina	Dry/Sierra Chaco ecotone	*Adesmia cordobensis*,* Adesmia macrostachya*,* Apurimacia dolichopcarpa*,* Arquita mimosifolia*,* Astragalus bergii*,* Galactia glaucophylla*,* Indigofera kurtzii*,* Indigofera parodii*,* Mimosa cordobensis Strombocarpa abbreviata*,* Neltuma campestris*,* Neltuma. sericantha*,* Senna chacoensis*,* Senna subulata.*	Dry/Sierra Chaco ecotone
Peripheral Dry Chaco	Northern extreme of Bolivian and Paraguayan Dry Chaco	Peripheral Gran Chaco	*Arachis batizocoi*,* Arachis duranensis*,* Chaetocalyx chacoensis*,* Chamaecrista arachyphylla*,* Chaetocalyx chacoensis*,* Chloroleucon chacoense*,* Erythrostemon argentinus.*	Interior Dry Chaco
Low Paraguay–Paraná River Basins	Northeastern Argentina and adjacent eastern Paraguay	Humid Chaco core	*Aeschynomene paraguayensis*,* Arachis correntina*,* Desmodium intermedium*,* Mimosa morongii*,* M. pseudopetiolaris*,* Neltuma nigra* var. *longispinna*,* Tephrosia hassleri*,* Vicia graminea.*	Low Paraguay–Paraná River Basin
Interior Dry Chaco	Western Paraguay and Adjacent Argentina	Interior Dry Chaco	*Vachellia curvifructa*,* Senegalia emilioana*,* Denysophyton stuckertii*,* Dolichopsis paraguariensis*,* Neltuma kuntzei*,* Neltuma nuda*,* Neltuma rojasiana*,* Neltuma sericantha*,* Stylosanthes recta.*	Interior Dry Chaco

**Fig. 4:**
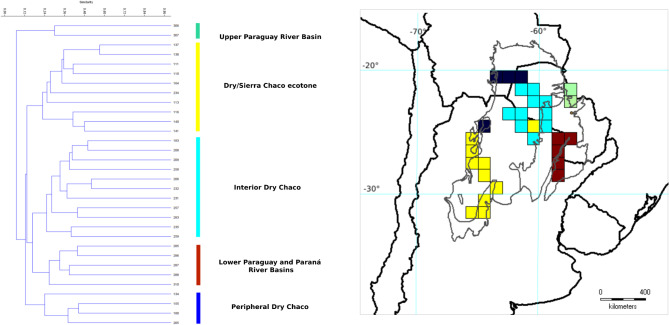
Cluster Analysis and mapping of Centers of Endemism (CEs) by means Unweighted Pair Group Method with Arithmetic Mean (UPGMA) of the Gran Chaco ecoregion based on endemic and highly restricted Fabaceae, calculated with the Jaccard similarity index. This analysis includes only cells with nine or more occurring taxa. Grey thin lines delimitate the three subregions of the Gran Chaco ecoregion.

The UPGMA analysis including only the cells summing 12 or more taxa showed 5 clusters (Centers of Endemism) distributed in 30 cells: (1) Upper Paraguay River Basin; (2) Dry/Sierra Chaco ecotone; (3) Interior Dry Chaco; (4) Low Paraguay–Paraná River Basin; 6) Peripheral Dry Chaco (Table 5; Fig. 4).

**Fig. 5:**
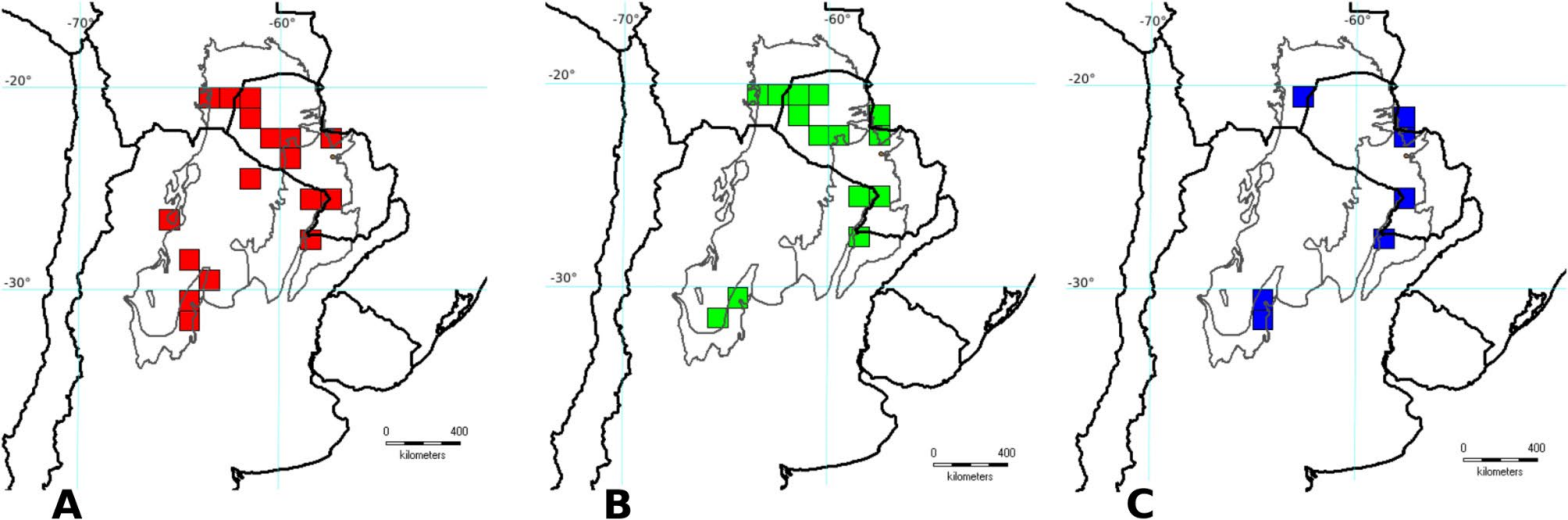
Centers of Endemism in the Gran Chaco ecoregion, based on different Endemicity Richness indexes in grid cell size of 1° × 1°. Left to right: (**A**) Endemic Richness; (**B**) Weighted Endemic Richness; (**C**) Restricted Endemic Richness. Grey thin lines delimitate the three subregions of the Gran Chaco ecoregion.

The endemic richness indexes showed different numbers of centers of endemism. The Endemic Richness Index showed the highest number of cells and CEs (17 and 10, respectively) and the Relative Endemic Richness showed the lowest (7 and 5, respectively). The three indexes showed cells with possible CEs in the same geographic areas, and they were mainly contained in the CEs (clusters) determined by UPGMA analyses (Fig. 5).

###  Distribution Models

**Fig. 6:**
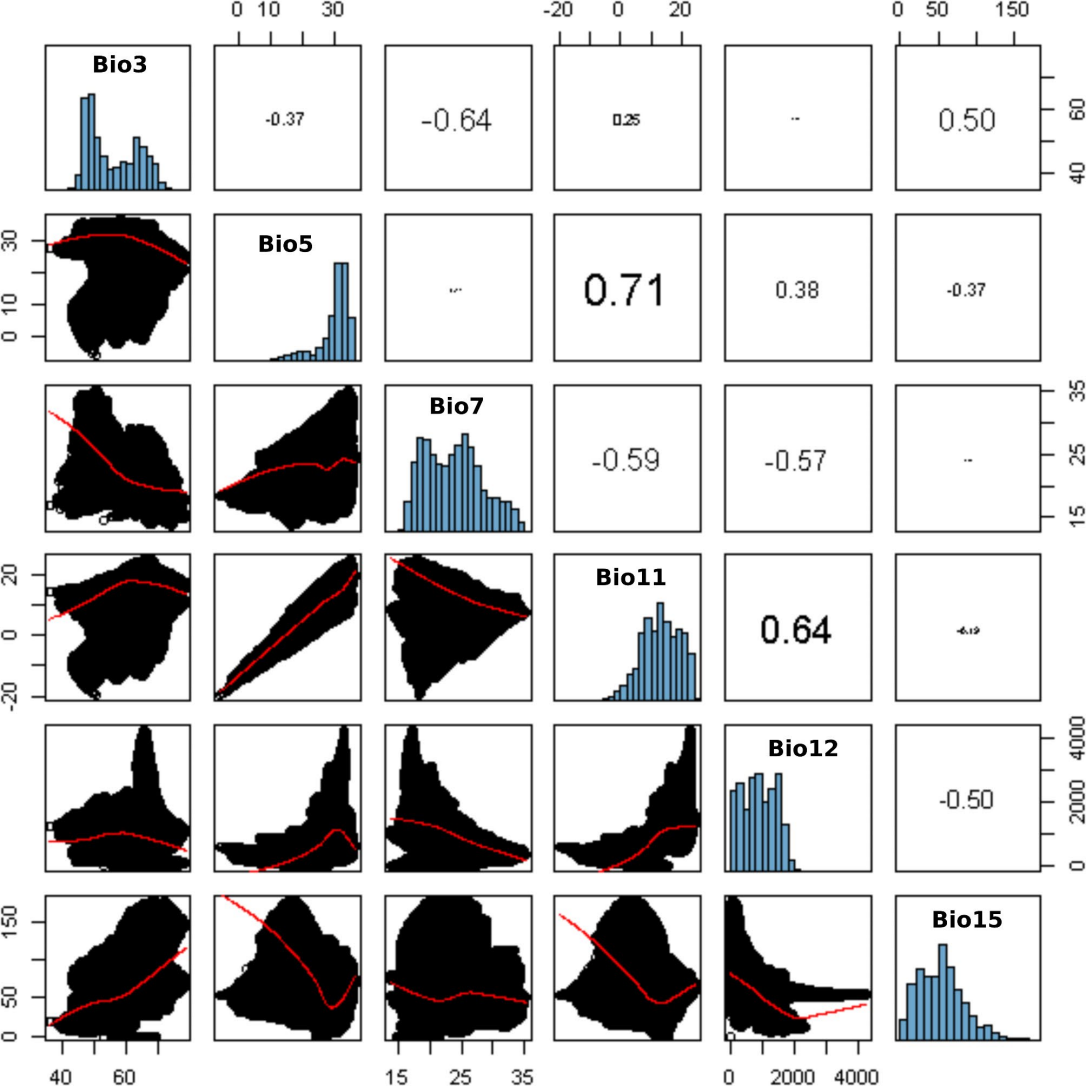
Bivariate analyses (under the diagonal) and Pearson correlation indexes (over the diagonal) of the bioclimatic variables selected for the Chacoan endemic Legumes.

For modelling purposes, we selected endemic Fabaceae with a number of occurrences adequate to generate reliable models. Additionally, in some cases, we included other endemic Fabaceae taxa from the region (Table 6). After evaluating the correlation between all the bioclimatic variables (Suppl. Files: Table 2), we selected six uncorrelated variables related to precipitation and temperature: Isothermality (Bio3), Maximum Temperature of Warmest Month (Bio5), Temperature Annual Range (Bio7), Mean Temperature of Coldest Quarter (Bio11), Annual Precipitation (Bio12), Precipitation Seasonality (Bio15) (Fig. 6). The models with the best performance were, in general terms, those with 1–2 regularization multipliers and Linear feature or Linear–Quadratic feature combination. (Table 7; Suppl. Files: Table 3).

**Table 6:** Selected taxa to model the distribution with MAXENT contributing to the main areas of Endemism and distinctive Center of Endemism detected in the Gran Chaco ecoregion.

Area/Center of Endemism	Taxa
Low Paraguay–Paraná River Basin	*Arachis correntina–Galactia longifolia–Mimosa pseudopetiolaris*
Upper Paraguay River Basin	*Arachis lignosa–Bauhinia hagenbeckii–Neltuma rubriflora*
Interior Dry Chaco	*Arachis batizocoi–Chaetocalyx chacoensis–Lophocarpinia aculeatifolia–Mimosa castanoclada–Piptadeniopsis lomentifera–Neltuma nuda–Senegalia emilioana*
Sierra Chaco	*Dalea elegans–Galactia glaucophylla*
Dry/Sierra Chaco Ecotone	*Adesmia cordobensis–Crotalaria chaco–serranensis–Neltuma flexuosa–Neltuma pugionata–Senna subulata.*

**Table 7:** Evaluation of the performance of MAXENT models for Chacoan Fabaceae endemic taxa.

Endemism area	Species	Feature	Regularization multiplier	AUC Train	CBI Train	AICc	delta AICc
Dry-Sierra Chaco ecotone	Adesmia cordobensis	LQ	1	0.97	0.90	1068.02	0.00
Dry-Sierra Chaco ecotone	Crotalaria chaco-serranensis	LQ	1	0.97	0.87	1041.98	0.00
Dry-Sierra Chaco ecotone	Prosopis flexuosa	LQ	1	0.97	0.88	813.60	0.00
Dry-Sierra Chaco ecotone	Prosopis pugionata	LQ	1	0.96	0.88	566.26	0.00
Dry-Sierra Chaco ecotone	Senna subulata	LQ	1	0.96	0.93	555.98	0.00
Interior Dry Chaco	Arachis batizocoi	LQ	2	0.97	0.18	255.96	0.00
Interior Dry Chaco	Chaetocalyx chacoensis	LQ	2	0.99	0.91	169.71	0.00
Interior Dry Chaco	Lophocarpinia aculeatifolia	LQ	1	0.98	0.65	685.47	0.50
Interior Dry Chaco	Mimosa castanoclada	LQ	2	0.98	0.69	690.38	0.00
Interior Dry Chaco	Piptadeniopsis lomentifera	L	1	0.96	0.74	382.72	0.00
Interior Dry Chaco	Prosopis nuda	L	1	0.97	-0.13	246.59	0.00
Interior Dry Chaco	Senegalia emilioana	LQH	2	0.98	0.64	553.31	0.00
Low Paraguay River Basin	Arachis lignosa	L	3	0.95	-0.07	206.00	0.00
Low Paraguay River Basin	Bauhinia hagenbeckii	LQ	2	0.94	0.25	380.78	0.00
Low Paraguay River Basin	Prosopis rubiflora	LQH	2	1.00	0.78	384.19	0.00
Sierra Chaco	Apurimacia dolichocarpa	L	1	0.91	0.61	307.87	0.00
Sierra Chaco	Dalea elegans	LQ	1	0.99	0.93	823.45	0.00
Sierra Chaco	Galactia glaucophylla	LQ	1	0.99	0.82	806.63	0.00
Upper Paraguay and Paraná Rivers Basin	Arachis correntina	LQ	1	0.99	0.63	659.91	0.00
Upper Paraguay and Paraná Rivers Basin	Galactia longifolia	L	1	0.95	0.65	267.67	0.00
Upper Paraguay and Paraná Rivers Basin	Mimosa pseudopetiolaris	LQ	3	0.98	0.68	209.77	0.00

**Fig. 7:**
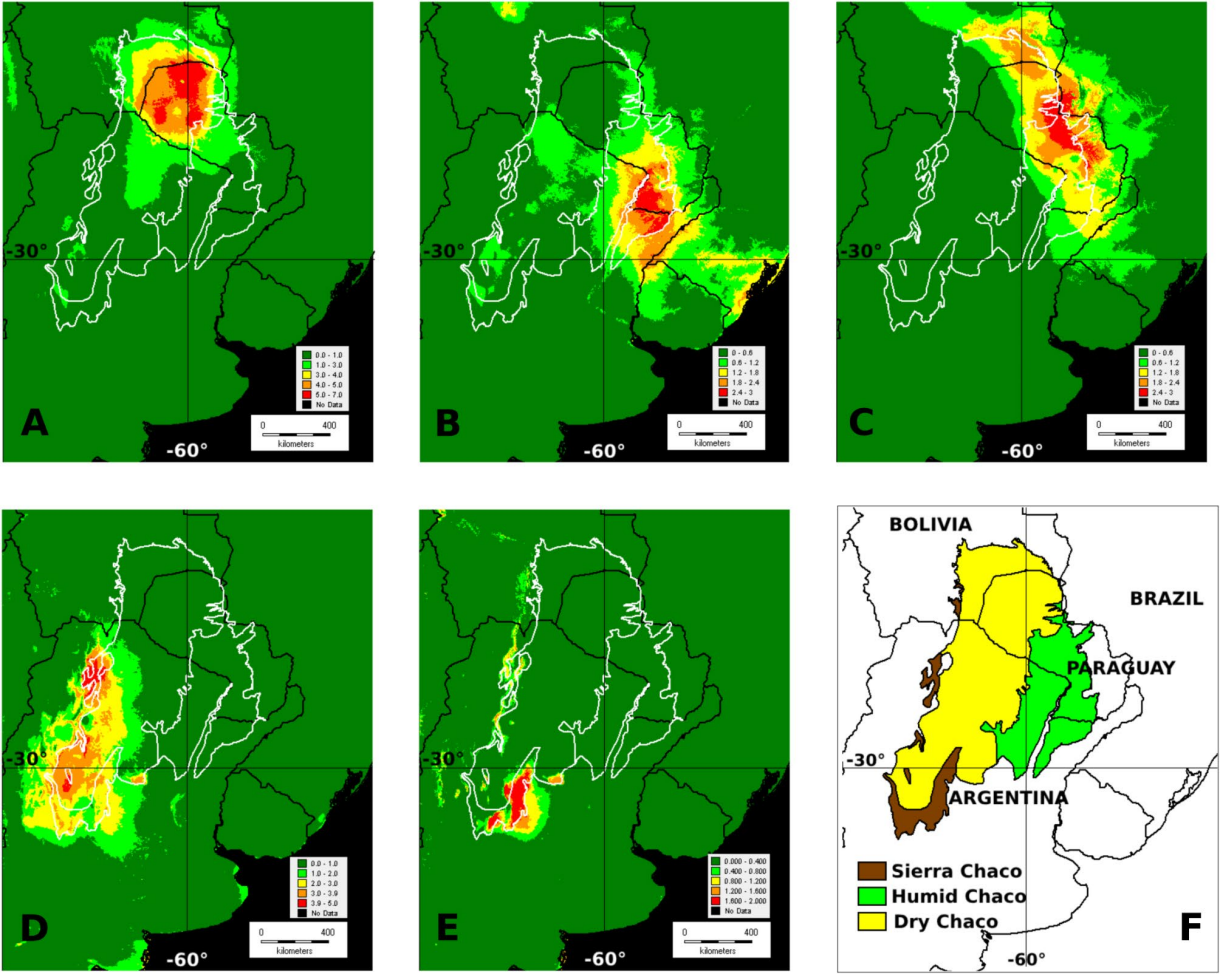
Maps of species richness based on stack of MAXENT distribution models of the taxa contributing to the Areas of Endemism in the Gran Chaco ecoregion. (**A**) Interior Dry Chaco; (**B**) Low Paraguay–Paraná River Basin; (**C**) Upper Paraguay River Basin; (**D**) Dry/Sierra Chaco ecotone; (**E**) Sierra Chaco; (**F**) regionalization of the Gran Chaco ecoregion (Brown: Sierra Chaco; yellow: Dry Chaco; green: Humid Chaco).

We extracted the models and explanatory variables from ENMEval (Fig. 7A–E and F; Table 8; Suppl. Files: Figures 2, 3, 4 and 5). The models of species (*Arachis batizocoi* Krapov. & W. C. Greg., *Chaetocalyx chacoensis* Vanni, *Lophocarpinia aculeatifolia* (Burkart) Burkart, *Mimosa castanoclada* Barneby & Fortunato, *Piptadeniopsis lomentifera* Burkart, *Neltuma nuda* (Schinini) C. E. Hughes & G. P. Lewis, *Senegalia emilioana* (Fortunato & Ciald.) Seigler & Ebinger) and the species-richest area from the stack of the Interior Dry Chaco showed the main occurrence probability in western Paraguay and adjacent regions (Fig. 7A; Suppl. Files: Fig. 2). The species models (*Arachis correntina* (Burkart) Krapov. & W. C. Greg., *Galactia longifolia* Benth. ex Hoehne and *Mimosa pseudopetiolaris* Barneby) and the stack of the Low Paraguay and Paraná River Basins showed the highest probability of occurrence and richness in the Humid Chaco (Fig. 7B; Suppl. Files: Fig. 3D–F). The models of the Upper Paraguay River Basin (*Arachis lignosa* (Chodat & Hassl.) Krapov. & W.C.Greg., *Bauhinia hagenbeckii* and *Neltuma rubriflora* (Hassl.) C. E. Hughes & G. P. Lewis) and their stack concentrated the highest occurrence probability in that region, but also showed a potential region in eastern Bolivia (Fig. 7C; Suppl. Files: Fig. 3A–C); those from the Dry–Sierra Chaco ecotone (*Adesmia cordobensis* Burkart, *Crotalaria chaco-serranensis* H. G. Bach & Fortunato, *Neltuma flexuosa* (DC.) C. E. Hughes & G. P. Lewis, *Neltuma pugionata* (Burkart) C.E. Hughes and G.P. Lewis and *Senna subulata* (Griseb.) H. S. Irwin & Barneby) were more probable to occur in the western extreme area of the Gran Chaco ecoregion (Fig. 7D; Suppl. Files: Fig. 4A–D). Finally, the species from the Sierra Chaco (*Dalea elegans* Gillies ex Hook., *Galactia glaucophylla* Harms) showed their highest probability of occurrence in the southern extreme of this subregion (Fig. 7E; Suppl. File: Fig. 4E–F).

**Table 8:** Main explanatory bioclimatic variables of models (in percentage) for the representative species in endemism areas from the Gran Chaco ecoregion.

Bioclimatic variable	Interior Dry Chaco						Upper Paraguay River Basin				Low Paraguay/Paraná River Basin			Dry/Sierra Chaco Ecotone				Sierra Chaco		
	Neltuma nuda	Mimosa castanoclada	Arachis batizocoi	Lophorpinia aculeatifolia	Piptadeniopsis lomentifera	Senegalia emilioana	Galactia glaucophylla	Arachis lignosa	Neltuma rubriflora	Bauhinia hagenbeckii	Arachis correntina	Galactia longifolia	Mimosa pseudopetiolaris	Neltuma flexuosa	Neltuma pugionata	Adesmia cordobensis	Crotalaria chacorreanensis	Senna subulata	Dalea elegans	Galactia glaucophylla
Isothermality (BIO3)	0.00	0.00	0.00	05.0	0.00	0.00	18.4	0.00	0.00	0.00	34.00	35.4	06.8	23.9	17.3	16.9	37.49	33.6	11.10	18.43
Maximum Temperature of the Warmest Month (BIO5)	50.0	05.4	0.00	84.2	97.4	00.9	20.1	34.1	9.30	16.3	40.06	36.2	0.00	0.00	02.0	29.1	8.87	18.1	38.42	20.09
Temperature annual Range (BIO7)	0.00	0.00	05.6	04.7	0.00	0.09	0.00	0.00	0.00	03.7	15.90	17.5	15.9	04.7	0.00	01.2	0.00	0.00	2.64	0.00
Mean temperature of Coldest Quarter (BIO11)	36.1	52.0	41.8	0.00	0.00	59.5	10.8	33.1	56.5	29.2	0.00	0.00	45.6	13.0	30.7	15.9	3.14	10.4	18.29	10.78
Annual Precipitation (BIO12)	13.8	42.6	47.9	01.1	0.00	39.6	29.4	0.00	00.4	0.00	6.47	10.9	0.00	56.7	50.0	23.3	6,22	18.6	26.63	29.39
Precipitation Seasonality (BIO15)	0.00	0.00	04.8	05.0	02.6	0.00	21.3	32.00	33.8	50.8	3.56	0.00	31.7	01.7	0.00	13.6	44.29	19.7	2.91	21.29

Different environmental variables explained the distribution models (Table 8). In general terms, the variables related to the temperatures in summer (Maximum Temperature of Warmest Month (BIO5) explained high percentages of the models of three of the species representative of Interior Dry Chaco. Distribution of other species of this Center of Endemism were explained by the Annual Precipitation (BIO12) and the Temperature of the Coldest Quarter (BIO11). In the case of the representative species of the Dry/Sierra Chaco ecotone, Isothermality (BIO3) was a variable explaining a significant percentage of the models, together with Annual Precipitation (BIO12) and Precipitation Seasonality (BIO15). The models of the representative species of the regions of Low Paraguay/Paraná centers of endemism were explained by Isothermality and Maximum Temperature of the Warmest Month. The models of the species contributing for Upper Paraguay Basin (*Arachis lignosa*,* Bauhinia hagenbeckii*,* Neltuma rubrifora*) were explained by the Mean Temperature of Coldest Quarter and Precipitation Seasonality. Finally, the models of the Sierra Chaco, such as *Dalea elegans* and *Galactia glaucophylla*,representative species were mainly explained by the Temperature in the Warmest Month and Annual Precipitation.

### Overlap analysis of ecological niches

The analysis of the ecological niche overlap (Table 9) showed that the Schoener’s *D* inde*x* was mainly moderate to high in the pairs of taxa contributing to the Interior Dry Chaco AE, which ranged mainly from 0.25 to 0.63, excepting for *Arachis batizocoi*., which exhibited no overlapping with the remainder taxa. Similarly, the overlap niche between taxa of the Dry–Sierra Chaco ecotone ranged from 0.19 to 0.42. In the remainder Areas of Endemism, the niche overlap was variable, ranging from no overlapping in highly restricted taxa (for example, *Mimosa pseudopetiolaris* in Low Paraguay–Paraná River Basins) to high niche overlap between *Neltuma rubriflora* and *Arachis lignosa* (Upper Paraguay Basin) and moderate in the case of *Arachis correntina* and *Galactia longifolia* (Low Paraguay and Paraná Basins). In Sierra Chaco, the niche overlap was variable, ranging from 0.09 to 0.53.

**Table 9:** Schoener’s index (*D*) of niche overlap and equivalency test between the main taxa contributing to the detected areas of Endemism (bold letters represent statistically significant values *p* = 0.05).

Area of Endemism	Grid cell size	Contributing species pair	D	Equivalency
Interior Dry Chaco	1° × 1°	*Senegalia emilioana–Arachis batizocoi*	0.18	0.78
		*Senegalia emilioana–Chaetocalyx chacoensis*	0.33	0.68
		*Senegalia emilioana*–*Lophocarpinia aculeatifolia*	0.36	**0.90**
		*Senegalia emilioana*–*Mimosa castanoclada*	0.64	0.70
		*Senegalia emilioana–Piptadeniopsis lomentifera*	0.53	0.78
		*Senegalia emilioana–Neltuma nuda*	0.49	0.65
		*Arachis batizocoi–Chaetocalyx chacoensis*	0.06	**0.96**
		*Arachis batizocoi–Lophocarpinia aculeatifolia*	0.02	**0.96**
		*Arachis batizocoi–Mimosa castanoclada*	0.13	**0.96**
		*Arachis batizocoi–Piptadeniopsis lomentifera*	0.06	0.76
		*Arachis batizocoi–Neltuma nuda*	0.06	**1**
		*Chaetocalyx chacoensis–Lophocarpinia aculeatifolia*	0.17	0.92
		*Chaetocalyx chacoensis–Mimosa castanoclada*	0.45	0.45
		*Chaetocalyx chacoensis–Piptadeniopsis lomentifera*	0.30	0.76
		*Chaetocalyx chacoensis–Neltuma nuda*	0.32	0.63
		*Lophocarpinia aculeatifolia–Mimosa castanoclada*	0.31	**1**
		*Lophocarpinia aculeatifolia–Piptadeniopsis lomentifera*	0.59	**1**
		*Lophocarpinia aculeatifolia–Neltuma nuda*	0.55	0.55
		*Mimosa castanoclada–Piptadeniopsis lomentifera*	0.60	**0.96**
		*Mimosa castanoclada–Neltuma nuda*	0.62	0.57
		*Piptadeniopsis lomentifera–Neltuma nuda*	0.48	0.68
Upper Paraguay River Basin	1° × 1°	*Arachis lignosa–Neltuma rubriflora*	0.48	0.84
		*Arachis lignosa–Bauhinia hagenbeckii*	0.006	**0.98**
		*Bauhinia hagenbeckii–Neltuma rubriflora*	0.006	**0.98**
Low Paraguay–Paraná River Basins	1° × 1°	*Arachis correntina–Galactia longifolia*	0.34	**1**
		*Arachis correntina–Mimosa pseudopetiolaris*	0.08	0.94
		*Galactia longifolia - Mimosa pseudopetiolaris*	0	**1**
Sierra Chaco	1° × 1°	*Dalea elegans–Galactia glaucophylla*	0.40	0.70
		*Dalea elegans –Indigofera kurtzii*	0.09	0.94
		*Dalea elegans–Mimosa cordobensis*	0.53	0.17
		*Galactia glaucophylla–Indigofera kurtzii*	0.23	0.47
		*Galactia glaucophylla–Mimosa cordobensis*	0.42	0.12
		*Indigofera kurtzii–Mimosa cordobensis*	0.34	0.27
Dry/Sierra Chaco ecotone	1.25° × 1.25°	*Adesmia cordobensis–Crotalaria chaco-serranensis*	0.42	0.86
		*Adesmia cordobensis–Neltuma flexuosa*	0.22	**1**
		*Adesmia cordobensis–Neltuma pugionata*	0.40	**0.98**
		*Adesmia cordobensis–Senna subulata*	0.48	**1**
		*Crotalaria chaco-serranensis–Neltuma flexuosa*	0.19	0.76
		*Crotalaria chaco-serranensis–Neltuma pugionata*	0.26	0.70
		*Crotalaria chaco-serranensis–Senna subulata*	0.76	0.43
		*Neltuma flexuosa–Neltuma pugionata*	0.42	0.41
		*Neltuma flexuosa–Senna subulata*	0.29	0.78
		*Neltuma pugionata–Senna subulata*	0.26	0.78

The equivalency index was significant in some pairs of Interior Dry Chaco, with moderate to high overlap in the case of *Lophocarpinia aculeatifolia*,* Senegalia emilioana* and *Mimosa castanoclada*, and almost null overlap in the case of pairs involving *Arachis batizocoi.* In Dry/Sierra Chaco ecotone, the equivalency indexes of pairs *Adesmia cordobensis* were significant and moderate (Table 9).

**Fig. 8:**
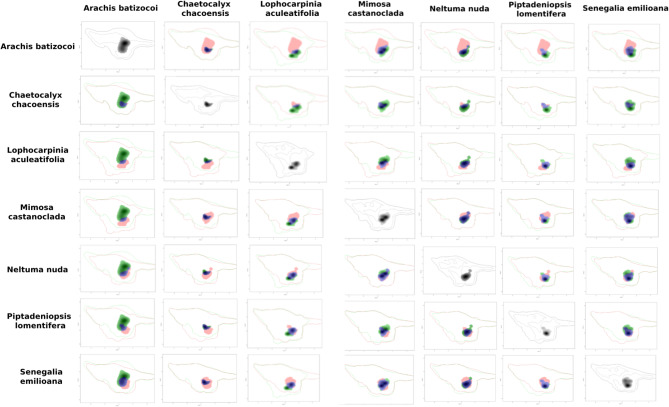
Kernel density occurrences based on grid in the PCA-environmental space (axes 1 and 2) on the representative taxa of Fabaceae in the Gran Chaco ecoregion from Interior Dry Chaco.

**Fig. 9:**
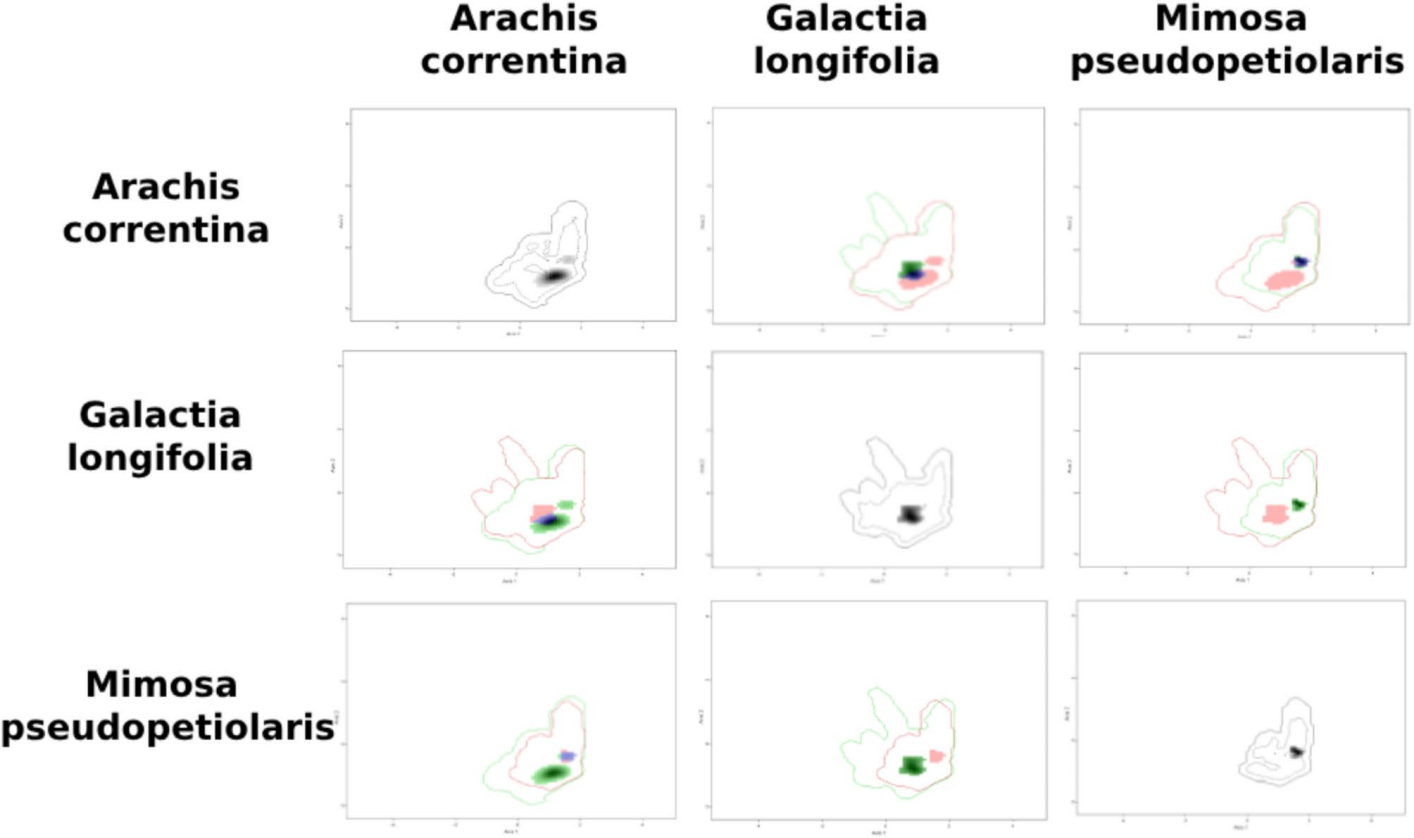
Kernel density occurrences based on grid in the PCA-environmental space (axes 1 and 2) on the representative taxa of Fabaceae in the Gran Chaco ecoregion from Low Paraguay and Paraná River Basins.

**Fig. 10:**
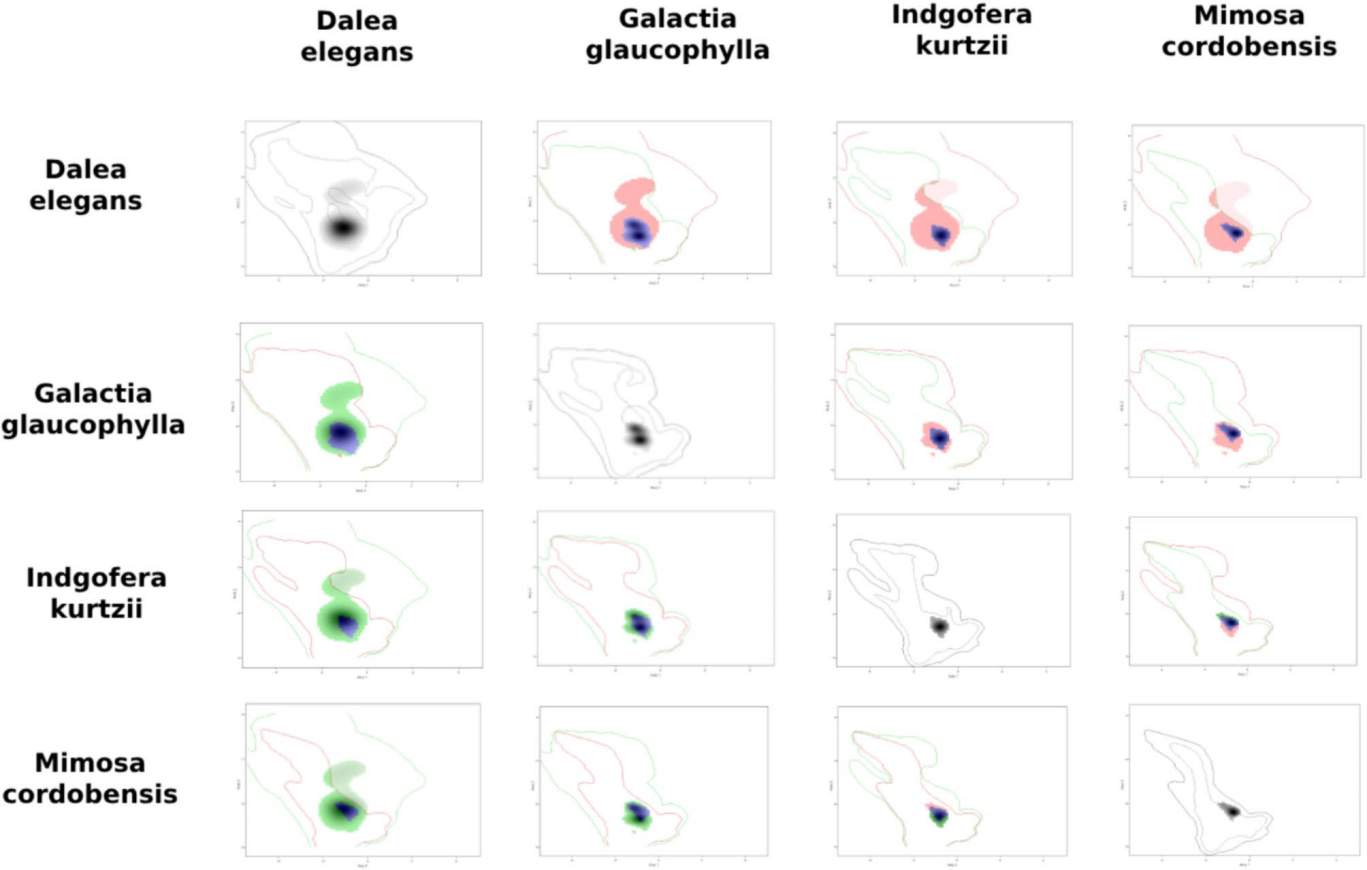
Kernel density occurrences based on grid in the PCA-environmental space (axes 1 and 2) on the representative taxa of Fabaceae in the Gran Chaco ecoregion from Sierra Chaco.

**Fig. 11:**
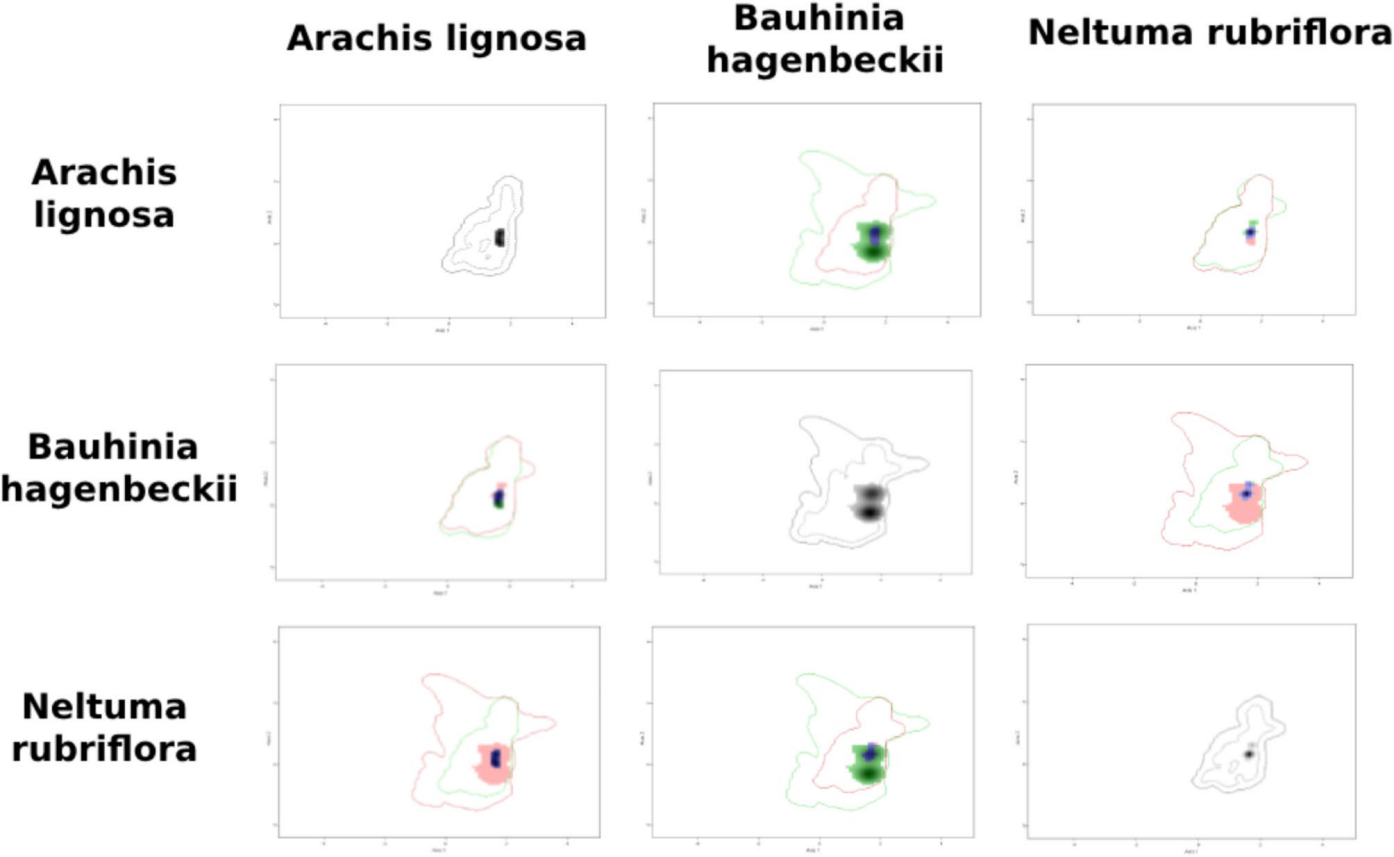
Kernel density occurrences based on grid in the PCA-environmental space (axes 1 and 2) on the representative taxa of Fabaceae in the Gran Chaco ecoregion from Upper Paraguay River Basin.

**Fig. 12:**
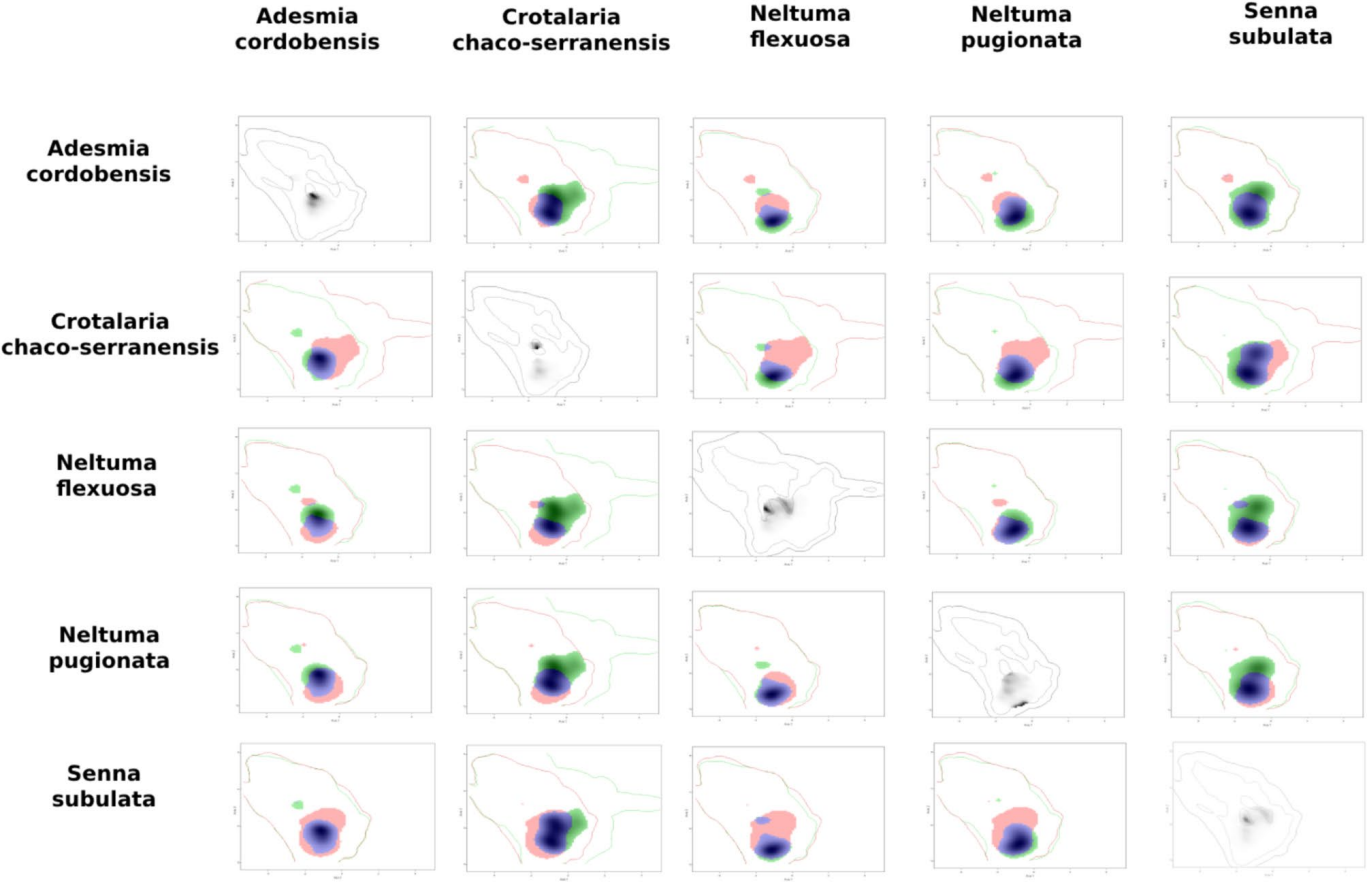
Kernel density occurrences based on grid in the PCA-environmental space (axes 1 and 2) on the representative taxa of Fabaceae in the Gran Chaco ecoregion from Dry–Sierra Chaco ecotone.

The kernel density of the taxa occurrences within each Area of Endemism on the PCA-environmental exhibited similar distribution pattern. They differed between the areas (Figs. 8, 9, 10, 11 and 12). The niches of Interior Dry Chaco were ample, with a relatively high overlap between its taxa (Fig. 8a, b) and the kernel density was concentrated in the positive area of the two first axes in the biplot. The niches of Lower Paraguay and Paraná River Basins were distinctly more concentrated in the positive area of the axis 1 and the negative area of the axis 2; they showed a high overlap between *Arachis correntina* and *Galactia longifolia*, but almost no overlap with *Mimosa pseudopetiolaris* (Fig. 9; Table 8). The niches of the taxa in Sierra Chaco were reduced and concentrated in the negative area of axes 1 and 2, and they showed moderate to high overlap and stability in the majority of taxa pairs (Fig. 10; Table 8). The niches were more restricted in the Upper Paraguay River Basin, in which the densities were more concentrated in the positive area of the Axis 1 and near the null value of the Axes 2; *Arachis lignosa* and *Neltuma rubriflora* showed a high overlap and stability (Fig. 11; Table 8). The niches from Arid Sierra Chaco ecotone were ample and concentrated in the negative area of both axes, similarly to the representative taxa of Sierra Chaco, but their niches seemed more restricted (Fig. 12; Table 8).

The quantification of the niche expansion, stability and unfilling showed high stability between pairs of taxa of the endemism areas, and they were coincident with the *D* niche overlap (Table 10; Suppl. Files: Table 4). The second quartile was ca. 0.5 in almost all of the endemism areas, excepting for Interior Dry Chaco (0.18) and Low Paraguay Basins. The latter also showed a lowest value of third quartile (0.51), comparing with the other areas, where this quartile was higher than 0.8. The presence of extreme values of 0.00 and 1.00 for any of the three indicators was concordant with taxa exhibiting a restricted distribution. The comparison between pairs of taxa from different areas showed that, in general terms, resulted in null or very low values of niche stability (Suppl. Files: Table 4).

**Table 10:** Niche expansion, stability and unfilling in pairs of endemic and restricted taxa of the Gran Chaco ecoregion.

Endemism area	Niche expansion				Niche stability				Niche unfilling			
	Min	Q25	Q75	Max	Min	Q25	Q75	Max	Min	Q25	Q75	Max
Interior Dry Chaco	0.00	0.17	0.59	0.88	0.12	0.22	0.83	1.00	0.00	0.06	0.59	0.88
Dry-Sierra Chaco ecotone	0.04	0.17	0.48	1.00	0.00	0.54	0.83	0.96	0.02	0.14	0.48	1.00
Low Paraguay and Paraná River Basins	0.14	0.48	1.00	1.00	0.00	0.00	0.51	0.86	0.14	0.48	1.00	1.00
Upper Paraguay Basin	0.01	0.06	0.48	0.82	0.18	0.52	0.86	0.99	0.01	0.06	0.47	0.82
Sierra Chaco	0.00	0.00	0.47	0.71	0.29	0.52	1.00	1.00	0.00	0.00	0.58	0.71

References: Min = Minimum value; 0.25 = Quartile; 0.75 = 3th Quartile; Max = Maximum value.

## Discussion

### Areas of endemism

We found different Chacoan Areas and Centers of Endemism. The consensus areas of endemism can be attributed to different factors: presence of microhabitats generated by orography (Sierra Chaco), boundaries between ecoregions (Dry/Sierra Chaco ecotone) or harsh extreme environmental conditions (in the case of Interior Dry Chaco). In contrast to our current detection of several Areas of Endemism, other previous works detected only a single significant area of endemism in Argentina comprising the Gran Chaco plains^28^.

Although our study is limited to one family of plants, we also could demonstrate in recent works that Fabaceae is a good indicator to define ecoregions in tropical and subtropical forest areas in the world^45–47^). In fact, we detected areas largely known as potential Chacoan subregions, such as Arid Chaco^48^. It suggests that the study of the endemism distribution at fine scale can adequately identify areas with common patterns of historical biogeography, and, possibly also, potential centers of speciation for the biota.

Some of the Endemism Areas in Gran Chaco showed partial overlapping and floristic differences, varying its species, especially in the westernmost and easternmost extremes of the ecoregion. It is interesting to point out that the overlapping patterns suggested that different environments are in the same cells^29^. This fact suggests that NDM/VNDM outperforms the hierarchical methods, which are commonly used in biogeography. At regional scale, the 1° × 1° grid cell size shows to be adequate to detect AEs, but larger cells (1.25° × 1.25°) still recovered new Areas of Endemism. We observed that larger cell sizes (more than 1.25° × 1.25°) might be not adequate for Areas of Endemism analyses, given the complexity of the Gran Chaco in the western region, where it is possible to find mosaic of environments, ecotones and pronounced gradients of rainfall and temperatures. On the other hand, we found that grid cells smaller than 1° × 1° recovered very restricted Areas of Endemism, possibly because of the presence of several undersampling sectors.

### Centers of endemism

We identified centers of endemism based on different endemic richness indexes and the Jaccard similarity distances. However, UPGMA based on cells including at least 12 taxa were the most informative. It allowed us to discover some nexus between the richest cells, the Areas of Endemism, and potential biogeographic divisions. In addition, UPGMA allowed us to detect the largest number of Centers of Endemism and rich-endemic cells among all methods here used. Although the Centers of Endemism are not directly related to biogeographical units and its detection is more focused to implement conservation policies^49^, recovering cells with CEs by means of UPGMA could give more biogeographic information than indexes by means of identifying areas with common endemism.

Previous authors^14^ pointed out that the identification of Centers of Endemism, when these analyses are carried out with a larger analysis constrained by biogeographic region, would ensure that these centers are their “core areas”. It would lessen the possibility of conflicts between both types of biogeographic units. The cluster analysis by means of UPGMA gave us adequate information to infer that Centers of Endemism are related to some biogeographic units; although some of them are relatively known in the past (for example, the Sierra Chaco or Dry Chaco^10^), other can be only visualized by our analyses; they would represent a starting point for new proposals of regionalization. It is a challenging topic to develop in further studies integrating more key families of the native Chacoan flora.

In the Gran Chaco ecoregion, we detected some cells configuring small Centers of Endemism. They were from Paraguayan Dry Chaco, the Brazilian–Paraguayan border of Humid Chaco, and the southern portion of Sierra Chaco. These areas would be “hotspots” of biodiversity. At present, neither the Gran Chaco nor larger parts of it are recognized as global “hotspot” areas^50^. However, there are some examples of recently recognized hotspots, such as the North American Coastal Plains^51^ where some assumptions such as the climate and environmental homogeneity and the erroneous climax vegetation were revised. The Gran Chaco could be also subject to similar fallacies, but more probably, undersampling and lack of public registers of biodiversity could be influencing that classification. Some potential areas of Argentine Chaco appear also as Centers of Endemism in our analyses and could be candidates for hotspots, such as the “Impenetrable” area^52^, near the Paraguayan border, as well as the Dry/Sierra Chaco ecotones. These regions clearly require intensive efforts to document their biota.

### Biogeographic issues

The cluster analysis revealed biogeographic patterns that are similar to the Analysis of Endemism. The analysis of all grid cells showed areas related to particular ecosystems described in the literature. The Peripheral Dry Chaco mostly coincided with the sandy area in northern Paraguay and adjacent Bolivia, including a mosaic of Chacoan and SDTF plant species. The Marginal Seasonally Dry Forest area was characterized by the dominance of several species that we previously identified as Seasonally Dry Tropical Forests and Cerrado elements, although they also were endemic or highly restricted to the region^10,53^). Coincidently, it has been observed that the formations of northern Paraguayan Chaco represent transitions from the Gran Chaco to the Chiquitania and Pantanal formations^54^. The Dry Chaco foothills and Dry Valleys are also dominated by a blend of SDTF and Chacoan elements. The Chaco-Espinal ecotone exhibited species of Chacoan lineage that were found in both ecoregions, such as *Neltuma affinis* (Spreng.) C.E. Hughes & G.P. Lewis. The Dry/Sierra Chaco Ecotone comprised an ample region with a mix of species exhibiting Chacoan lineages but mainly exclusive of this sector of the ecoregion.

Regarding the detection of endemism areas, previous studies have revealed that in the Gran Chaco plains of Argentina a large Area of Endemism it has been detected; it comprised the Dry and Humid Chaco, and certain parts of the Sierra Chaco^28^. This result was based on a large and extensive database of animals and plants. In our particular case, exclusively using the endemic and restricted Fabaceae we could retrieve different Endemism Areas; some of them encompassed areas within each subregion of Gran Chaco while others also encompassed ecotones between them.

For instance, the Area of Endemism that we called “Sierra Chaco” comprised a set of cells almost entirely included in the Sierra Chaco subregion^10^ in its southernmost portion. The taxa contributing to this Area of Endemism are highly restricted, with reduced ecological niches but a relatively high degree of overlapping between them. The variables explaining better the distribution of their endemisms are related to summer temperatures, which are lower than the surrounding areas, and the annual precipitations, which are significantly higher than the adjacent areas. These are direct effects of altitude. The ecological distinction can be explained by the presence of intricate environmental heterogeneity leading to high biological diversity in Andes and surrounding areas, which is partly explaining by tectonics and by its perpendicularity to the atmospheric circulation patterns^55^. In mountainous environments, such as Sierra Chaco, speciation processes (natural selection, genetic drift, related to isolation of populations) could explain the notable diversity related to surrounding areas^56^.

In our study, even the most restrictive techniques recovered some Centers of Endemism in the Sierra Chaco area. Interestingly, variability in altitude and topography and the consequent climatic and edaphic variations in small areas of this region determined the existence of the recently recognized “Comechingones” phytogeographic province^57^. Since it consisted of cold temperate grasslands having a different lineage than the Chacoan ones^58,59^), its biota has been not considered for the present study, unless the common species with it. In this work, we assessed the flora reaching up to 1,700 m a.s.l., in concordance with previous criteria of upper limit of Sierra Chaco^10,60^).

In Dry Chaco, we retrieved several Areas of Endemism from the analyses based on the 1°×1° and, in one case, on the 1.25°×1.25° cells. The consensus area for all these areas in the interior Dry Chaco is the central western Paraguay plain, but extending in adjacent Bolivia and Argentina. This area was clearly defined here by several xerophytic, endemic Fabaceae, some of them belonging to monotypic genera, such as *Mimozyganthus* Burkart and *Lophocarpinia* Burkart, which were largely known as endemisms of the Gran Chaco^61,62^). The variables explaining the potential distribution of the endemic taxa are related to the summer temperatures and, secondarily, to the summer rainfall. Consequently, these taxa seem to be well-adapted to extreme conditions of high temperature and low precipitation of Interior Dry Chaco. Some of their ecological niches are convergent and the niche overlap was moderate to high, configuring a large area of potential speciation for Legumes in the Gran Chaco ecoregion.

In the Humid Chaco (eastern portion of the Gran Chaco ecoregion), the analysis of endemism areas detected at least two main consensus areas: Upper Paraguay River Basin, and Low Paraguay–Paraná River Basins. The first one comprised the Brazilian Chaco and the adjacent Paraguayan Chaco, and consisted of a mix of Seasonally Dry Tropical Forests, Chacoan formations and remnants of Cerrado^10^. The existence of ecotones between these ecoregions, as well as the presence of specialized habitats (such as the calcareous outcrops on the left margin of the Paraguay River) could explain the concentration of Fabaceae endemisms.

In this area, our analyses show that the potential distribution of contributing species inferred by the predictive models is mostly configured by seasonal rainfall variability and the winter temperature. According to these variables, this area represents a thermal and rainfall transition between the ecoregions of Atlantic Paraná Rain Forests and Humid Chaco^12^. This subtropical strip is transitional between the first one, with precipitations mainly in all seasons, and the second one, with precipitations more concentrated in summer. The main taxa contributing to this Area of Endemism, such as *Arachis lignosa*, *Bauhinia hagenbeckii* and *Neltuma rubriflora* have been reported to grow in mosaics of SDTF and Chacoan formations in recent works including field data^63–65^;^66,67^). Especially *A. lignosa* and *N. rubriflora* appear to share similar and convergent ecological niches, becoming good indicators for the presence of this endemism area. In our study, 79 specific taxa of Fabaceae are endemic or highly restricted in the Gran Chaco ecoregion, and, notably, 16% of them, in Brazil, are restricted to the Chacoan formations strongly threatened by increasing deforestation.

The Low Paraguay–Paraná River Basins Area of Endemism is also constituted by a mosaic of SDTF and Chacoan formations. It is another transition between the Paranaense and Humid Chaco ecoregions, with thermal transitions; in fact, summer temperatures are milder than other areas of the Gran Chaco ecoregion. It could explain the presence of many grasslands species, such as *Arachis correntina* or *Galactia longifolia.* Additionally, it includes some topographic accidents in southern Paraguay^53,63^). This diversity of environments might explain the occurrence of endemic or rare taxa. Particularly, the outcrops of Tobatí, as well as other minor hills, have restricted relicts of *cerrado* and grasslands with unique or almost so species, belonging to the genus *Mimosa*^63,68^) or *Aeschynomene* L^69^. These outcrops preserve highly restricted species, such as *Mimosa pseudopetiolaris* Barneby^68^. Other representative taxa are growing outside of these outcrops, such as some species of *Desmodium* Desv^70,71^.

We detected another Area of Endemism in the transition between Dry and Sierra Chaco, but only considering an analysis with large cells (1.25° × 1.25°). This Area was identified by us as “Dry/Sierra Chaco ecotone”. It was found as a different Center of Endemism (CEs) in the hierarchical analyses. This area comprised mosaics of extremely dry plains^72^, salt lakes, and dunes, mixed with hills, where the climate is cooler and humid. The taxa that contributed to this AE are typical of these different environments and intermediate areas^73^, and their ecological niches are delimited by temperature and precipitation variables, such as minimum extreme temperature in winter. This AE has a correspondence with some cells representing CEs throughout the area.

This ecotone is partially located in the previously recognized unit of vegetation called “Chaco Árido”^48,72^). , which mainly involves a transition between forests of *Aspidosperma quebracho–blanco* Schltdl. and steppes^13^. Some of the endemic taxa contributing to the Area of Endemism are *Neltuma flexuosa* and *N. pugionata*, accompanied by other endemic Fabaceae, such *Mimozyganthus carinatus* (Griseb.) Burkart. This unit of vegetation seems to be situated in a mosaic with formations of Sierra Chaco, where other taxa, such as *Adesmia cordobensis* are also contributors to performing the CEs. The presence of floristic elements of Fabaceae in this area, that are common to Sierra Chaco and Arid and Dry Chaco, was previously found by other authors^60^). In addition, it is interesting to point out that this Area of Endemism includes the presence of ecotones between arid and humid ecoregions, such as Monte, Yungas, Puna and Gran Chaco. In the adjacent western area, previous authors have found several Areas of Endemism analyzing the flora of southern Central Andes, which was explained by the diversity in altitude and rainfalls^27^.

### Conservation issues

Since the Gran Chaco ecoregion is suffering one of the highest deforestation rates in the world from the 2000’decade^74^, all information contributing to elucidate priority areas of conservation is urgently demanded. Recently, some works have established priority areas based in the presence of endemic vertebrates^75^. These areas coincided with our detected sectors in the interior Dry Chaco AE/CEs, the Low Paraguay–Paraná River Basins AE/CE, and the Sierra Chaco and Dry/Sierra Chaco Ecotone Areas of Endemism. Interestingly^76^, some authors have observed that endemic richness of plants and vertebrates are generally correlated. Therefore, further integration of information from endemic taxa could have significant implications for delimitation of new protected areas and extensions of those already established or, even better, the consolidation of buffer areas surrounding reserves and parks.

It is especially interesting that the Interior Dry Chaco Area of Endemism represented the most visible area of endemism and included centers rich in endemics. It coincided with the areas of the Gran Chaco that has suffered the highest rates of forest loss from 2000^77,78^).

Our studies about ecological niches of indicator taxa allowed us to infer that delimitation of areas and centers of endemism in which their taxa exhibited possible ecological niche divergence requires more detailed studies to conservation policies. It is because of their biota seems to be ecologically complex; it might be related to the presence of microhabitats, ecotones or mosaics of habitat. The most visible case of this work is the Sierra Chaco area/center of endemism, whose complex topography might be causing specialized habitats^58^ via ecological isolation, climatic and edaphic variations and the ingression of Andean elements of the flora^10^).

The northwestern area of the Gran Chaco requires an intensive sampling and complementary studies of the biota to improve the regionalization proposals, because of the complexity generated not only by the topography, but also by the presence of mosaics of different vegetal formations^79^. They are related to slope orientation and altitude triggering great differences in annual precipitation and temperatures. In our analyses, the northern portions of Sierra Chaco were detected only at large-scale analyses in both, AE and CE methodologies. For conservation purposes, an exhaustive sampling in this area is imperative, in order to generate adequate plans to preserve the biological diversity.

There are currently few areas with Chaco formations in conservation units; this increases the risk of extinction of endemic species. Indeed, the natural resources of the Gran Chaco areas along its extension are used by humans for different purposes, such as fishing, crafts, religious cults, construction, medicinal uses which strengthens their relationship with the Chaco formations. Public policies are urgently needed in areas with CEs, especially in some cells of the Chaco such as the Paraguayan Dry Chaco, the Brazil-Paraguay border of the Humid Chaco and the southern portion of the Chaco mountains.

## Electronic supplementary material

Below is the link to the electronic supplementary material.

Supplementary Material 1

Supplementary Material 2

Supplementary Material 3

Supplementary Material 4

Supplementary Material 5

Supplementary Material 6

Supplementary Material 7

Supplementary Material 8

Supplementary Material 9

## Data Availability

The majority of data are available within the manuscript and supplementary information files, in the Online Appendix. Original matrices, distribution data, singular areas of endemisms, codes and remaining data are available in Zenodo and from the corresponding author on request.
