# Supplementary figures and images for: Identifying areas and centers of endemism in the Gran Chaco with Fabaceae as a diversity indicator
Source: Sci Rep. 2025 Mar 20;15:9572. doi: 10.1038/s41598-025-90091-3 (PMC11926246; doi:10.1038/s41598-025-90091-3)

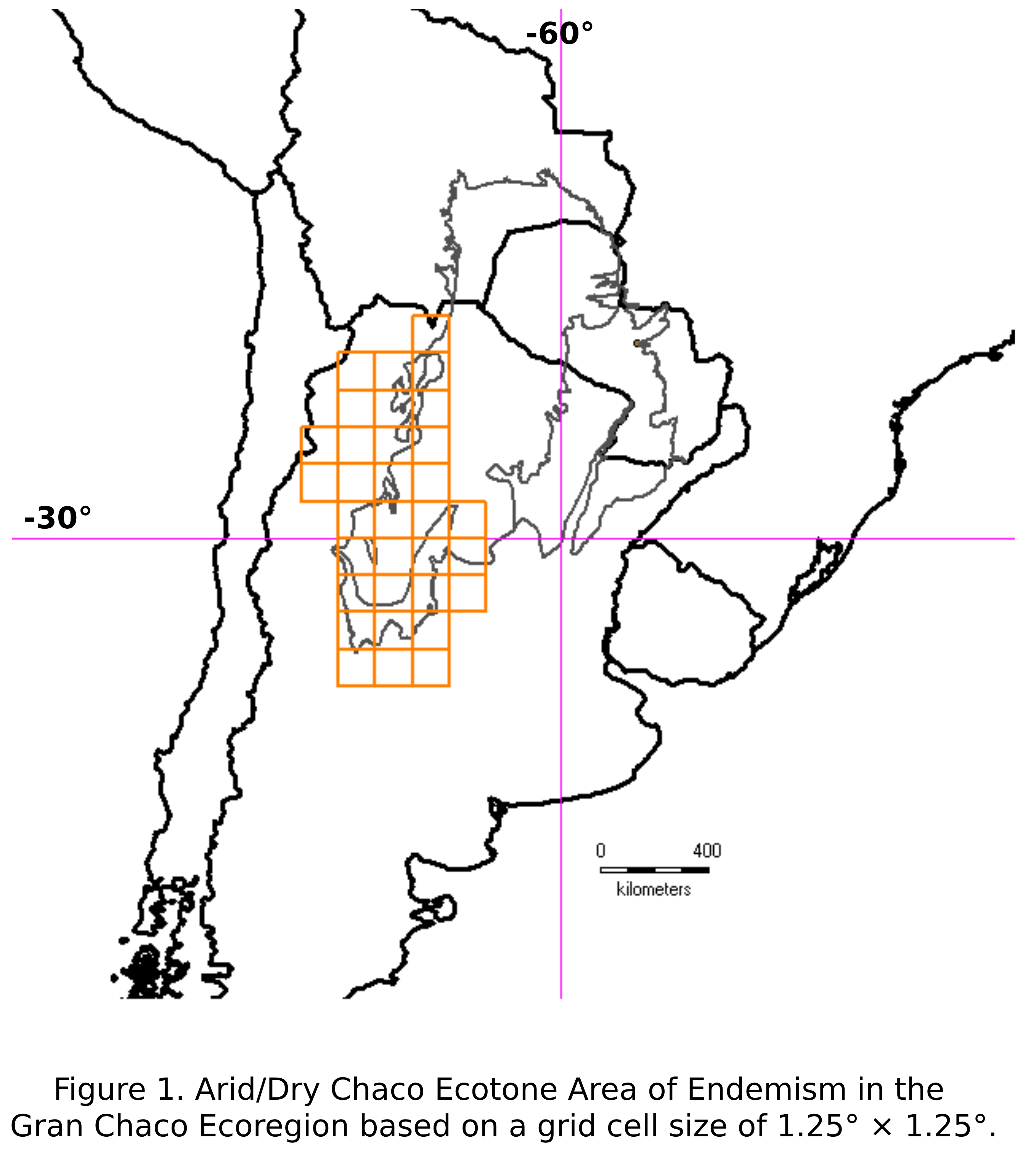

Supplement: Supplementary file 1 — Supplementary Material 1 [file 41598_2025_90091_MOESM1_ESM.tiff]

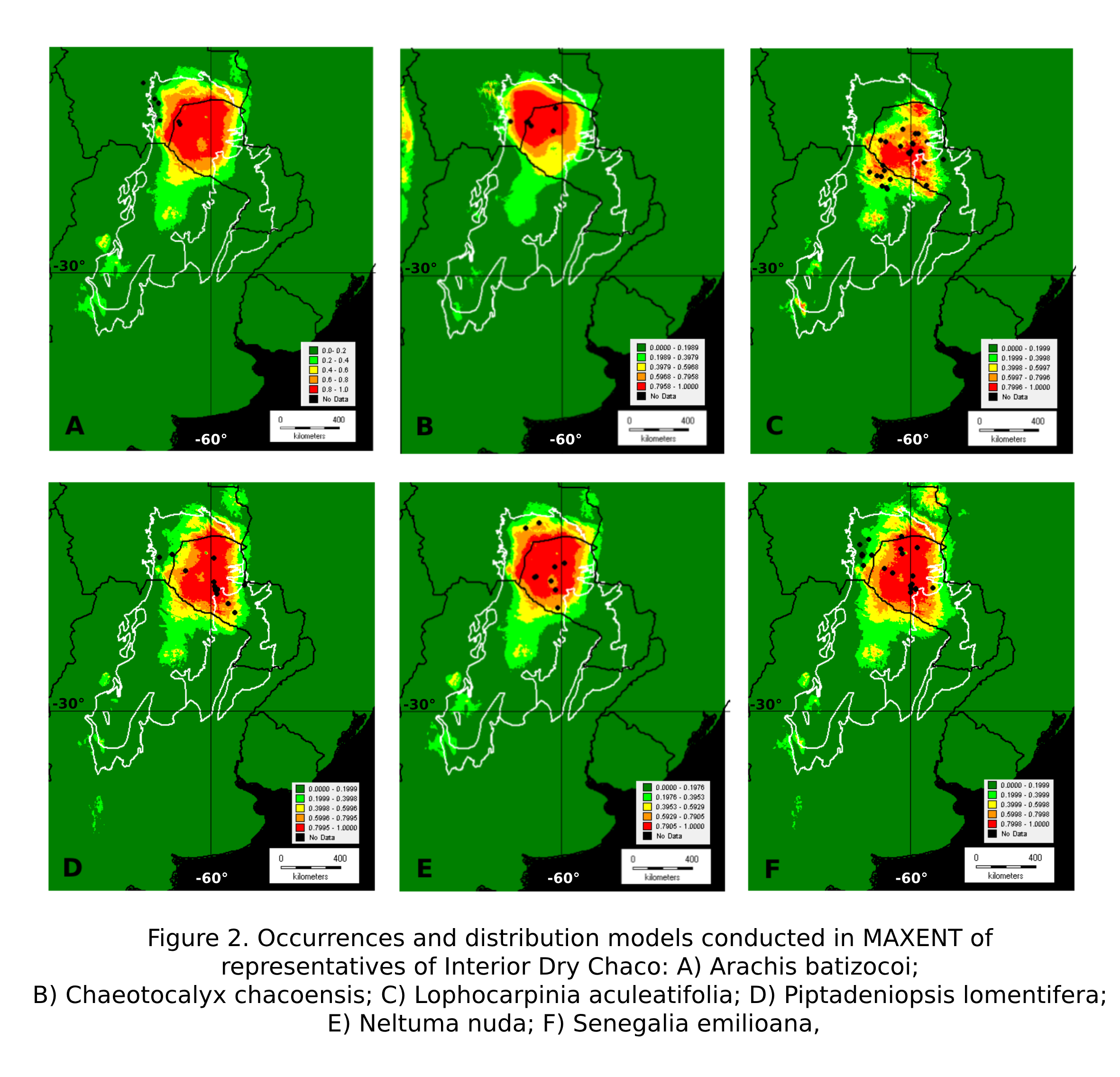

Supplement: Supplementary file 2 — Supplementary Material 2 [file 41598_2025_90091_MOESM2_ESM.tiff]

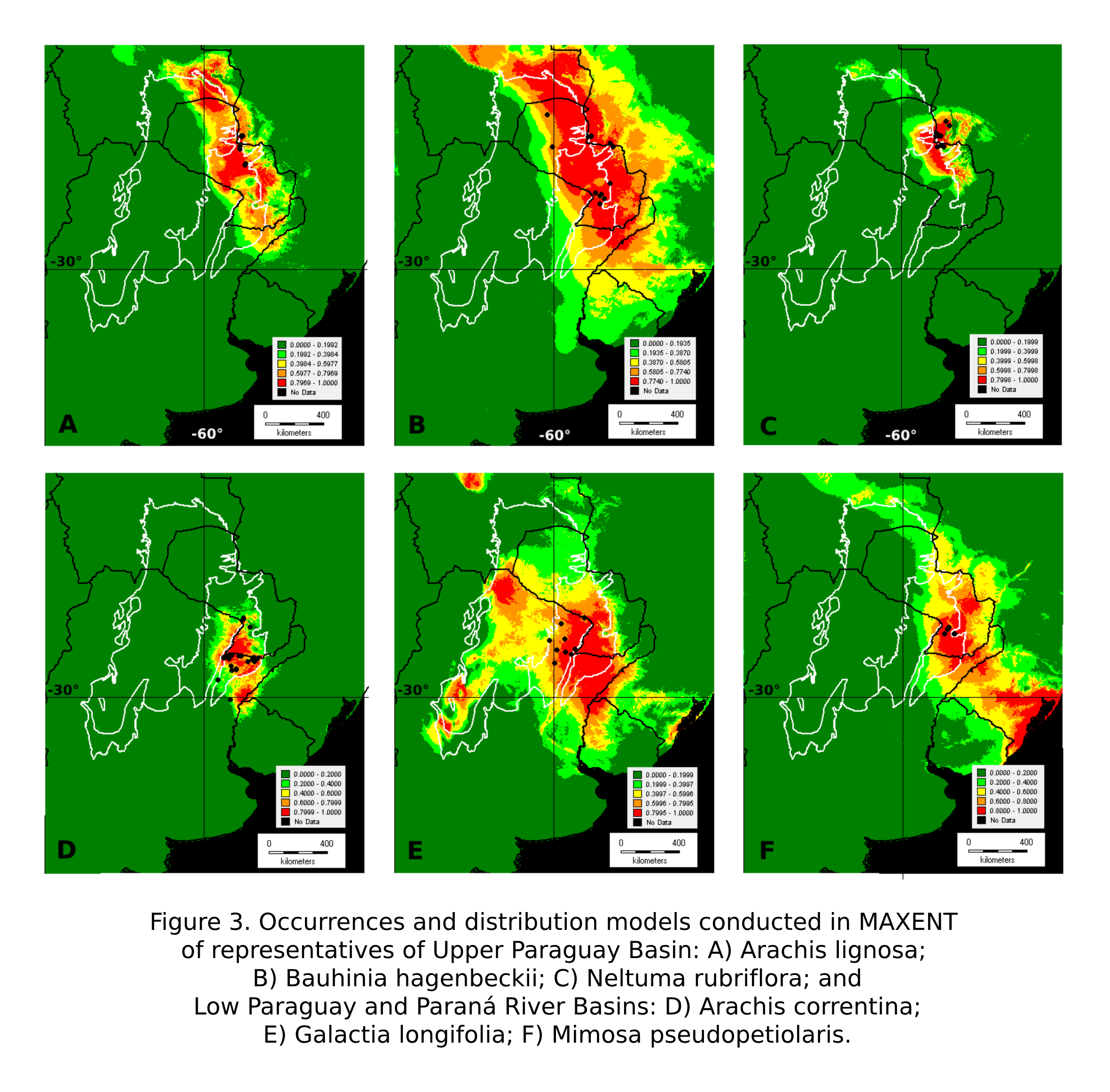

Supplement: Supplementary file 3 — Supplementary Material 3 [file 41598_2025_90091_MOESM3_ESM.tiff]

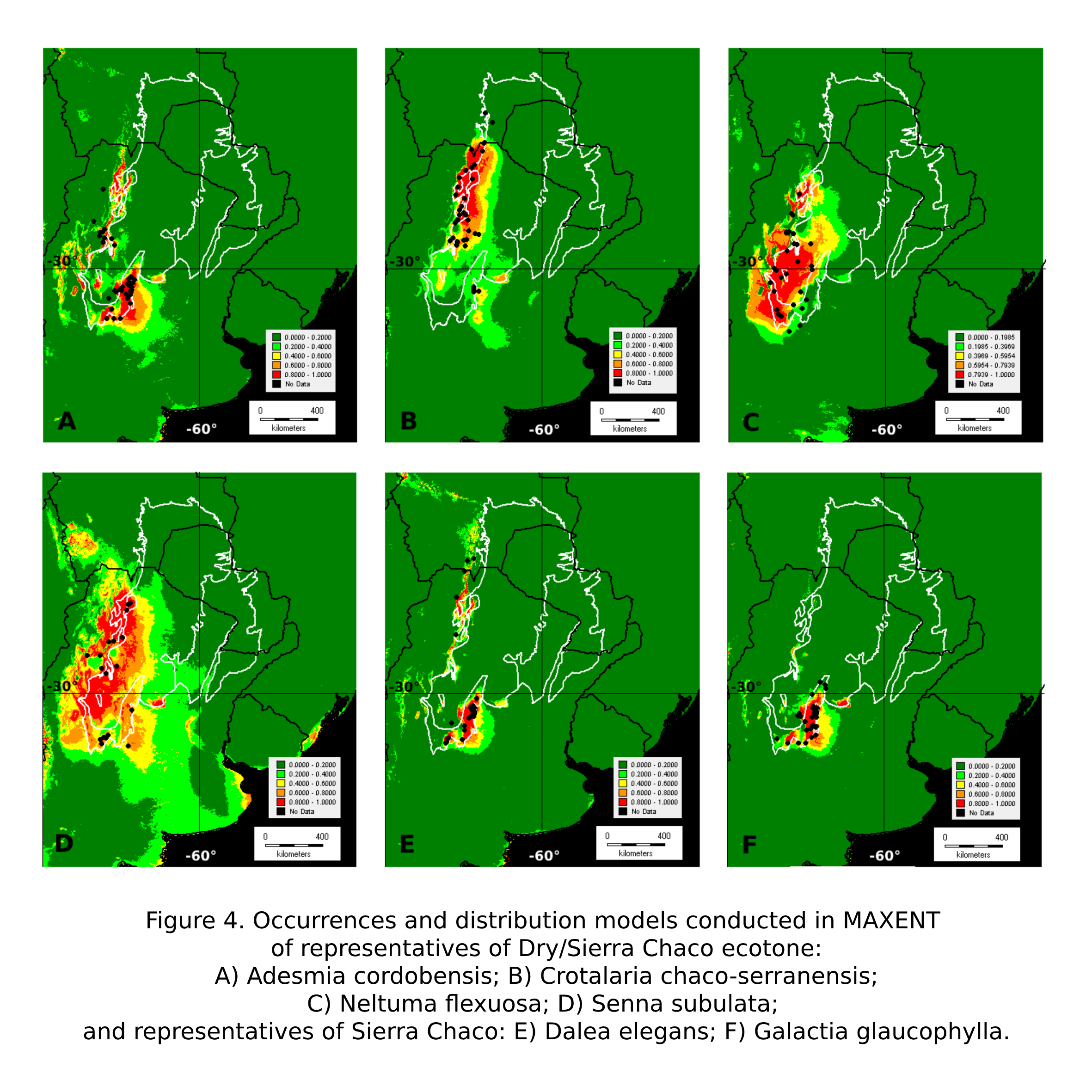

Supplement: Supplementary file 9 — Supplementary Material 9 [file 41598_2025_90091_MOESM9_ESM.tiff]
