# Supplementary material for: Identifying areas and centers of endemism in the Gran Chaco with Fabaceae as a diversity indicator
Source: Sci Rep. 2025 Mar 20;15:9572. doi: 10.1038/s41598-025-90091-3 (PMC11926246; doi:10.1038/s41598-025-90091-3)
Supplement: Supplementary file 4 — Supplementary Material 4 [file 41598_2025_90091_MOESM4_ESM.docx]

**Table 1 (Suppl.).** Endemism area, consensus and species contribution to the consensus areas of endemism detected by NDM-VNDM with 1 × 1 grid cell size in the Gran Chaco ecoregion

| **Consensus** | **Endemism area** | **Endemism area (consensus)** | **Species** | **Score** | **Total score** |
| --- | --- | --- | --- | --- | --- |
| 3 | 17 | Lower Paraguay and Paraná River Basins | Mimosa morongii | 0,72 | 2,98 |
| 3 | 17 | Lower Paraguay and Paraná River Basins | Demodium intermedium | 0,52 | 2,98 |
| 3 | 17 | Lower Paraguay and Paraná River Basins | Aeschynomene paraguayensis | 0,67 | 2,98 |
| 3 | 17 | Lower Paraguay and Paraná River Basins | Tephrosia hassleri | 0,35 | 2,98 |
| 2 | 2 | Sierra Chaco | Apurimacia dolichocarpa | 0,25 | 2,18 |
| 2 | 2 | Sierra Chaco | Indigofera kurtzii | 0,44 | 2,18 |
| 2 | 2 | Sierra Chaco | Mimosa cordobensis | 0,62 | 2,18 |
| 2 | 2 | Sierra Chaco | Clitoria cordobensis | 0,88 | 2,18 |
| 2 | 3 | Sierra Chaco | Apurimacia dolichocarpa | 0,50 | 2,89 |
| 2 | 3 | Sierra Chaco | Indigofera kurtzii | 0,69 | 2,89 |
| 2 | 3 | Sierra Chaco | Mimosa cordobensis | 0,96 | 2,89 |
| 2 | 3 | Sierra Chaco | Clitoria cordobensis | 0,75 | 2,89 |
| 2 | 7 | Sierra Chaco | Apurimacia dolichocarpa | 1,00 | 2,50 |
| 2 | 7 | Sierra Chaco | Indigofera kurtzii | 0,45 | 2,50 |
| 2 | 7 | Sierra Chaco | Mimosa cordobensis | 0,48 | 2,50 |
| 2 | 7 | Sierra Chaco | Clitoria cordobensis | 0,57 | 2,50 |
| 2 | 13 | Sierra Chaco | Apurimacia dolichocarpa | 0,83 | 3,14 |
| 2 | 13 | Sierra Chaco | Indigofera kurtzii | 0,83 | 3,14 |
| 2 | 13 | Sierra Chaco | Mimosa cordobensis | 0,81 | 3,14 |
| 2 | 13 | Sierra Chaco | Clitoria cordobensis | 0,67 | 3,14 |
| 2 | 18 | Sierra Chaco | Apurimacia dolichocarpa | 0,50 | 3,04 |
| 2 | 18 | Sierra Chaco | Indigofera kurtzii | 0,93 | 3,04 |
| 2 | 18 | Sierra Chaco | Mimosa cordobensis | 0,89 | 3,04 |
| 2 | 18 | Sierra Chaco | Clitoria cordobensis | 0,71 | 3,04 |
| 1 | 1 | Upper Paraguay Basin | Arachis lignosa | 0,56 | 2,84 |
| 1 | 1 | Upper Paraguay Basin | Arachis microsperma | 0,92 | 2,84 |
| 1 | 1 | Upper Paraguay Basin | Prosopis rubiflora | 0,58 | 2,84 |
| 1 | 1 | Upper Paraguay Basin | Aeschynomene magna | 0,79 | 2,84 |
| 1 | 6 | Upper Paraguay Basin | Arachis lignosa | 0,92 | 2,11 |
| 1 | 6 | Upper Paraguay Basin | Prosopis rubiflora | 0,40 | 2,11 |
| 1 | 6 | Upper Paraguay Basin | Aeschynomene magna | 0,79 | 2,11 |
| 1 | 9 | Upper Paraguay Basin | Arachis lignosa | 0,58 | 2,33 |
| 1 | 9 | Upper Paraguay Basin | Arachis microsperma | 0,44 | 2,33 |
| 1 | 9 | Upper Paraguay Basin | Prosopis rubiflora | 0,38 | 2,33 |
| 1 | 9 | Upper Paraguay Basin | Aeschynomene magna | 0,94 | 2,33 |
| 1 | 15 | Upper Paraguay Basin | Arachis lignosa | 0,71 | 3,10 |
| 1 | 15 | Upper Paraguay Basin | Arachis microsperma | 0,78 | 3,10 |
| 1 | 15 | Upper Paraguay Basin | Prosopis rubiflora | 0,92 | 3,10 |
| 1 | 15 | Upper Paraguay Basin | Aeschynomene magna | 0,69 | 3,10 |
| 1 | 19 | Upper Paraguay Basin | Arachis lignosa | 0,55 | 3,05 |
| 1 | 19 | Upper Paraguay Basin | Arachis microsperma | 0,81 | 3,05 |
| 1 | 19 | Upper Paraguay Basin | Prosopis rubiflora | 0,97 | 3,05 |
| 1 | 19 | Upper Paraguay Basin | Aeschynomene magna | 0,72 | 3,05 |
| 0 | 0 | Dry Chaco | Acacia emilioana | 0,75 | 2,70 |
| 0 | 0 | Dry Chaco | Mimosa castanoclada | 0,68 | 2,70 |
| 0 | 0 | Dry Chaco | Piptadeniopsis lomentifera | 0,63 | 2,70 |
| 0 | 0 | Dry Chaco | Stylosanthes recta | 0,68 | 2,70 |
| 0 | 4 | Dry Chaco | Acacia emilioana | 0,62 | 2,90 |
| 0 | 4 | Dry Chaco | Lophocarpinia | 0,41 | 2,90 |
| 0 | 4 | Dry Chaco | Mimosa castanoclada | 0,58 | 2,90 |
| 0 | 4 | Dry Chaco | Piptadeniopsis lomentifera | 0,61 | 2,90 |
| 0 | 4 | Dry Chaco | Stylosanthes recta | 0,69 | 2,90 |
| 0 | 5 | Dry Chaco | Lophocarpinia | 0,39 | 2,48 |
| 0 | 5 | Dry Chaco | Mimosa castanoclada | 0,38 | 2,48 |
| 0 | 5 | Dry Chaco | Piptadeniopsis lomentifera | 0,48 | 2,48 |
| 0 | 5 | Dry Chaco | Prosopis rojasiana | 0,60 | 2,48 |
| 0 | 5 | Dry Chaco | Stylosanthes recta | 0,63 | 2,48 |
| 0 | 8 | Dry Chaco | Lophocarpinia aculeatifolia | 0,32 | 2,20 |
| 0 | 8 | Dry Chaco | Mimosa castanoclada | 0,37 | 2,20 |
| 0 | 8 | Dry Chaco | Piptadeniopsis lomentifera | 0,39 | 2,20 |
| 0 | 8 | Dry Chaco | Prosopis rojasiana | 0,47 | 2,20 |
| 0 | 8 | Dry Chaco | Stylosanthes recta | 0,65 | 2,20 |
| 0 | 10 | Dry Chaco | Acacia emilioana | 0,67 | 2,55 |
| 0 | 10 | Dry Chaco | Mimosa castanoclada | 0,66 | 2,55 |
| 0 | 10 | Dry Chaco | Piptadeniopsis lomentifera | 0,53 | 2,55 |
| 0 | 10 | Dry Chaco | Stylosanthes recta | 0,70 | 2,55 |
| 0 | 11 | Dry Chaco | Acacia emilioana | 0,55 | 2,70 |
| 0 | 11 | Dry Chaco | Lophocarpinia aculeatifolia | 0,34 | 2,70 |
| 0 | 11 | Dry Chaco | Mimosa castanoclada | 0,59 | 2,70 |
| 0 | 11 | Dry Chaco | Piptadeniopsis lomentifera | 0,51 | 2,70 |
| 0 | 11 | Dry Chaco | Stylosanthes recta | 0,71 | 2,70 |
| 0 | 12 | Dry Chaco | Acacia emilioana | 0,50 | 0,74 |
| 0 | 12 | Dry Chaco | Chaecalyx chacoensis | 0,74 | 0,74 |
| 0 | 12 | Dry Chaco | Mimosa castanoclada | 0,69 | 0,74 |
| 0 | 12 | Dry Chaco | Prosopis nuda | 0,39 | 0,74 |
| 0 | 12 | Dry Chaco | Stylosanthes recta | 0,60 | 0,74 |
| 0 | 14 | Dry Chaco | Arachis batizocoi | 0,44 | 2,61 |
| 0 | 14 | Dry Chaco | Stylosanthes recta | 0,49 | 2,61 |
| 0 | 14 | Dry Chaco | Chaetocalyx chacoensis | 0,78 | 2,61 |
| 0 | 14 | Dry Chaco | Mimosa castanoclada | 0,58 | 2,61 |
| 0 | 14 | Dry Chaco | Prosopis nuda | 0,32 | 2,61 |
| 0 | 16 | Dry Chaco | Acacia emilioana | 0,69 | 3,10 |
| 0 | 16 | Dry Chaco | Mimosa castanoclada | 0,66 | 3,10 |
| 0 | 16 | Dry Chaco | Stylosanthes recta | 0,70 | 3,10 |
| 0 | 16 | Dry Chaco | Prosopis nuda | 0,50 | 3,10 |
| 0 | 16 | Dry Chaco | Piptadeniopsis lomentifera | 0,55 | 3,10 |
| 0 | 20 | Dry Chaco | Acacia emilioana | 0,55 | 2,14 |
| 0 | 20 | Dry Chaco | Mimosa castanoclada | 0,88 | 2,14 |
| 0 | 20 | Dry Chaco | Chaeocalyx | 0,71 | 2,14 |
| 0 | 0 | Dry/Sierra Chaco ecotone | Adesmia cordobensis | 0,62 | 3,31 |
| 0 | 0 | Dry/Sierra Chaco ecotone | Neltuma campestris | 0,66 | 3,31 |
| 0 | 0 | Dry/Sierra Chaco ecotone | Neltuma flexosa | 0,70 | 3,31 |
| 0 | 0 | Dry/Sierra Chaco ecotone | Neltuma pugiopnata | 0,69 | 3,31 |
| 0 | 0 | Dry/Sierra Chaco ecotone | Senna subulata | 0,64 | 3,31 |
| 0 | 1 | Dry/Sierra Chaco ecotone | Adesmia cordobensis | 0,62 | 3,31 |
| 0 | 1 | Dry/Sierra Chaco ecotone | Neltuma campestris | 0,70 | 3,31 |
| 0 | 1 | Dry/Sierra Chaco ecotone | Neltuma flexosa | 0,70 | 3,31 |
| 0 | 1 | Dry/Sierra Chaco ecotone | Neltuma pugiopnata | 0,69 | 3,31 |
| 0 | 1 | Dry/Sierra Chaco ecotone | Senna subulata | 0,64 | 3,31 |
