# Supplementary material for: Identifying areas and centers of endemism in the Gran Chaco with Fabaceae as a diversity indicator
Source: Sci Rep. 2025 Mar 20;15:9572. doi: 10.1038/s41598-025-90091-3 (PMC11926246; doi:10.1038/s41598-025-90091-3)
Supplement: Supplementary file 5 — Supplementary Material 5 [file 41598_2025_90091_MOESM5_ESM.docx]

**Table 2A.** Pearson correlation coefficient of the 19 bioclimatic variables of Wordclim 2.0

| Bioclimatic variables | 1 | 2 | 3 | 4 | 5 | 6 | 7 | 8 | 9 | 10 | 11 | 12 | 13 | 14 | 15 | 16 | 17 | 18 | 19 | **mean** |
| --- | --- | --- | --- | --- | --- | --- | --- | --- | --- | --- | --- | --- | --- | --- | --- | --- | --- | --- | --- | --- |
| 1 | 1.00 | -0.52 | 0.04 | -0.26 | 0.86 | 0.96 | -0.40 | 0.90 | 0.87 | 0.94 | 0.97 | 0.60 | 0.63 | 0.22 | -0.26 | 0.65 | 0.25 | 0.67 | 0.25 | 18.03 |
| 2 | -0.52 | 1.00 | 0.11 | 0.33 | -0.26 | -0.69 | 0.69 | -0.33 | -0.62 | -0.44 | -0.57 | -0.70 | -0.56 | -0.59 | 0.55 | -0.58 | -0.60 | -0.59 | -0.64 | 13.05 |
| 3 | 0.04 | 0.11 | 1.00 | -0.89 | -0.37 | 0.15 | -0.64 | -0.09 | 0.22 | -0.27 | 0.25 | 0.05 | 0.28 | -0.23 | 0.50 | 0.27 | -0.20 | 0.20 | -0.16 | 55.60 |
| 4 | -0.26 | 0.33 | -0.89 | 1.00 | 0.25 | -0.43 | 0.90 | -0.06 | -0.47 | 0.08 | -0.49 | -0.37 | -0.53 | -0.05 | -0.27 | -0.53 | -0.08 | -0.46 | -0.12 | 391.32 |
| 5 | 0.86 | -0.26 | -0.37 | 0.25 | 1.00 | 0.72 | 0.11 | 0.87 | 0.62 | 0.97 | 0.71 | 0.38 | 0.35 | 0.14 | -0.37 | 0.37 | 0.16 | 0.41 | 0.13 | 29.68 |
| 6 | 0.96 | -0.69 | 0.15 | -0.43 | 0.72 | 1.00 | -0.61 | 0.80 | 0.92 | 0.85 | 0.98 | 0.70 | 0.70 | 0.33 | -0.33 | 0.72 | 0.37 | 0.73 | 0.38 | 5.80 |
| 7 | -0.40 | 0.69 | -0.64 | 0.90 | 0.11 | -0.61 | 1.00 | -0.15 | -0.62 | -0.11 | -0.59 | -0.57 | -0.61 | -0.31 | 0.05 | -0.62 | -0.34 | -0.58 | -0.40 | 23.88 |
| 8 | 0.90 | -0.33 | -0.09 | -0.06 | 0.87 | 0.80 | -0.15 | 1.00 | 0.62 | 0.91 | 0.83 | 0.42 | 0.48 | 0.06 | -0.14 | 0.50 | 0.09 | 0.56 | 0.05 | 21.04 |
| 9 | 0.87 | -0.62 | 0.22 | -0.47 | 0.62 | 0.92 | -0.62 | 0.62 | 1.00 | 0.74 | 0.91 | 0.64 | 0.64 | 0.33 | -0.28 | 0.66 | 0.36 | 0.65 | 0.41 | 14.21 |
| 10 | 0.94 | -0.44 | -0.27 | 0.08 | 0.97 | 0.85 | -0.11 | 0.91 | 0.74 | 1.00 | 0.83 | 0.50 | 0.48 | 0.22 | -0.38 | 0.50 | 0.25 | 0.54 | 0.23 | 22.56 |
| 11 | 0.97 | -0.57 | 0.25 | -0.49 | 0.71 | 0.98 | -0.59 | 0.83 | 0.91 | 0.83 | 1.00 | 0.64 | 0.71 | 0.22 | -0.19 | 0.73 | 0.26 | 0.73 | 0.27 | 13.03 |
| 12 | 0.60 | -0.70 | 0.05 | -0.37 | 0.38 | 0.70 | -0.57 | 0.42 | 0.64 | 0.50 | 0.64 | 1.00 | 0.90 | 0.76 | -0.50 | 0.91 | 0.78 | 0.90 | 0.78 | 906.68 |
| 13 | 0.63 | -0.56 | 0.28 | -0.53 | 0.35 | 0.70 | -0.61 | 0.48 | 0.64 | 0.48 | 0.71 | 0.90 | 1.00 | 0.43 | -0.17 | 1.00 | 0.46 | 0.96 | 0.47 | 130.45 |
| 14 | 0.22 | -0.59 | -0.23 | -0.05 | 0.14 | 0.33 | -0.31 | 0.06 | 0.33 | 0.22 | 0.22 | 0.76 | 0.43 | 1.00 | -0.72 | 0.44 | 0.99 | 0.49 | 0.97 | 31.03 |
| 15 | -0.26 | 0.55 | 0.50 | -0.27 | -0.37 | -0.33 | 0.05 | -0.14 | -0.28 | -0.38 | -0.19 | -0.50 | -0.17 | -0.72 | 1.00 | -0.19 | -0.72 | -0.23 | -0.71 | 55.10 |
| 16 | 0.65 | -0.58 | 0.27 | -0.53 | 0.37 | 0.72 | -0.62 | 0.50 | 0.66 | 0.50 | 0.73 | 0.91 | 1.00 | 0.44 | -0.19 | 1.00 | 0.47 | 0.96 | 0.48 | 353.34 |
| 17 | 0.25 | -0.60 | -0.20 | -0.08 | 0.16 | 0.37 | -0.34 | 0.09 | 0.36 | 0.25 | 0.26 | 0.78 | 0.46 | 0.99 | -0.72 | 0.47 | 1.00 | 0.52 | 0.98 | 108.80 |
| 18 | 0.67 | -0.59 | 0.20 | -0.46 | 0.41 | 0.73 | -0.58 | 0.56 | 0.65 | 0.54 | 0.73 | 0.90 | 0.96 | 0.49 | -0.23 | 0.96 | 0.52 | 1.00 | 0.52 | 316.69 |
| 19 | 0.25 | -0.64 | -0.16 | -0.12 | 0.13 | 0.38 | -0.40 | 0.05 | 0.41 | 0.23 | 0.27 | 0.78 | 0.47 | 0.97 | -0.71 | 0.48 | 0.98 | 0.52 | 1.00 | 121.48 |

Table 3.
