# Supplementary material for: Identifying areas and centers of endemism in the Gran Chaco with Fabaceae as a diversity indicator
Source: Sci Rep. 2025 Mar 20;15:9572. doi: 10.1038/s41598-025-90091-3 (PMC11926246; doi:10.1038/s41598-025-90091-3)
Supplement: Supplementary file 6 — Supplementary Material 6 [file 41598_2025_90091_MOESM6_ESM.docx]

**Table 3. Indicator sof performance of the MAXENT models developed to the endemic species of the Gran Chaco ecoregion**

| **Endemism area** | **Species** | **Feature** | **Regularization multiplier** | **AUC Train** | **CBI Train** | **AICc** | **delta AICc** | **auc,diff,avg** | **auc,diff,sd** | **auc,val,avg** | **auc,val,sd** | **cbi,val,avg** | **cbi,val,sd** | **or,10p,avg** | **or,10p,sd** | **or,mtp,avg** | **or,mtp,sd** | **AICc** | **delta AICc** | **w,AIC** | **ncoef** | **tune,args** |
| --- | --- | --- | --- | --- | --- | --- | --- | --- | --- | --- | --- | --- | --- | --- | --- | --- | --- | --- | --- | --- | --- | --- |
| Dry-Sierra Chaco ecotone | Adesmia cordobensis | LQ | 1 | 0,97 | 0,90 | 1068,02 | 0,00 | 0,07 | 0,07 | 0,94 | 0,09 | 0,40 | 0,70 | 0,27 | 0,49 | 0,18 | 0,35 | 1068,02 | 0,00 | 1,00 | 10 | fc,LQ_rm,1 |
| Dry-Sierra Chaco ecotone | Crotalaria chaco-serranensis | LQ | 1 | 0,97 | 0,87 | 1041,98 | 0,00 | 0,02 | 0,01 | 0,97 | 0,02 | 0,54 | 0,18 | 0,08 | 0,11 | 0,06 | 0,11 | 1041,98 | 0,00 | 0,97 | 8 | fc,LQ_rm,1 |
| Dry-Sierra Chaco ecotone | Prosopis flexuosa | LQ | 1 | 0,97 | 0,88 | 813,60 | 0,00 | 0,04 | 0,03 | 0,93 | 0,05 | 0,28 | 0,57 | 0,21 | 0,43 | 0,18 | 0,36 | 813,60 | 0,00 | 1,00 | 7 | fc,LQ_rm,1 |
| Dry-Sierra Chaco ecotone | Prosopis pugionata | LQ | 1 | 0,96 | 0,88 | 566,26 | 0,00 | 0,05 | 0,02 | 0,92 | 0,07 | 0,70 | 0,07 | 0,10 | 0,12 | 0,00 | 0,00 | 566,26 | 0,00 | 0,99 | 7 | fc,LQ_rm,1 |
| Dry-Sierra Chaco ecotone | Senna subulata | LQ | 1 | 0,96 | 0,93 | 555,98 | 0,00 | 0,03 | 0,03 | 0,94 | 0,02 | 0,34 | 0,45 | 0,05 | 0,10 | 0,05 | 0,10 | 555,98 | 0,00 | 0,88 | 8 | fc,LQ_rm,1 |
| Interior Dry Chaco | Arachis batizocoi | LQ | 2 | 0,97 | 0,18 | 255,96 | 0,00 | 0,02 | 0,01 | 0,97 | 0,01 | 0,45 | 0,10 | 0,21 | 0,25 | 0,21 | 0,25 | 255,96 | 0,00 | 0,63 | 4 | fc,LQ_rm,2 |
| Interior Dry Chaco | Chaetocalyx chacoensis | LQ | 2 | 0,99 | 0,91 | 169,71 | 0,00 | 0,02 | 0,03 | 0,98 | 0,02 | 0,35 | 0,67 | 0,38 | 0,48 | 0,13 | 0,25 | 169,71 | 0,00 | 0,77 | 3 | fc,LQ_rm,2 |
| Interior Dry Chaco | Lophocarpinia aculeatifolia | LQ | 1 | 0,98 | 0,65 | 685,47 | 0,50 | 0,02 | 0,01 | 0,98 | 0,02 | 0,54 | 0,12 | 0,16 | 0,24 | 0,04 | 0,08 | 685,47 | 0,50 | 0,34 | 5 | fc,LQ_rm,1 |
| Interior Dry Chaco | Mimosa castanoclada | LQ | 2 | 0,98 | 0,69 | 690,38 | 0,00 | 0,02 | 0,02 | 0,98 | 0,01 | 0,71 | 0,19 | 0,18 | 0,27 | 0,18 | 0,27 | 690,38 | 0,00 | 0,37 | 3 | fc,LQ_rm,2 |
| Interior Dry Chaco | Piptadeniopsis lomentifera | L | 1 | 0,96 | 0,74 | 382,72 | 0,00 | 0,07 | 0,08 | 0,92 | 0,07 | 0,72 | 0,13 | 0,27 | 0,36 | 0,13 | 0,25 | 382,72 | 0,00 | 0,85 | 4 | fc,L_rm,1 |
| Interior Dry Chaco | Prosopis nuda | L | 1 | 0,97 | -0,13 | 246,59 | 0,00 | 0,05 | 0,04 | 0,94 | 0,05 | 0,07 | 0,38 | 0,38 | 0,48 | 0,38 | 0,48 | 246,59 | 0,00 | 0,42 | 3 | fc,L_rm,1 |
| Interior Dry Chaco | Senegalia emilioana | LQH | 2 | 0,98 | 0,64 | 553,31 | 0,00 | 0,04 | 0,02 | 0,97 | 0,02 | 0,74 | 0,13 | 0,25 | 0,38 | 0,15 | 0,19 | 553,31 | 0,00 | 0,83 | 7 | fc,LQH_rm,2 |
| Lower Paraguay River Basin | Arachis lignosa | L | 3 | 0,95 | -0,07 | 206,00 | 0,00 | 0,02 | 0,00 | 0,94 | 0,02 | 0,22 | 0,51 | 0,25 | 0,50 | 0,25 | 0,50 | 206,00 | 0,00 | 0,85 | 3 | fc,L_rm,3 |
| Lower Paraguay River Basin | Bauhinia hagenbeckii | LQ | 2 | 0,94 | 0,25 | 380,78 | 0,00 | 0,04 | 0,02 | 0,93 | 0,04 | 0,39 | 0,34 | 0,13 | 0,25 | 0,06 | 0,13 | 380,78 | 0,00 | 0,91 | 4 | fc,LQ_rm,2 |
| Lower Paraguay River Basin | Prosopis rubiflora | LQH | 2 | 1,00 | 0,78 | 384,19 | 0,00 | 0,01 | 0,01 | 0,99 | 0,01 | 0,27 | 0,79 | 0,13 | 0,25 | 0,13 | 0,25 | 384,19 | 0,00 | 1,00 | 8 | fc,LQH_rm,2 |
| Sierra Chaco | Apurimacia dolichocarpa | L | 1 | 0,91 | 0,61 | 307,87 | 0,00 | 0,02 | 0,01 | 0,85 | 0,06 | 0,54 | 0,19 | 0,42 | 0,29 | 0,21 | 0,25 | 307,87 | 0,00 | 0,92 | 3 | fc,L_rm,1 |
| Sierra Chaco | Dalea elegans | LQ | 1 | 0,99 | 0,93 | 823,45 | 0,00 | 0,02 | 0,01 | 0,97 | 0,03 | 0,71 | 0,08 | 0,06 | 0,07 | 0,03 | 0,06 | 823,45 | 0,00 | 1,00 | 10 | fc,LQ_rm,1 |
| Sierra Chaco | Galactia glaucophylla | LQ | 1 | 0,99 | 0,82 | 806,63 | 0,00 | 0,01 | 0,01 | 0,99 | 0,01 | 0,83 | 0,02 | 0,09 | 0,19 | 0,09 | 0,19 | 806,63 | 0,00 | 1,00 | 9 | fc,LQ_rm,1 |
| Sierra Chaco | Indigofera kurtzii | L | 2 | 0,89 | 0,54 | 185,69 | 0,00 | 0,05 | 0,01 | 0,86 | 0,03 | 0,28 | 0,65 | 0,50 | 0,41 | 0,13 | 0,25 | 185,69 | 0,00 | 0,57 | 2 | fc,L_rm,2 |
| Sierra Chaco | Mimosa cordobensis | L | 2 | 0,81 | -0,23 | 161,92 | 0,00 | 0,05 | 0,04 | 0,79 | 0,06 | 0,26 | 0,58 | 0,25 | 0,50 | 0,25 | 0,50 | 161,92 | 0,00 | 0,54 | 2 | fc,L_rm,2 |
| Upper Paraguay and Paraná Rivers Basin | Arachis correntina | LQ | 1 | 0,99 | 0,63 | 659,91 | 0,00 | 0,01 | 0,01 | 0,98 | 0,01 | 0,23 | 0,53 | 0,21 | 0,43 | 0,18 | 0,36 | 659,91 | 0,00 | 0,78 | 6 | fc,LQ_rm,1 |
| Upper Paraguay and Paraná Rivers Basin | Galactia longifolia | L | 1 | 0,95 | 0,65 | 267,67 | 0,00 | 0,04 | 0,03 | 0,94 | 0,03 | NA | NA | 0,25 | 0,50 | 0,13 | 0,25 | 267,67 | 0,00 | 0,93 | 4 | fc,L_rm,1 |
| Upper Paraguay and Paraná Rivers Basin | Mimosa pseudopetiolaris | LQ | 3 | 0,98 | 0,68 | 209,77 | 0,00 | 0,01 | 0,01 | 0,97 | 0,01 | NA | NA | 0,25 | 0,50 | 0,25 | 0,50 | 209,77 | 0,00 | 1,00 | 4 | fc,LQ_rm,3 |
