# Supplementary material for: Identifying areas and centers of endemism in the Gran Chaco with Fabaceae as a diversity indicator
Source: Sci Rep. 2025 Mar 20;15:9572. doi: 10.1038/s41598-025-90091-3 (PMC11926246; doi:10.1038/s41598-025-90091-3)
Supplement: Supplementary file 7 — Supplementary Material 7 [file 41598_2025_90091_MOESM7_ESM.docx]

**Table 4. Niche expansion, stability and unfilling of endemic taxa of Chacoan Fabaceae.**

| **Areas of endemism** | **expansion** | **stability** | **unfilling** | **species 1** | **species 2** |
| --- | --- | --- | --- | --- | --- |
| Dry Chaco-Dry Chaco | 0.53 | 0.47 | 0.36 | Senegalia emilioana | Arachis batizocoi |
| Dry Chaco-Dry Chaco | 0.36 | 0.64 | 0.53 | Arachis batizocoi | Senegalia emilioana |
| Dry Chaco-Dry Chaco | 0.34 | 0.66 | 0.45 | Senegalia emilioana | Lophocarpinia aculeatifolia |
| Dry Chaco-Dry Chaco | 0.45 | 0.55 | 0.34 | Lophocarpinia aculeatifolia | Senegalia emilioana |
| Dry Chaco-Dry Chaco | 0.06 | 0.94 | 0.14 | Senegalia emilioana | Mimosa castanoclada |
| Dry Chaco-Dry Chaco | 0.14 | 0.86 | 0.06 | Mimosa castanoclada | Senegalia emilioana |
| Dry Chaco-Dry Chaco | 0.06 | 0.94 | 0.30 | Senegalia emilioana | Piptadeniopsis lomentifera |
| Dry Chaco-Dry Chaco | 0.30 | 0.70 | 0.06 | Piptadeniopsis lomentifera | Senegalia emilioana |
| Dry Chaco-Dry Chaco | 0.13 | 0.87 | 0.30 | Senegalia emilioana | Neltuma nuda |
| Dry Chaco-Dry Chaco | 0.30 | 0.70 | 0.13 | Neltuma nuda | Senegalia emilioana |
| Dry Chaco-Dry Chaco | 0.69 | 0.31 | 0.88 | Arachis batizocoi | Lophocarpinia aculeatifolia |
| Dry Chaco-Dry Chaco | 0.88 | 0.12 | 0.69 | Lophocarpinia aculeatifolia | Arachis batizocoi |
| Dry Chaco-Dry Chaco | 0.25 | 0.75 | 0.59 | Arachis batizocoi | Mimosa castanoclada |
| Dry Chaco-Dry Chaco | 0.59 | 0.41 | 0.25 | Mimosa castanoclada | Arachis batizocoi |
| Dry Chaco-Dry Chaco | 0.60 | 0.40 | 0.78 | Arachis batizocoi | Piptadeniopsis lomentifera |
| Dry Chaco-Dry Chaco | 0.78 | 0.22 | 0.60 | Piptadeniopsis lomentifera | Arachis batizocoi |
| Dry Chaco-Dry Chaco | 0.41 | 0.59 | 0.76 | Arachis batizocoi | Neltuma nuda |
| Dry Chaco-Dry Chaco | 0.76 | 0.24 | 0.41 | Neltuma nuda | Arachis batizocoi |
| Dry Chaco-Dry Chaco | 0.51 | 0.49 | 0.37 | Lophocarpinia aculeatifolia | Mimosa castanoclada |
| Dry Chaco-Dry Chaco | 0.37 | 0.63 | 0.51 | Mimosa castanoclada | Lophocarpinia aculeatifolia |
| Dry Chaco-Dry Chaco | 0.17 | 0.83 | 0.34 | Lophocarpinia aculeatifolia | Piptadeniopsis lomentifera |
| Dry Chaco-Dry Chaco | 0.34 | 0.66 | 0.17 | Piptadeniopsis lomentifera | Lophocarpinia aculeatifolia |
| Dry Chaco-Dry Chaco | 0.08 | 0.92 | 0.21 | Lophocarpinia aculeatifolia | Neltuma nuda |
| Dry Chaco-Dry Chaco | 0.21 | 0.79 | 0.08 | Neltuma nuda | Lophocarpinia aculeatifolia |
| Dry Chaco-Dry Chaco | 0.23 | 0.78 | 0.41 | Mimosa castanoclada | Piptadeniopsis lomentifera |
| Dry Chaco-Dry Chaco | 0.41 | 0.59 | 0.23 | Piptadeniopsis lomentifera | Mimosa castanoclada |
| Dry Chaco-Dry Chaco | 0.08 | 0.92 | 0.21 | Mimosa castanoclada | Neltuma nuda |
| Dry Chaco-Dry Chaco | 0.21 | 0.79 | 0.08 | Neltuma nuda | Mimosa castanoclada |
| Dry Chaco-Dry Chaco | 0.30 | 0.70 | 0.22 | Piptadeniopsis lomentifera | Neltuma nuda |
| Dry Chaco-Dry Chaco | 0.22 | 0.78 | 0.30 | Neltuma nuda | Piptadeniopsis lomentifera |
| Dry Chaco-Dry Chaco | 0.63 | 0.37 | 0.00 | Chaetocalyx chacoensis | Senegalia emilioana |
| Dry Chaco-Dry Chaco | 0.00 | 1.00 | 0.63 | Senegalia emilioana | Chaetocalyx chacoensis |
| Dry Chaco-Dry Chaco | 0.78 | 0.22 | 0.04 | Chaetocalyx chacoensis | Arachis batizocoi |
| Dry Chaco-Dry Chaco | 0.04 | 0.96 | 0.78 | Arachis batizocoi | Chaetocalyx chacoensis |
| Dry Chaco-Dry Chaco | 0.83 | 0.17 | 0.59 | Chaetocalyx chacoensis | Lophocarpinia aculeatifolia |
| Dry Chaco-Dry Chaco | 0.59 | 0.41 | 0.83 | Lophocarpinia aculeatifolia | Chaetocalyx chacoensis |
| Dry Chaco-Dry Chaco | 0.52 | 0.48 | 0.01 | Chaetocalyx chacoensis | Mimosa castanoclada |
| Dry Chaco-Dry Chaco | 0.01 | 0.99 | 0.52 | Mimosa castanoclada | Chaetocalyx chacoensis |
| Dry Chaco-Dry Chaco | 0.76 | 0.24 | 0.16 | Chaetocalyx chacoensis | Piptadeniopsis lomentifera |
| Dry Chaco-Dry Chaco | 0.16 | 0.84 | 0.76 | Piptadeniopsis lomentifera | Chaetocalyx chacoensis |
| Dry Chaco-Dry Chaco | 0.65 | 0.35 | 0.27 | Chaetocalyx chacoensis | Neltuma nuda |
| Dry Chaco-Dry Chaco | 0.27 | 0.73 | 0.65 | Neltuma nuda | Chaetocalyx chacoensis |
| Dry Chaco-Dry/Sierra Chaco ecotone | 0.98 | 0.02 | 0.96 | Senegalia emilioana | Senna subulata |
| Dry Chaco-Dry/Sierra Chaco ecotone | 0.96 | 0.04 | 0.98 | Senna subulata | Senegalia emilioana |
| Dry Chaco-Dry/Sierra Chaco ecotone | 1.00 | 0.00 | 1.00 | Senegalia emilioana | Adesmia cordobensis |
| Dry Chaco-Dry/Sierra Chaco ecotone | 1.00 | 0.00 | 1.00 | Adesmia cordobensis | Senegalia emilioana |
| Dry Chaco-Dry/Sierra Chaco ecotone | 1.00 | 0.00 | 1.00 | Senegalia emilioana | Neltuma flexuosa |
| Dry Chaco-Dry/Sierra Chaco ecotone | 1.00 | 0.00 | 1.00 | Neltuma flexuosa | Senegalia emilioana |
| Dry Chaco-Dry/Sierra Chaco ecotone | 1.00 | 0.00 | 1.00 | Senegalia emilioana | Neltuma pugionata |
| Dry Chaco-Dry/Sierra Chaco ecotone | 1.00 | 0.00 | 1.00 | Neltuma pugionata | Senegalia emilioana |
| Dry Chaco-Dry/Sierra Chaco ecotone | 0.88 | 0.16 | 0.86 | Arachis batizocoi | Senna subulata |
| Dry Chaco-Dry/Sierra Chaco ecotone | 0.86 | 0.14 | 0.88 | Senna subulata | Arachis batizocoi |
| Dry Chaco-Dry/Sierra Chaco ecotone | 1.00 | 0.00 | 1.00 | Arachis batizocoi | Neltuma flexuosa |
| Dry Chaco-Dry/Sierra Chaco ecotone | 1.00 | 0.00 | 1.00 | Neltuma flexuosa | Arachis batizocoi |
| Dry Chaco-Dry/Sierra Chaco ecotone | 1.00 | 0.00 | 1.00 | Arachis batizocoi | Neltuma pugionata |
| Dry Chaco-Dry/Sierra Chaco ecotone | 1.00 | 0.00 | 1.00 | Neltuma pugionata | Arachis batizocoi |
| Dry Chaco-Dry/Sierra Chaco ecotone | 1.00 | 0.00 | 1.00 | Lophocarpinia aculeatifolia | Adesmia cordobensis |
| Dry Chaco-Dry/Sierra Chaco ecotone | 1.00 | 0.00 | 1.00 | Adesmia cordobensis | Lophocarpinia aculeatifolia |
| Dry Chaco-Dry/Sierra Chaco ecotone | 1.00 | 0.00 | 1.00 | Lophocarpinia aculeatifolia | Neltuma flexuosa |
| Dry Chaco-Dry/Sierra Chaco ecotone | 1.00 | 0.00 | 1.00 | Neltuma flexuosa | Lophocarpinia aculeatifolia |
| Dry Chaco-Dry/Sierra Chaco ecotone | 1.00 | 0.00 | 1.00 | Lophocarpinia aculeatifolia | Neltuma flexuosa |
| Dry Chaco-Dry/Sierra Chaco ecotone | 1.00 | 0.00 | 1.00 | Neltuma flexuosa | Lophocarpinia aculeatifolia |
| Dry Chaco-Dry/Sierra Chaco ecotone | 0.98 | 0.02 | 0.96 | Mimosa castanoclada | Senna subulata |
| Dry Chaco-Dry/Sierra Chaco ecotone | 0.96 | 0.04 | 0.98 | Senna subulata | Mimosa castanoclada |
| Dry Chaco-Dry/Sierra Chaco ecotone | 1.00 | 0.00 | 1.00 | Mimosa castanoclada | Adesmia cordobensis |
| Dry Chaco-Dry/Sierra Chaco ecotone | 1.00 | 0.00 | 1.00 | Adesmia cordobensis | Mimosa castanoclada |
| Dry Chaco-Dry/Sierra Chaco ecotone | 1.00 | 0.00 | 1.00 | Mimosa castanoclada | Neltuma flexuosa |
| Dry Chaco-Dry/Sierra Chaco ecotone | 1.00 | 0.00 | 1.00 | Neltuma flexuosa | Mimosa castanoclada |
| Dry Chaco-Dry/Sierra Chaco ecotone | 1.00 | 0.00 | 1.00 | Mimosa castanoclada | Neltuma pugionata |
| Dry Chaco-Dry/Sierra Chaco ecotone | 1.00 | 0.00 | 1.00 | Neltuma pugionata | Mimosa castanoclada |
| Dry Chaco-Dry/Sierra Chaco ecotone | 1.00 | 0.00 | 1.00 | Mimosa castanoclada | Neltuma flexuosa |
| Dry Chaco-Dry/Sierra Chaco ecotone | 1.00 | 0.00 | 1.00 | Neltuma flexuosa | Mimosa castanoclada |
| Dry Chaco-Dry/Sierra Chaco ecotone | 1.00 | 0.00 | 1.00 | Mimosa castanoclada | Neltuma pugionata |
| Dry Chaco-Dry/Sierra Chaco ecotone | 1.00 | 0.00 | 1.00 | Neltuma pugionata | Mimosa castanoclada |
| Dry Chaco-Dry/Sierra Chaco ecotone | 1.00 | 0.00 | 1.00 | Piptadeniopsis lomentifera | Senna subulata |
| Dry Chaco-Dry/Sierra Chaco ecotone | 1.00 | 0.00 | 1.00 | Senna subulata | Piptadeniopsis lomentifera |
| Dry Chaco-Dry/Sierra Chaco ecotone | 1.00 | 0.00 | 1.00 | Piptadeniopsis lomentifera | Adesmia cordobensis |
| Dry Chaco-Dry/Sierra Chaco ecotone | 1.00 | 0.00 | 1.00 | Adesmia cordobensis | Piptadeniopsis lomentifera |
| Dry Chaco-Dry/Sierra Chaco ecotone | 1.00 | 0.00 | 1.00 | Piptadeniopsis lomentifera | Neltuma flexuosa |
| Dry Chaco-Dry/Sierra Chaco ecotone | 1.00 | 0.00 | 1.00 | Neltuma flexuosa | Piptadeniopsis lomentifera |
| Dry Chaco-Dry/Sierra Chaco ecotone | 1.00 | 0.00 | 1.00 | Piptadeniopsis lomentifera | Neltuma pugionata |
| Dry Chaco-Dry/Sierra Chaco ecotone | 1.00 | 0.00 | 1.00 | Neltuma pugionata | Piptadeniopsis lomentifera |
| Dry Chaco-Dry/Sierra Chaco ecotone | 0.99 | 0.01 | 0.95 | Neltuma nuda | Senna subulata |
| Dry Chaco-Dry/Sierra Chaco ecotone | 0.95 | 0.05 | 0.99 | Senna subulata | Neltuma nuda |
| Dry Chaco-Dry/Sierra Chaco ecotone | 1.00 | 0.00 | 1.00 | Neltuma nuda | Neltuma flexuosa |
| Dry Chaco-Dry/Sierra Chaco ecotone | 1.00 | 0.00 | 1.00 | Neltuma flexuosa | Neltuma nuda |
| Dry Chaco-Dry/Sierra Chaco ecotone | 1.00 | 0.00 | 1.00 | Neltuma nuda | Neltuma pugionata |
| Dry Chaco-Dry/Sierra Chaco ecotone | 1.00 | 0.00 | 1.00 | Neltuma pugionata | Neltuma nuda |
| Dry Chaco-Dry/Sierra Chaco ecotone | 1.00 | 0.00 | 1.00 | Neltuma nuda | Neltuma flexuosa |
| Dry Chaco-Dry/Sierra Chaco ecotone | 1.00 | 0.00 | 1.00 | Neltuma flexuosa | Neltuma nuda |
| Dry Chaco-Dry/Sierra Chaco ecotone | 1.00 | 0.00 | 1.00 | Neltuma nuda | Neltuma pugionata |
| Dry Chaco-Dry/Sierra Chaco ecotone | 1.00 | 0.00 | 1.00 | Neltuma pugionata | Neltuma nuda |
| Dry Chaco-Upper Paraguay River Basin | 0.93 | 0.07 | 0.99 | Senegalia emilioana | Neltuma rubiflora |
| Dry Chaco-Upper Paraguay River Basin | 0.99 | 0.01 | 0.93 | Neltuma rubiflora | Senegalia emilioana |
| Dry Chaco-Low Paraguay River Basin | 0.93 | 0.07 | 0.99 | Senegalia emilioana | Galactia longifolia |
| Dry Chaco-Low Paraguay River Basin | 0.99 | 0.91 | 0.93 | Galactia longifolia | Senegalia emilioana |
| Dry Chaco-Low Paraguay River Basin | 1.00 | 0.00 | 0.00 | Senegalia emilioana | Arachis correntina |
| Dry Chaco-Low Paraguay River Basin | 0.00 | 1.00 | 1.00 | Arachis correntina | Senegalia emilioana |
| Dry Chaco-Low Paraguay River Basin | 1.00 | 0.00 | 1.00 | Senegalia emilioana | Mimosa pseudopetiolaris |
| Dry Chaco-Low Paraguay River Basin | 1.00 | 0.00 | 1.00 | Mimosa pseudopetiolaris | Senegalia emilioana |
| Dry Chaco-Low Paraguay River Basin | 1.00 | 0.00 | 1.00 | Arachis batizocoi | Arachis correntina |
| Dry Chaco-Low Paraguay River Basin | 1.00 | 0.00 | 1.00 | Arachis correntina | Arachis batizocoi |
| Dry Chaco-Low Paraguay River Basin | 1.00 | 0.00 | 1.00 | Arachis batizocoi | Galactia longifolia |
| Dry Chaco-Low Paraguay River Basin | 1.00 | 0.00 | 1.00 | Galactia longifolia | Arachis batizocoi |
| Dry Chaco-Low Paraguay River Basin | 1.00 | 0.00 | 1.00 | Arachis batizocoi | Mimosa pseudopetiolaris |
| Dry Chaco-Low Paraguay River Basin | 1.00 | 0.00 | 1.00 | Mimosa pseudopetiolaris | Arachis batizocoi |
| Dry Chaco-Low Paraguay River Basin | 0.54 | 0.46 | 0.93 | Lophocarpinia aculeatifolia | Galactia longifolia |
| Dry Chaco-Low Paraguay River Basin | 0.93 | 0.07 | 0.54 | Galactia longifolia | Lophocarpinia aculeatifolia |
| Dry Chaco-Low Paraguay River Basin | 0.55 | 0.46 | 0.93 | Lophocarpinia aculeatifolia | Galactia longifolia |
| Dry Chaco-Low Paraguay River Basin | 0.93 | 0.07 | 0.55 | Galactia longifolia | Lophocarpinia aculeatifolia |
| Dry Chaco-Low Paraguay River Basin | 0.98 | 0.02 | 0.93 | Lophocarpinia aculeatifolia | Neltuma pugionata |
| Dry Chaco-Low Paraguay River Basin | 0.93 | 0.07 | 0.98 | Neltuma pugionata | Lophocarpinia aculeatifolia |
| Dry Chaco-Low Paraguay River Basin | 1.00 | 0.00 | 0.98 | Lophocarpinia aculeatifolia | Senna subulata |
| Dry Chaco-Low Paraguay River Basin | 0.98 | 0.02 | 1.00 | Senna subulata | Lophocarpinia aculeatifolia |
| Dry Chaco-Low Paraguay River Basin | 1.00 | 0.00 | 1.00 | Lophocarpinia aculeatifolia | Mimosa pseudopetiolaris |
| Dry Chaco-Low Paraguay River Basin | 1.00 | 0.00 | 1.00 | Mimosa pseudopetiolaris | Lophocarpinia aculeatifolia |
| Dry Chaco-Low Paraguay River Basin | 1.00 | 0.00 | 1.00 | Lophocarpinia aculeatifolia | Arachis correntina |
| Dry Chaco-Low Paraguay River Basin | 1.00 | 0.00 | 1.00 | Arachis correntina | Lophocarpinia aculeatifolia |
| Dry Chaco-Low Paraguay River Basin | 1.00 | 0.00 | 1.00 | Lophocarpinia aculeatifolia | Mimosa pseudopetiolaris |
| Dry Chaco-Low Paraguay River Basin | 1.00 | 0.00 | 1.00 | Mimosa pseudopetiolaris | Lophocarpinia aculeatifolia |
| Dry Chaco-Low Paraguay River Basin | 0.96 | 0.04 | 0.99 | Mimosa castanoclada | Galactia longifolia |
| Dry Chaco-Low Paraguay River Basin | 0.99 | 0.01 | 0.96 | Galactia longifolia | Mimosa castanoclada |
| Dry Chaco-Low Paraguay River Basin | 0.00 | 0.00 | 0.00 | Mimosa castanoclada | Mimosa pseudopetiolaris |
| Dry Chaco-Low Paraguay River Basin | 0.00 | 0.00 | 0.00 | Mimosa pseudopetiolaris | Mimosa castanoclada |
| Dry Chaco-Low Paraguay River Basin | 1.00 | 0.00 | 1.00 | Mimosa castanoclada | Arachis correntina |
| Dry Chaco-Low Paraguay River Basin | 1.00 | 0.00 | 1.00 | Arachis correntina | Mimosa castanoclada |
| Dry Chaco-Low Paraguay River Basin | 1.00 | 0.00 | 1.00 | Mimosa castanoclada | Mimosa pseudopetiolaris |
| Dry Chaco-Low Paraguay River Basin | 1.00 | 0.00 | 1.00 | Mimosa pseudopetiolaris | Mimosa castanoclada |
| Dry Chaco-Low Paraguay River Basin | 0.97 | 0.29 | 0.98 | Piptadeniopsis lomentifera | Mimosa pseudopetiolaris |
| Dry Chaco-Low Paraguay River Basin | 0.98 | 0.02 | 0.98 | Mimosa pseudopetiolaris | Piptadeniopsis lomentifera |
| Dry Chaco-Low Paraguay River Basin | 0.79 | 0.21 | 0.94 | Piptadeniopsis lomentifera | Galactia longifolia |
| Dry Chaco-Low Paraguay River Basin | 0.94 | 0.06 | 0.79 | Galactia longifolia | Piptadeniopsis lomentifera |
| Dry Chaco-Low Paraguay River Basin | 0.99 | 0.06 | 0.99 | Piptadeniopsis lomentifera | Arachis correntina |
| Dry Chaco-Low Paraguay River Basin | 0.99 | 0.10 |  | Arachis correntina | Piptadeniopsis lomentifera |
| Dry Chaco-Low Paraguay River Basin | 0.97 | 0.03 | 1.00 | Piptadeniopsis lomentifera | Mimosa pseudopetiolaris |
| Dry Chaco-Low Paraguay River Basin | 1.00 | 0.00 | 0.97 | Mimosa pseudopetiolaris | Piptadeniopsis lomentifera |
| Dry Chaco-Low Paraguay River Basin | 1.00 | 0.00 | 1.00 | Neltuma flexuosa | Arachis correntina |
| Dry Chaco-Low Paraguay River Basin | 1.00 | 0.00 | 1.00 | Arachis correntina | Neltuma flexuosa |
| Dry Chaco-Low Paraguay River Basin | 1.00 | 0.00 | 1.00 | Neltuma flexuosa | Galactia longifolia |
| Dry Chaco-Low Paraguay River Basin | 1.00 | 0.00 | 1.00 | Galactia longifolia | Neltuma flexuosa |
| Dry Chaco-Low Paraguay River Basin | 0.00 | 0.00 | 1.00 | Neltuma flexuosa | Mimosa pseudopetiolaris |
| Dry Chaco-Low Paraguay River Basin | 0.00 | 0.00 | 1.00 | Mimosa pseudopetiolaris | Neltuma flexuosa |
| Dry Chaco-Low Paraguay River Basin | 0.93 | 0.08 | 0.98 | Neltuma nuda | Galactia longifolia |
| Dry Chaco-Low Paraguay River Basin | 0.98 | 0.02 | 0.93 | Galactia longifolia | Neltuma nuda |
| Dry Chaco-Low Paraguay River Basin | 0.92 | 0.07 | 0.98 | Neltuma nuda | Galactia longifolia |
| Dry Chaco-Low Paraguay River Basin | 0.98 | 0.02 | 0.92 | Galactia longifolia | Neltuma nuda |
| Dry Chaco-Low Paraguay River Basin | 1.00 | 0.00 | 1.00 | Neltuma nuda | Mimosa pseudopetiolaris |
| Dry Chaco-Low Paraguay River Basin | 1.00 | 0.00 | 1.00 | Mimosa pseudopetiolaris | Neltuma nuda |
| Dry Chaco-Low Paraguay River Basin | 1.00 | 0.00 | 1.00 | Neltuma nuda | Arachis correntina |
| Dry Chaco-Low Paraguay River Basin | 1.00 | 0.00 | 1.00 | Arachis correntina | Neltuma nuda |
| Dry Chaco-Low Paraguay River Basin | 1.00 | 0.00 | 1.00 | Neltuma nuda | Mimosa pseudopetiolaris |
| Dry Chaco-Low Paraguay River Basin | 1.00 | 0.00 | 1.00 | Mimosa pseudopetiolaris | Neltuma nuda |
| Dry Chaco-Sierra Chaco | 1.00 | 0.00 | 1.00 | Senegalia emilioana | Galactia glaucophylla |
| Dry Chaco-Sierra Chaco | 1.00 | 0.00 | 1.00 | Galactia glaucophylla | Senegalia emilioana |
| Dry Chaco-Sierra Chaco | 1.00 | 0.00 | 1.00 | Neltuma nuda | Adesmia cordobensis |
| Dry Chaco-Sierra Chaco | 1.00 | 0.00 | 1.00 | Adesmia cordobensis | Neltuma nuda |
| Dry Sierra Chaco ecotone-Dry Sierra Chaco ecotone | 0.28 | 0.72 | 0.52 | Adesmia cordobensis | Neltuma flexuosa |
| Dry Sierra Chaco ecotone-Dry Sierra Chaco ecotone | 0.52 | 0.48 | 0.28 | Neltuma flexuosa | Adesmia cordobensis |
| Dry Sierra Chaco ecotone-Dry Sierra Chaco ecotone | 0.25 | 0.75 | 0.18 | Adesmia cordobensis | Neltuma pugionata |
| Dry Sierra Chaco ecotone-Dry Sierra Chaco ecotone | 0.18 | 0.82 | 0.25 | Neltuma pugionata | Adesmia cordobensis |
| Dry Sierra Chaco ecotone-Dry Sierra Chaco ecotone | 0.34 | 0.66 | 0.02 | Adesmia cordobensis | Senna subulata |
| Dry Sierra Chaco ecotone-Dry Sierra Chaco ecotone | 0.06 | 0.94 | 1.00 | Senna subulata | Adesmia cordobensis |
| Dry Sierra Chaco ecotone-Dry Sierra Chaco ecotone | 1.00 | 0.00 | 0.06 | Adesmia cordobensis | Senna subulata |
| Dry Sierra Chaco ecotone-Dry Sierra Chaco ecotone | 0.20 | 0.80 | 0.06 | Neltuma flexuosa | Neltuma pugionata |
| Dry Sierra Chaco ecotone-Dry Sierra Chaco ecotone | 0.06 | 0.94 | 0.20 | Neltuma pugionata | Neltuma flexuosa |
| Dry Sierra Chaco ecotone-Dry Sierra Chaco ecotone | 0.50 | 0.50 | 0.04 | Neltuma flexuosa | Senna subulata |
| Dry Sierra Chaco ecotone-Dry Sierra Chaco ecotone | 0.04 | 0.96 | 0.50 | Senna subulata | Neltuma flexuosa |
| Dry Sierra Chaco ecotone-Dry Sierra Chaco ecotone | 0.41 | 0.59 | 0.08 | Neltuma pugionata | Senna subulata |
| Dry Sierra Chaco ecotone-Dry Sierra Chaco ecotone | 0.08 | 0.92 | 0.41 | Senna subulata | Neltuma pugionata |
| Dry Sierra Chaco ecotone-Dry Sierra Chaco ecotone | 0.69 | 0.67 | 0.77 | Crotalaria chaco-serranensis | Neltuma flexuosa |
| Dry Sierra Chaco ecotone-Dry Sierra Chaco ecotone | 0.77 | 0.23 | 0.69 | Neltuma flexuosa | Crotalaria chaco-serranensis |
| Dry Sierra Chaco ecotone-Dry Sierra Chaco ecotone | 0.18 | 0.82 | 0.46 | Crotalaria chaco-serranensis | Neltuma pugionata |
| Dry Sierra Chaco ecotone-Dry Sierra Chaco ecotone | 0.46 | 0.54 | 0.18 | Neltuma pugionata | Crotalaria chaco-serranensis |
| Dry Sierra Chaco ecotone-Dry Sierra Chaco ecotone | 0.10 | 0.90 | 0.45 | Crotalaria chaco-serranensis | Adesmia cordobensis |
| Dry Sierra Chaco ecotone-Dry Sierra Chaco ecotone | 0.45 | 0.55 | 0.10 | Adesmia cordobensis | Crotalaria chaco-serranensis |
| Dry Sierra Chaco ecotone-Dry Sierra Chaco ecotone | 0.19 | 0.81 | 0.17 | Crotalaria chaco-serranensis | Senna subulata |
| Dry Sierra Chaco ecotone-Dry Sierra Chaco ecotone | 0.17 | 0.83 | 0.19 | Senna subulata | Crotalaria chaco-serranensis |
| Dry Sierra Chaco ecotone-Dry Sierra Chaco ecotone | 0.69 | 0.67 | 0.77 | Crotalaria chaco-serranensis | Neltuma flexuosa |
| Dry Sierra Chaco ecotone-Dry Sierra Chaco ecotone | 0.77 | 0.23 | 0.69 | Neltuma flexuosa | Crotalaria chaco-serranensis |
| Dry Sierra Chaco ecotone-Dry Sierra Chaco ecotone | 0.18 | 0.82 | 0.46 | Crotalaria chaco-serranensis | Neltuma pugionata |
| Dry Sierra Chaco ecotone-Dry Sierra Chaco ecotone | 0.46 | 0.54 | 0.18 | Neltuma pugionata | Crotalaria chaco-serranensis |
| Dry Sierra Chaco ecotone-Dry Sierra Chaco ecotone | 0.10 | 0.90 | 0.45 | Crotalaria chaco-serranensis | Adesmia cordobensis |
| Dry Sierra Chaco ecotone-Dry Sierra Chaco ecotone | 0.45 | 0.55 | 0.10 | Adesmia cordobensis | Crotalaria chaco-serranensis |
| Dry Sierra Chaco ecotone-Dry Sierra Chaco ecotone | 0.19 | 0.81 | 0.17 | Crotalaria chaco-serranensis | Senna subulata |
| Dry Sierra Chaco ecotone-Dry Sierra Chaco ecotone | 0.17 | 0.83 | 0.19 | Senna subulata | Crotalaria chaco-serranensis |
| Dry/Sierra Chaco ecotone-Dry Chaco | 0.38 | 0.62 | 0.81 | Crotalaria chaco-serranensis | Senegalia emilioana |
| Dry/Sierra Chaco ecotone-Dry Chaco | 0.81 | 0.19 | 0.38 | Senegalia emilioana | Crotalaria chaco-serranensis |
| Dry/Sierra Chaco ecotone-Dry Chaco | 0.60 | 0.40 | 0.74 | Crotalaria chaco-serranensis | Arachis batizocoi |
| Dry/Sierra Chaco ecotone-Dry Chaco | 0.74 | 0.26 | 0.60 | Arachis batizocoi | Crotalaria chaco-serranensis |
| Dry/Sierra Chaco ecotone-Dry Chaco | 0.31 | 0.69 | 0.87 | Crotalaria chaco-serranensis | Lophocarpinia aculeatifolia |
| Dry/Sierra Chaco ecotone-Dry Chaco | 0.87 | 0.13 | 0.31 | Lophocarpinia aculeatifolia | Crotalaria chaco-serranensis |
| Dry/Sierra Chaco ecotone-Dry Chaco | 0.37 | 0.63 | 0.83 | Crotalaria chaco-serranensis | Mimosa castanoclada |
| Dry/Sierra Chaco ecotone-Dry Chaco | 0.83 | 0.17 | 0.37 | Mimosa castanoclada | Crotalaria chaco-serranensis |
| Dry/Sierra Chaco ecotone-Dry Chaco | 0.43 | 0.57 | 0.90 | Crotalaria chaco-serranensis | Piptadeniopsis lomentifera |
| Dry/Sierra Chaco ecotone-Dry Chaco | 0.90 | 0.10 | 0.43 | Piptadeniopsis lomentifera | Crotalaria chaco-serranensis |
| Dry/Sierra Chaco ecotone-Dry Chaco | 0.35 | 0.65 | 0.85 | Crotalaria chaco-serranensis | Neltuma nuda |
| Dry/Sierra Chaco ecotone-Dry Chaco | 0.85 | 0.15 | 0.35 | Neltuma nuda | Crotalaria chaco-serranensis |
| Dry/Sierra Chaco ecotone-Dry/Sierra Chaco ecotone | 0.28 | 0.72 | 0.52 | Adesmia cordobensis | Neltuma flexuosa |
| Dry/Sierra Chaco ecotone-Dry/Sierra Chaco ecotone | 0.52 | 0.48 | 0.28 | Neltuma flexuosa | Adesmia cordobensis |
| Dry/Sierra Chaco ecotone-Upper Paraguay River Basin | 1.00 | 0.00 | 1.00 | Crotalaria chaco-serranensis | Arachis lignosa |
| Dry/Sierra Chaco ecotone-Upper Paraguay River Basin | 1.00 | 0.00 | 1.00 | Arachis lignosa | Crotalaria chaco-serranensis |
| Dry/Sierra Chaco ecotone-Upper Paraguay River Basin | 1.00 | 0.00 | 1.00 | Crotalaria chaco-serranensis | Bauhinia hagenbeckii |
| Dry/Sierra Chaco ecotone-Upper Paraguay River Basin | 1.00 | 0.00 | 1.00 | Bauhinia hagenbeckii | Crotalaria chaco-serranensis |
| Dry/Sierra Chaco ecotone-Upper Paraguay River Basin | 1.00 | 0.00 | 1.00 | Crotalaria chaco-serranensis | Neltuma rubiflora |
| Dry/Sierra Chaco ecotone-Upper Paraguay River Basin | 1.00 | 0.00 | 1.00 | Neltuma rubiflora | Crotalaria chaco-serranensis |
| Dry/Sierra Chaco ecotone-Low Paraguay River Basin | 1.00 | 0.00 | 1.00 | Adesmia cordobensis | Galactia longifolia |
| Dry/Sierra Chaco ecotone-Low Paraguay River Basin | 1.00 | 0.00 | 1.00 | Galactia longifolia | Adesmia cordobensis |
| Dry/Sierra Chaco ecotone-Low Paraguay River Basin | 0.00 | 0.00 | 0.00 | Adesmia cordobensis | Mimosa pseudopetiolaris |
| Dry/Sierra Chaco ecotone-Low Paraguay River Basin | 0.00 | 0.00 | 0.00 | Mimosa pseudopetiolaris | Adesmia cordobensis |
| Dry/Sierra Chaco ecotone-Low Paraguay River Basin | 1.00 | 0.00 | 1.00 | Adesmia cordobensis | Arachis correntina |
| Dry/Sierra Chaco ecotone-Low Paraguay River Basin | 1.00 | 0.00 | 1.00 | Arachis correntina | Adesmia cordobensis |
| Dry/Sierra Chaco ecotone-Low Paraguay River Basin | 1.00 | 0.00 | 1.00 | Adesmia cordobensis | Galactia longifolia |
| Dry/Sierra Chaco ecotone-Low Paraguay River Basin | 1.00 | 0.00 | 1.00 | Galactia longifolia | Adesmia cordobensis |
| Dry/Sierra Chaco ecotone-Low Paraguay River Basin | 0.00 | 0.00 | 0.00 | Adesmia cordobensis | Mimosa pseudopetiolaris |
| Dry/Sierra Chaco ecotone-Low Paraguay River Basin | 0.00 | 0.00 | 0.00 | Mimosa pseudopetiolaris | Adesmia cordobensis |
| Dry/Sierra Chaco ecotone-Low Paraguay River Basin | 1.00 | 0.00 | 1.00 | Neltuma pugionata | Arachis correntina |
| Dry/Sierra Chaco ecotone-Low Paraguay River Basin | 1.00 | 0.00 | 1.00 | Arachis correntina | Neltuma pugionata |
| Dry/Sierra Chaco ecotone-Low Paraguay River Basin | 1.00 | 0.00 | 1.00 | Neltuma pugionata | Galactia longifolia |
| Dry/Sierra Chaco ecotone-Low Paraguay River Basin | 1.00 | 0.00 | 1.00 | Galactia longifolia | Neltuma pugionata |
| Dry/Sierra Chaco ecotone-Low Paraguay River Basin | 0.00 | 0.00 | 1.00 | Neltuma pugionata | Mimosa pseudopetiolaris |
| Dry/Sierra Chaco ecotone-Low Paraguay River Basin | 0.00 | 0.00 | 1.00 | Mimosa pseudopetiolaris | Neltuma pugionata |
| Dry/Sierra Chaco ecotone-Low Paraguay River Basin | 1.00 | 0.00 | 1.00 | Senna subulata | Arachis correntina |
| Dry/Sierra Chaco ecotone-Low Paraguay River Basin | 1.00 | 0.00 | 1.00 | Arachis correntina | Senna subulata |
| Dry/Sierra Chaco ecotone-Low Paraguay River Basin | 1.00 | 0.00 | 1.00 | Senna subulata | Galactia longifolia |
| Dry/Sierra Chaco ecotone-Low Paraguay River Basin | 1.00 | 0.00 | 1.00 | Galactia longifolia | Senna subulata |
| Dry/Sierra Chaco ecotone-Low Paraguay River Basin | 0.00 | 0.00 | 1.00 | Senna subulata | Mimosa pseudopetiolaris |
| Dry/Sierra Chaco ecotone-Low Paraguay River Basin | 0.00 | 0.00 | 1.00 | Mimosa pseudopetiolaris | Senna subulata |
| Dry/Sierra Chaco ecotone-Low Paraguay River Basin | 1.00 | 0.00 | 1.00 | Crotalaria chaco-serranensis | Arachis correntina |
| Dry/Sierra Chaco ecotone-Low Paraguay River Basin | 1.00 | 0.00 | 1.00 | Arachis correntina | Crotalaria chaco-serranensis |
| Dry/Sierra Chaco ecotone-Low Paraguay River Basin | 1.00 | 0.00 | 1.00 | Crotalaria chaco-serranensis | Galactia longifolia |
| Dry/Sierra Chaco ecotone-Low Paraguay River Basin | 1.00 | 0.00 | 1.00 | Galactia longifolia | Crotalaria chaco-serranensis |
| Dry/Sierra Chaco ecotone-Low Paraguay River Basin | 1.00 | 0.00 | 1.00 | Crotalaria chaco-serranensis | Mimosa pseudopetiolaris |
| Dry/Sierra Chaco ecotone-Low Paraguay River Basin | 1.00 | 0.00 | 1.00 | Mimosa pseudopetiolaris | Crotalaria chaco-serranensis |
| Dry/Sierra Chaco ecotone-Sierra Chaco | 0.22 | 0.78 | 0.30 | Crotalaria chaco-serranensis | Dalea elegans |
| Dry/Sierra Chaco ecotone-Sierra Chaco | 0.30 | 0.70 | 0.22 | Dalea elegans | Crotalaria chaco-serranensis |
| Dry/Sierra Chaco ecotone-Sierra Chaco | 0.00 | 1.00 | 0.57 | Crotalaria chaco-serranensis | Galactia glaucophylla |
| Dry/Sierra Chaco ecotone-Sierra Chaco | 0.57 | 0.43 | 0.00 | Galactia glaucophylla | Crotalaria chaco-serranensis |
| Dry/Sierra Chaco ecotone-Sierra Chaco | 0.00 | 1.00 | 0.76 | Crotalaria chaco-serranensis | Indigofera kurtzii |
| Dry/Sierra Chaco ecotone-Sierra Chaco | 0.76 | 0.24 | 0.00 | Indigofera kurtzii | Crotalaria chaco-serranensis |
| Dry/Sierra Chaco ecotone-Sierra Chaco | 0.00 | 1.00 | 0.75 | Crotalaria chaco-serranensis | Mimosa cordobensis |
| Dry/Sierra Chaco ecotone-Sierra Chaco | 0.75 | 0.25 | 0.00 | Mimosa cordobensis | Crotalaria chaco-serranensis |
| Upper Paraguay River Basin-Dry Chaco | 0.99 | 0.01 | 0.78 | Arachis lignosa | Piptadeniopsis lomentifera |
| Upper Paraguay River Basin-Dry Chaco | 0.78 | 0.22 | 0.99 | Piptadeniopsis lomentifera | Arachis lignosa |
| Upper Paraguay River Basin-Dry Chaco | 1.00 | 0.00 | 0.96 | Arachis lignosa | Lophocarpinia aculeatifolia |
| Upper Paraguay River Basin-Dry Chaco | 0.96 | 0.04 | 1.00 | Lophocarpinia aculeatifolia | Arachis lignosa |
| Upper Paraguay River Basin-Dry Chaco | 1.00 | 0.00 | 0.97 | Arachis lignosa | Senegalia emilioana |
| Upper Paraguay River Basin-Dry Chaco | 0.97 | 0.03 | 1.00 | Senegalia emilioana | Arachis lignosa |
| Upper Paraguay River Basin-Dry Chaco | 1.00 | 0.00 | 0.97 | Arachis lignosa | Mimosa castanoclada |
| Upper Paraguay River Basin-Dry Chaco | 0.97 | 0.03 | 1.00 | Mimosa castanoclada | Arachis lignosa |
| Upper Paraguay River Basin-Dry Chaco | 1.00 | 0.00 | 1.00 | Arachis lignosa | Arachis batizocoi |
| Upper Paraguay River Basin-Dry Chaco | 1.00 | 0.00 | 1.00 | Arachis batizocoi | Arachis lignosa |
| Upper Paraguay River Basin-Dry Chaco | 1.00 | 0.00 | 1.00 | Arachis lignosa | Neltuma nuda |
| Upper Paraguay River Basin-Dry Chaco | 1.00 | 0.00 | 1.00 | Neltuma nuda | Arachis lignosa |
| Upper Paraguay River Basin-Dry Chaco | 0.74 | 0.26 | 0.76 | Bauhinia hagenbeckii | Piptadeniopsis lomentifera |
| Upper Paraguay River Basin-Dry Chaco | 0.76 | 0.24 | 0.74 | Piptadeniopsis lomentifera | Bauhinia hagenbeckii |
| Upper Paraguay River Basin-Dry Chaco | 0.82 | 0.18 | 0.75 | Bauhinia hagenbeckii | Senegalia emilioana |
| Upper Paraguay River Basin-Dry Chaco | 0.75 | 0.25 | 0.82 | Senegalia emilioana | Bauhinia hagenbeckii |
| Upper Paraguay River Basin-Dry Chaco | 0.85 | 0.15 | 0.86 | Bauhinia hagenbeckii | Neltuma nuda |
| Upper Paraguay River Basin-Dry Chaco | 0.86 | 0.14 | 0.85 | Neltuma nuda | Bauhinia hagenbeckii |
| Upper Paraguay River Basin-Dry Chaco | 0.85 | 0.15 | 0.85 | Bauhinia hagenbeckii | Lophocarpinia aculeatifolia |
| Upper Paraguay River Basin-Dry Chaco | 0.85 | 0.15 | 0.85 | Lophocarpinia aculeatifolia | Bauhinia hagenbeckii |
| Upper Paraguay River Basin-Dry Chaco | 0.86 | 0.14 | 0.85 | Bauhinia hagenbeckii | Mimosa castanoclada |
| Upper Paraguay River Basin-Dry Chaco | 0.85 | 0.15 | 0.86 | Mimosa castanoclada | Bauhinia hagenbeckii |
| Upper Paraguay River Basin-Dry Chaco | 0.99 | 0.00 | 0.99 | Bauhinia hagenbeckii | Arachis batizocoi |
| Upper Paraguay River Basin-Dry Chaco | 0.99 | 0.01 | 0.99 | Arachis batizocoi | Bauhinia hagenbeckii |
| Upper Paraguay River Basin-Dry Chaco | 0.99 | 0.01 | 0.86 | Neltuma rubiflora | Piptadeniopsis lomentifera |
| Upper Paraguay River Basin-Dry Chaco | 0.86 | 0.14 | 0.99 | Piptadeniopsis lomentifera | Neltuma rubiflora |
| Upper Paraguay River Basin-Dry Chaco | 0.99 | 0.01 | 0.88 | Neltuma rubiflora | Mimosa castanoclada |
| Upper Paraguay River Basin-Dry Chaco | 0.88 | 0.12 | 0.99 | Mimosa castanoclada | Neltuma rubiflora |
| Upper Paraguay River Basin-Dry Chaco | 0.99 | 0.01 | 0.97 | Neltuma rubiflora | Neltuma nuda |
| Upper Paraguay River Basin-Dry Chaco | 0.97 | 0.03 | 0.99 | Neltuma nuda | Neltuma rubiflora |
| Upper Paraguay River Basin-Dry Chaco | 0.99 | 0.01 | 0.93 | Neltuma rubiflora | Senegalia emilioana |
| Upper Paraguay River Basin-Dry Chaco | 0.93 | 0.07 | 0.99 | Senegalia emilioana | Neltuma rubiflora |
| Upper Paraguay River Basin-Dry Chaco | 1.00 | 0.00 | 0.99 | Neltuma rubiflora | Lophocarpinia aculeatifolia |
| Upper Paraguay River Basin-Dry Chaco | 0.99 | 0.01 | 1.00 | Lophocarpinia aculeatifolia | Neltuma rubiflora |
| Upper Paraguay River Basin-Dry Chaco | 1.00 | 0.00 | 1.00 | Neltuma rubiflora | Arachis batizocoi |
| Upper Paraguay River Basin-Dry Chaco | 1.00 | 0.00 | 1.00 | Arachis batizocoi | Neltuma rubiflora |
| Upper Paraguay River Basin-Dry/Sierra Chaco ecotone | 0.00 | 0.00 | 0.00 | Arachis lignosa | Adesmia cordobensis |
| Upper Paraguay River Basin-Dry/Sierra Chaco ecotone | 0.00 | 0.00 | 0.00 | Adesmia cordobensis | Arachis lignosa |
| Upper Paraguay River Basin-Dry/Sierra Chaco ecotone | 1.00 | 0.00 | 0.00 | Arachis lignosa | Neltuma flexuosa |
| Upper Paraguay River Basin-Dry/Sierra Chaco ecotone | 1.00 | 0.00 | 0.00 | Neltuma flexuosa | Arachis lignosa |
| Upper Paraguay River Basin-Dry/Sierra Chaco ecotone | 1.00 | 0.00 | 0.00 | Arachis lignosa | Neltuma pugionata |
| Upper Paraguay River Basin-Dry/Sierra Chaco ecotone | 1.00 | 0.00 | 0.00 | Neltuma pugionata | Arachis lignosa |
| Upper Paraguay River Basin-Dry/Sierra Chaco ecotone | 1.00 | 0.00 | 1.00 | Arachis lignosa | Senna subulata |
| Upper Paraguay River Basin-Dry/Sierra Chaco ecotone | 1.00 | 0.00 | 1.00 | Senna subulata | Arachis lignosa |
| Upper Paraguay River Basin-Dry/Sierra Chaco ecotone | 1.00 | 0.00 | 1.00 | Bauhinia hagenbeckii | Adesmia cordobensis |
| Upper Paraguay River Basin-Dry/Sierra Chaco ecotone | 1.00 | 0.00 | 1.00 | Adesmia cordobensis | Bauhinia hagenbeckii |
| Upper Paraguay River Basin-Dry/Sierra Chaco ecotone | 1.00 | 0.00 | 1.00 | Bauhinia hagenbeckii | Neltuma flexuosa |
| Upper Paraguay River Basin-Dry/Sierra Chaco ecotone | 1.00 | 0.00 | 1.00 | Neltuma flexuosa | Bauhinia hagenbeckii |
| Upper Paraguay River Basin-Dry/Sierra Chaco ecotone | 1.00 | 0.00 | 1.00 | Bauhinia hagenbeckii | Neltuma pugionata |
| Upper Paraguay River Basin-Dry/Sierra Chaco ecotone | 1.00 | 0.00 | 1.00 | Neltuma pugionata | Bauhinia hagenbeckii |
| Upper Paraguay River Basin-Dry/Sierra Chaco ecotone | 1.00 | 0.00 | 1.00 | Bauhinia hagenbeckii | Senna subulata |
| Upper Paraguay River Basin-Dry/Sierra Chaco ecotone | 0.00 | 0.00 | 1.00 | Neltuma rubiflora | Adesmia cordobensis |
| Upper Paraguay River Basin-Dry/Sierra Chaco ecotone | 0.00 | 0.00 | 1.00 | Adesmia cordobensis | Neltuma rubiflora |
| Upper Paraguay River Basin-Dry/Sierra Chaco ecotone | 0.00 | 0.00 | 0.00 | Neltuma rubiflora | Neltuma flexuosa |
| Upper Paraguay River Basin-Dry/Sierra Chaco ecotone | 0.00 | 0.00 | 0.00 | Neltuma flexuosa | Neltuma rubiflora |
| Upper Paraguay River Basin-Dry/Sierra Chaco ecotone | 1.00 | 0.00 | 1.00 | Neltuma rubiflora | Neltuma pugionata |
| Upper Paraguay River Basin-Dry/Sierra Chaco ecotone | 1.00 | 0.00 | 1.00 | Neltuma pugionata | Neltuma rubiflora |
| Upper Paraguay River Basin-Dry/Sierra Chaco ecotone | 1.00 | 0.00 | 1.00 | Neltuma rubiflora | Senna subulata |
| Upper Paraguay River Basin-Dry/Sierra Chaco ecotone | 1.00 | 0.00 | 1.00 | Senna subulata | Neltuma rubiflora |
| Upper Paraguay River Basin-Upper Paraguay River Basin | 0.08 | 0.81 | 0.19 | Arachis lignosa | Bauhinia hagenbeckii |
| Upper Paraguay River Basin-Upper Paraguay River Basin | 0.19 | 0.81 | 0.08 | Bauhinia hagenbeckii | Arachis lignosa |
| Upper Paraguay River Basin-Upper Paraguay River Basin | 0.21 | 0.79 | 0.36 | Arachis lignosa | Neltuma rubiflora |
| Upper Paraguay River Basin-Upper Paraguay River Basin | 0.36 | 0.64 | 0.21 | Neltuma rubiflora | Arachis lignosa |
| Upper Paraguay River Basin-Upper Paraguay River Basin | 0.01 | 0.99 | 0.82 | Bauhinia hagenbeckii | Neltuma rubiflora |
| Upper Paraguay River Basin-Upper Paraguay River Basin | 0.82 | 0.18 | 0.01 | Neltuma rubiflora | Bauhinia hagenbeckii |
| Upper Paraguay River Basin-Low Paraguay River Basin | 1.00 | 0.00 | 1.00 | Arachis lignosa | Arachis correntina |
| Upper Paraguay River Basin-Low Paraguay River Basin | 1.00 | 0.00 | 1.00 | Arachis correntina | Arachis lignosa |
| Upper Paraguay River Basin-Low Paraguay River Basin | 1.00 | 0.00 | 1.00 | Arachis lignosa | Galactia longifolia |
| Upper Paraguay River Basin-Low Paraguay River Basin | 1.00 | 0.00 | 1.00 | Galactia longifolia | Arachis lignosa |
| Upper Paraguay River Basin-Low Paraguay River Basin | 1.00 | 0.00 | 1.00 | Arachis lignosa | Mimosa pseudopetiolaris |
| Upper Paraguay River Basin-Low Paraguay River Basin | 1.00 | 0.00 | 1.00 | Mimosa pseudopetiolaris | Arachis lignosa |
| Upper Paraguay River Basin-Low Paraguay River Basin | 0.00 | 1.00 | 0.80 | Bauhinia hagenbeckii | Mimosa pseudopetiolaris |
| Upper Paraguay River Basin-Low Paraguay River Basin | 0.00 | 1.00 | 0.80 | Mimosa pseudopetiolaris | Bauhinia hagenbeckii |
| Upper Paraguay River Basin-Low Paraguay River Basin | 0.86 | 0.15 | 0.85 | Bauhinia hagenbeckii | Galactia longifolia |
| Upper Paraguay River Basin-Low Paraguay River Basin | 0.85 | 0.15 | 0.86 | Galactia longifolia | Bauhinia hagenbeckii |
| Upper Paraguay River Basin-Low Paraguay River Basin | 0.91 | 0.09 | 0.83 | Bauhinia hagenbeckii | Arachis correntina |
| Upper Paraguay River Basin-Low Paraguay River Basin | 0.83 | 0.17 | 0.91 | Arachis correntina | Bauhinia hagenbeckii |
| Upper Paraguay River Basin-Low Paraguay River Basin | 1.00 | 0.00 | 1.00 | Neltuma rubiflora | Arachis correntina |
| Upper Paraguay River Basin-Low Paraguay River Basin | 1.00 | 0.00 | 1.00 | Arachis correntina | Neltuma rubiflora |
| Upper Paraguay River Basin-Low Paraguay River Basin | 1.00 | 0.00 | 1.00 | Neltuma rubiflora | Galactia longifolia |
| Upper Paraguay River Basin-Low Paraguay River Basin | 1.00 | 0.00 | 1.00 | Galactia longifolia | Neltuma rubiflora |
| Upper Paraguay River Basin-Low Paraguay River Basin | 1.00 | 0.00 | 1.00 | Neltuma rubiflora | Mimosa pseudopetiolaris |
| Upper Paraguay River Basin-Low Paraguay River Basin | 1.00 | 0.00 | 1.00 | Mimosa pseudopetiolaris | Neltuma rubiflora |
| Low Paraguay River Basin-Upper Paraguay River Basin | 1.00 | 0.00 | 1.00 | Arachis correntina | Neltuma rubiflora |
| Low Paraguay River Basin-Upper Paraguay River Basin | 1.00 | 0.00 | 1.00 | Neltuma rubiflora | Arachis correntina |
| Low Paraguay/Paraná River Basins-Low Paraguay/Paraná River Basins | 0.60 | 0.40 | 0.68 | Arachis correntina | Galactia longifolia |
| Low Paraguay/Paraná River Basins-Low Paraguay/Paraná River Basins | 0.68 | 0.32 | 0.60 | Galactia longifolia | Arachis correntina |
| Low Paraguay/Paraná River Basins-Low Paraguay/Paraná River Basins | 0.14 | 0.86 | 0.92 | Arachis correntina | Mimosa pseudopetiolaris |
| Low Paraguay/Paraná River Basins-Low Paraguay/Paraná River Basins | 0.92 | 0.08 | 0.14 | Mimosa pseudopetiolaris | Arachis correntina |
| Low Paraguay/Paraná River Basins-Low Paraguay/Paraná River Basins | 1.00 | 0.00 | 1.00 | Galactia longifolia | Mimosa pseudopetiolaris |
| Low Paraguay/Paraná River Basins-Low Paraguay/Paraná River Basins | 1.00 | 0.00 | 1.00 | Mimosa pseudopetiolaris | Galactia longifolia |
| Low Paraguay River Basin-Sierra Chaco | 1.00 | 0.00 | 1.00 | Arachis correntina | Galactia glaucophylla |
| Low Paraguay River Basin-Sierra Chaco | 1.00 | 0.00 | 1.00 | Galactia glaucophylla | Arachis correntina |
| Sierra Chaco-Dry Chaco | 0.96 | 0.38 | 0.99 | Dalea elegans | Lophocarpinia aculeatifolia |
| Sierra Chaco-Dry Chaco | 0.99 | 0.01 | 0.96 | Lophocarpinia aculeatifolia | Dalea elegans |
| Sierra Chaco-Dry Chaco | 1.00 | 0.00 | 1.00 | Dalea elegans | Arachis batizocoi |
| Sierra Chaco-Dry Chaco | 1.00 | 0.00 | 1.00 | Arachis batizocoi | Dalea elegans |
| Sierra Chaco-Dry Chaco | 1.00 | 0.00 | 1.00 | Dalea elegans | Senegalia emilioana |
| Sierra Chaco-Dry Chaco | 1.00 | 0.00 | 1.00 | Senegalia emilioana | Dalea elegans |
| Sierra Chaco-Dry Chaco | 1.00 | 0.00 | 1.00 | Dalea elegans | Mimosa castanoclada |
| Sierra Chaco-Dry Chaco | 1.00 | 0.00 | 1.00 | Mimosa castanoclada | Dalea elegans |
| Sierra Chaco-Dry Chaco | 1.00 | 0.00 | 1.00 | Dalea elegans | Piptadeniopsis lomentifera |
| Sierra Chaco-Dry Chaco | 1.00 | 0.00 | 1.00 | Piptadeniopsis lomentifera | Dalea elegans |
| Sierra Chaco-Dry Chaco | 1.00 | 0.00 | 1.00 | Dalea elegans | Neltuma nuda |
| Sierra Chaco-Dry Chaco | 1.00 | 0.00 | 1.00 | Neltuma nuda | Dalea elegans |
| Sierra Chaco-Dry Chaco | 1.00 | 0.00 | 1.00 | Indigofera kurtzii | Senegalia emilioana |
| Sierra Chaco-Dry Chaco | 1.00 | 0.00 | 1.00 | Senegalia emilioana | Indigofera kurtzii |
| Sierra Chaco-Dry Chaco | 1.00 | 0.00 | 1.00 | Indigofera kurtzii | Arachis batizocoi |
| Sierra Chaco-Dry Chaco | 1.00 | 0.00 | 1.00 | Arachis batizocoi | Indigofera kurtzii |
| Sierra Chaco-Dry Chaco | 1.00 | 0.00 | 1.00 | Indigofera kurtzii | Lophocarpinia aculeatifolia |
| Sierra Chaco-Dry Chaco | 1.00 | 0.00 | 1.00 | Lophocarpinia aculeatifolia | Indigofera kurtzii |
| Sierra Chaco-Dry Chaco | 1.00 | 0.00 | 1.00 | Indigofera kurtzii | Mimosa castanoclada |
| Sierra Chaco-Dry Chaco | 1.00 | 0.00 | 1.00 | Mimosa castanoclada | Indigofera kurtzii |
| Sierra Chaco-Dry Chaco | 1.00 | 0.00 | 1.00 | Indigofera kurtzii | Piptadeniopsis lomentifera |
| Sierra Chaco-Dry Chaco | 1.00 | 0.00 | 1.00 | Piptadeniopsis lomentifera | Indigofera kurtzii |
| Sierra Chaco-Dry Chaco | 1.00 | 0.00 | 1.00 | Indigofera kurtzii | Neltuma nuda |
| Sierra Chaco-Dry Chaco | 1.00 | 0.00 | 1.00 | Neltuma nuda | Indigofera kurtzii |
| Sierra Chaco-Dry Chaco | 1.00 | 0.00 | 1.00 | Mimosa cordobensis | Senegalia emilioana |
| Sierra Chaco-Dry Chaco | 1.00 | 0.00 | 1.00 | Senegalia emilioana | Mimosa cordobensis |
| Sierra Chaco-Dry Chaco | 0.00 | 0.00 | 1.00 | Mimosa cordobensis | Arachis batizocoi |
| Sierra Chaco-Dry Chaco | 0.00 | 0.00 | 1.00 | Arachis batizocoi | Mimosa cordobensis |
| Sierra Chaco-Dry Chaco | 1.00 | 0.00 | 1.00 | Mimosa cordobensis | Lophocarpinia aculeatifolia |
| Sierra Chaco-Dry Chaco | 1.00 | 0.00 | 1.00 | Lophocarpinia aculeatifolia | Mimosa cordobensis |
| Sierra Chaco-Dry Chaco | 1.00 | 0.00 | 1.00 | Mimosa cordobensis | Mimosa castanoclada |
| Sierra Chaco-Dry Chaco | 1.00 | 0.00 | 1.00 | Mimosa castanoclada | Mimosa cordobensis |
| Sierra Chaco-Dry Chaco | 1.00 | 0.00 | 1.00 | Mimosa cordobensis | Piptadeniopsis lomentifera |
| Sierra Chaco-Dry Chaco | 1.00 | 0.00 | 1.00 | Piptadeniopsis lomentifera | Mimosa cordobensis |
| Sierra Chaco-Dry Chaco | 1.00 | 0.00 | 1.00 | Mimosa cordobensis | Neltuma nuda |
| Sierra Chaco-Dry Chaco | 1.00 | 0.00 | 1.00 | Neltuma nuda | Mimosa cordobensis |
| Sierra Chaco-Upper Paraguay River Basin | 1.00 | 0.00 | 1.00 | Dalea elegans | Arachis lignosa |
| Sierra Chaco-Upper Paraguay River Basin | 1.00 | 0.00 | 1.00 | Arachis lignosa | Dalea elegans |
| Sierra Chaco-Upper Paraguay River Basin | 1.00 | 0.00 | 1.00 | Dalea elegans | Bauhinia hagenbeckii |
| Sierra Chaco-Upper Paraguay River Basin | 1.00 | 0.00 | 1.00 | Bauhinia hagenbeckii | Dalea elegans |
| Sierra Chaco-Upper Paraguay River Basin | 1.00 | 0.00 | 1.00 | Dalea elegans | Neltuma rubiflora |
| Sierra Chaco-Upper Paraguay River Basin | 1.00 | 0.00 | 1.00 | Neltuma rubiflora | Dalea elegans |
| Sierra Chaco-Upper Paraguay River Basin | 1.00 | 0.00 | 1.00 | Arachis correntina | Dalea elegans |
| Sierra Chaco-Upper Paraguay River Basin | 1.00 | 0.00 | 1.00 | Galactia longifolia | Dalea elegans |
| Sierra Chaco-Upper Paraguay River Basin | 0.00 | 0.00 | 0.00 | Mimosa pseudopetiolaris | Dalea elegans |
| Sierra Chaco-Upper Paraguay River Basin | 0.00 | 0.00 | 0.00 | Neltuma rubiflora | Galactia glaucophylla |
| Sierra Chaco-Upper Paraguay River Basin | 0.00 | 0.00 | 0.00 | Mimosa cordobensis | Arachis lignosa |
| Sierra Chaco-Upper Paraguay River Basin | 0.00 | 0.00 | 0.00 | Arachis lignosa | Mimosa cordobensis |
| Sierra Chaco-Upper Paraguay River Basin | 0.00 | 0.00 | 1.00 | Mimosa cordobensis | Bauhinia hagenbeckii |
| Sierra Chaco-Upper Paraguay River Basin | 0.00 | 0.00 | 1.00 | Bauhinia hagenbeckii | Mimosa cordobensis |
| Sierra Chaco-Upper Paraguay River Basin | 0.00 | 0.00 | 0.00 | Mimosa cordobensis | Neltuma rubiflora |
| Sierra Chaco-Upper Paraguay River Basin | 0.00 | 0.00 | 0.00 | Neltuma rubiflora | Mimosa cordobensis |
| Sierra Chaco-Low Paraguay River Basin | 1.00 | 0.00 | 1.00 | Dalea elegans | Arachis correntina |
| Sierra Chaco-Low Paraguay River Basin | 1.00 | 0.00 | 1.00 | Dalea elegans | Galactia longifolia |
| Sierra Chaco-Low Paraguay River Basin | 0.00 | 0.00 | 0.00 | Dalea elegans | Mimosa pseudopetiolaris |
| Sierra Chaco-Low Paraguay River Basin | 0.00 | 0.00 | 0.00 | Galactia glaucophylla | Neltuma rubiflora |
| Sierra Chaco-Low Paraguay River Basin | 0.00 | 0.00 | 1.00 | Mimosa cordobensis | Arachis correntina |
| Sierra Chaco-Low Paraguay River Basin | 0.00 | 0.00 | 1.00 | Arachis correntina | Mimosa cordobensis |
| Sierra Chaco-Low Paraguay River Basin | 1.00 | 0.00 | 1.00 | Mimosa cordobensis | Galactia longifolia |
| Sierra Chaco-Low Paraguay River Basin | 1.00 | 0.00 | 1.00 | Galactia longifolia | Mimosa cordobensis |
| Sierra Chaco-Low Paraguay River Basin | 1.00 | 0.00 | 1.00 | Mimosa cordobensis | Mimosa pseudopetiolaris |
| Sierra Chaco-Low Paraguay River Basin | 1.00 | 0.00 | 1.00 | Mimosa pseudopetiolaris | Mimosa cordobensis |
| Sierra Chaco-Sierra Chaco | 0.00 | 1.00 | 0.45 | Dalea elegans | Galactia glaucophylla |
| Sierra Chaco-Sierra Chaco | 0.45 | 0.55 | 0.00 | Galactia glaucophylla | Dalea elegans |
| Sierra Chaco-Sierra Chaco | 0.00 | 1.00 | 0.71 | Dalea elegans | Indigofera kurtzii |
| Sierra Chaco-Sierra Chaco | 0.71 | 0.29 | 0.00 | Indigofera kurtzii | Dalea elegans |
| Sierra Chaco-Sierra Chaco | 0.00 | 1.00 | 0.71 | Dalea elegans | Indigofera kurtzii |
| Sierra Chaco-Sierra Chaco | 0.00 | 1.00 | 0.66 | Dalea elegans | Mimosa cordobensis |
| Sierra Chaco-Sierra Chaco | 0.66 | 0.34 | 0.00 | Mimosa cordobensis | Dalea elegans |
| Sierra Chaco-Sierra Chaco | 0.00 | 1.00 | 0.37 | Galactia glaucophylla | Indigofera kurtzii |
| Sierra Chaco-Sierra Chaco | 0.37 | 0.63 | 0.00 | Indigofera kurtzii | Galactia glaucophylla |
| Sierra Chaco-Sierra Chaco | 0.00 | 1.00 | 0.50 | Galactia glaucophylla | Mimosa cordobensis |
| Sierra Chaco-Sierra Chaco | 0.50 | 0.50 | 0.00 | Mimosa cordobensis | Galactia glaucophylla |
| Sierra Chaco-Sierra Chaco | 0.23 | 0.76 | 0.37 | Indigofera kurtzii | Mimosa cordobensis |
| Sierra Chaco-Sierra Chaco | 0.37 | 0.63 | 0.23 | Mimosa cordobensis | Indigofera kurtzii |
| Sierra Chaco-Upper Paraguay River Basin | 0.00 | 0.00 | 0.00 | Indigofera kurtzii | Arachis lignosa |
| Sierra Chaco-Upper Paraguay River Basin | 0.00 | 0.00 | 0.00 | Arachis lignosa | Indigofera kurtzii |
| Sierra Chaco-Upper Paraguay River Basin | 0.00 | 0.00 | 1.00 | Indigofera kurtzii | Bauhinia hagenbeckii |
| Sierra Chaco-Upper Paraguay River Basin | 0.00 | 0.00 | 1.00 | Bauhinia hagenbeckii | Indigofera kurtzii |
| Sierra Chaco-Upper Paraguay River Basin | 0.00 | 0.00 | 0.00 | Indigofera kurtzii | Neltuma rubiflora |
| Sierra Chaco-Upper Paraguay River Basin | 0.00 | 0.00 | 0.00 | Neltuma rubiflora | Indigofera kurtzii |
| Sierra Chaco-Upper Paraguay River Basin | 1.00 | 0.00 | 1.00 | Arachis correntina | Indigofera kurtzii |
| Sierra Chaco-Upper Paraguay River Basin | 1.00 | 0.00 | 1.00 | Galactia longifolia | Indigofera kurtzii |
| Sierra Chaco-Low Paraguay River Basin | 1.00 | 0.00 | 1.00 | Indigofera kurtzii | Arachis correntina |
| Sierra Chaco-Low Paraguay River Basin | 1.00 | 0.00 | 1.00 | Indigofera kurtzii | Galactia longifolia |
| Sierra Chaco-Low Paraguay River Basin | 0.00 | 0.00 | 0.00 | Indigofera kurtzii | Mimosa pseudopetiolaris |
