# Supplementary material for: Identifying areas and centers of endemism in the Gran Chaco with Fabaceae as a diversity indicator
Source: Sci Rep. 2025 Mar 20;15:9572. doi: 10.1038/s41598-025-90091-3 (PMC11926246; doi:10.1038/s41598-025-90091-3)
Supplement: Supplementary file 8 — Supplementary Material 8 [file 41598_2025_90091_MOESM8_ESM.docx]

**Selected specimens examined**

***Adesmia cordobensis***

**Argentina.** *Anderson 2092* (SI);*Barboza et al. 1172* (SI); *Burkart 1682, 7400, 10299, 20442,* ***20795*** (SI); *Cabrera et al. 29651* (SI); *Castellanos 96* (SI); *Dawson s.n.* (SI); *Deginani et al. 2123* (SI); *Giardelli 675* (SI); *Jörgensen 963* (SI); *Krapovickas 47553* (SI); *Lanfranchi 1251, 1479* (SI); *Nicora s.n., 1297, 1532, 2602, 2955, 9158* (SI); *Ragonese & Piccinini 9766* (SI); *Roig 1011* (SI); *Ruiz Leal 19870, 21175* (SI); *Ruiz Leal & Roig 20108* (SI); *Sayago 523b, 819, 889* (SI); *Schreiter s.n.* (SI); *Sleumer 2186* (SI); *Zuloaga et al. 14606* (SI).

***Adesmia macrostachya***

**Argentina.** *Ariza Espinar 2228* (CORD); *Burkart 5884* (SI); *Cabrera 28086* (SI); *Correa 8788* (BAB); *Giardelli 164* (SI); *Hunziker, A. T. 24373* (CORD); *Lanfranchi 808* (SI).

***Aeschynomene magna***

**Brazil.** *Leme & Souza 55* (CGMS). **Paraguay.** *Fiebrig 4059* (GH).

***Aeschynomene paraguayensis***

**Paraguay.** *Hassler 6694* (NY); *Pedersen 9301* (SI).

***Apurimacia dolichocarpa***

**Argentina.** *Cabrera et al. 29655* (SI); *Castellanos s.n.* (SI); *Hieronymus 386* (SI); *Hunziker 8838* (SI); *Zuloaga et al. 11318* (SI); *Roig Herb.RuizLeal 20083* (SI); *Sayago 967* (SI).

***Arachis batizocoi***

**Bolivia**. *Cárdenas 4741* (LIL); *Krapovickas 9484* (CTES); *Krapovickas et al. 30079, 30080, 30081, 30083* (CTES); *Saravia Toledo et al. 11442* (LIL). **Paraguay.** *Schinini 25658* (CTES); *Schinini et al. 25704* (CTES).

***Arachis correntina***

**Paraguay.** *Krapovickas 12593, 37459* (CTES); *Schinini 19616* (CTES). **Argentina.** *Anzoátegui 1210* (CTES); *Arbo 1088* (CTES); *Burkart 27595, 27695* (SI); *Carnevali 2271* (CTES); *Gregory et al. 9557* (CTES); *Krapovickas 7890, 13782* (CTES); *Krapovickas 30048* (CTES); *Krapovickas et al. 11905, 11919, 11941, 13800, 15680, 24580, 30050* (CTES); *Martínez Crovetto & Schinini 10798* (SI); *Sayago 2133, 2933* (SI); *Spegazzini 10071* (SI)*; Schinini 7453, 15669* (CTES); *Schulz 11791* (SI); *Stephens 16* (CTES); *Zardini 644* (CTES); *Zardini et al. 644* (SI).

***Arachis duranensis***

**Bolivia.** *Seijo et al. 2928, 4021, 4022, 4023, 4024* (CTES). **Argentina.** *Hunziker 1635* (SI); *Krapovickas 8010, 8026, 38904, 46353, 46561* (CTES); *Korovitz 5580* (SI); *Novara 11404* (CTES); *Schinini 21764, 21767* (CTES); *Seijo et al. 2737, 2741, 2772, 2822, 2823, 2825, 2826, 2857* (SI, CTES).

***Arachis lignosa***

**Brazil.** *Alves & Sartori 504* (CGMS). **Paraguay.** *Hassler 7476* (G); *Meyer 18729* (LIL); *Rojas 14074* (SI); *Vanni et al. 1291* (CTES).

***Arachis microsperma***

**Brazil.** *Valls et al. 8711* (CEN, CTES).

***Arquita mimosifolia***

**Bolivia.** *Fiebrig 2987* (MO). **Argentina.** *Biurrun 4193* (MO), *5348* (SI); *Cabrera 29720* (MO); *Cabrera et al. 27108* (BAB); *Cantero 5932* (BAB); *Fortunato 5932* (BAB); *Hunziker, A. 13102, 16378* (MO); *Jörgensen 1336* (BAB38396); *Molfino s.n.* (BAB55055); *Sayago 2601* (MO); *Spegazzini P. L. s.n.* (BAB29086), s.n. (BAB29293); *Ragonese & Piccinini 9816* (BAB).

***Astragalus bergii***

**Argentina.** *Burkart 4573* (SI); *Giardelli 685a, 966* (SI); *Hunziker 7561, 9723* (SI); *Kiesling & Ferrari 6422* (SI); *Sayago 1423* (SI).

***Astragalus distinens***

**Argentina.** *Burkart 4573* (SI), *5391* (MO); *Castiglioni & Ragnese 6690* (BAB75570) *Hunziker, A. 7529, 7561, 9723, 13278, 18263* (SI); *Giardelli 685a, 966* (SI); *Kiesling & Ferrari 6422* (SI); *Krapovickas 6498* (SI); *Manetti 1216* (SI); *Molina & Hilfer 3396* (BAB); *Piccinini & Petetin 3020* (BAB), 3187 (BAB); *Prado 113* (SI); *Sayago B-41, 1423, 2241, 2318* (SI).

***Bauhinia argentinensis***

**Bolivia.** *Fuentes & Navarro 2290* (CTES); *Pensiero & Marino 4391* (BAB). **Paraguay.** *Arenas 1101* (CTES), *1507* (BAB), *1817* (CTES, SI); *Degen & Mereles 3271* (CTES); *Luckow et al. 4496* (BAB); *Pavetti & Rojas 10414* (SI); *Rojas 2111, 4823* (SI); *Schinini & Bordas 1639* (CTES); *Schinini & Palacios 25671* (CTES); *Zardini & Acosta 42266* (BAB); *Zardini & Apestegui 58283* (BAB); *Zardini & Báez 52454* (BAB); *Zardini & Duarte 49276* (BAB); **Argentina.** *Arenas 2346* (BAB); *Devoto & Alberti 1658* (BAB); *Fortunato 346* (BAB); *Fortunato et al.* *4517* (BAB); *Maranta et al. 149* (SI); *Morello s.n.* (SI): *Saravia Toledo 10769* (BAB); *Scarpa 692* (SI); *Schinini 35324* (BAB).

***Bauhinia hagenbeckii***

**Brazil**. *Sartori & Alves 1084* (CGMS); *Seleme 281* (CGMS). **Paraguay.** *Degen & Zardini 656* (BAB); *Ferrucci et al. 1586* (BAB); *Hassler 6958, 7898* (SI); *Krapovickas 13920* (SI); *Ramella et al. 2955* (BAB); *Rodríguez 14083* (SI); *Rojas 3311* (SI); *Schinini 4396, 6187* (SI); *Vanni & Kurtz 3685* (BAB); *Zardini & Velázquez 19200* (BAB).

***Chaetocalyx chacoensis***

**Bolivia.** *Fuentes 2383* (CTES, USZ); *Joaquín s.n.* (CTES). **Paraguay.** *Bernardi 20273* (G); *Mereles 4971, 6672* (CTES); *Rojas 7398* (SI); *Schinini & Palacios 25722* (G); *Utzschneider 16* (CTES).

***Chamaecrista arachiphylla***

Paraguay. *Charpin & Ramella 21426* (BAB); *Fortunato et al. 8570* (BAB); *Krapovickas 45456* (BAB); *Zardini & Acosta 42226* (BAB).

***Chloroleucon chacoense***

**Brazil.** *Alves & Sartori 486a,b* (CGMS). **Bolivia.** *Fuentes & Navarro 2420, 2438* (CTES); *Fuentes & Wirandaco 2403* (CTES); *Meyer 18220* (SI); *Nee 54858* (MO). **Paraguay.** *Brunner 1235* (MO); *Fortunato et al. 4686* (BAB, CTES). **Argentina**. *González & Morello 5* (SI); *Meyer 18002* (SI).

***Clitoria cordobensis***

**Argentina.** *Estrada s.n.* (BAB); *Lahitte s.n.* (BAB); *Lamarque s.n.* (BAB91969).

***Dalea elegans***

**Bolivia.** *Chiapella 2927* (CORD). **Argentina.** *Anderson 1970* (CORD); *Barros 760, 763* (SI); *Burkart 6622, 10287, 10305* (SI); *Cantero 5464, 5513, 5554, 5616, 6083* (CORD); *Castellanos s.n.* (SI142492, 142498); *Giardelli 686* (SI); *Hawkes 3288* (SI); *Hunziker, A. T. 9717, 10262, 11760, 11764, 11774, 13820, 17979* (CORD, SI); *Isern 8142* (SI); *Kiesling 1657, 5156, 5804*(SI); *Krapovickas 7595* (SI); *Kurtz 15786* (CORD); *Lanfranchi 630* (SI); *Novara 3249* (SI); *Rojas*  *Schreiter 9741* (SI); *Sayago 497* (SI); *Sleumer 4008* (SI); *Stuckert 10637* (CORD); *Subils 941* (CORD); *Zavala-Gallo 255* (SI); *Zuloaga et al. 10164* (SI).

***Denisophyton stuckertii***

**Bolivia.** *Fuentes et al. 679* (MO); *Gentry & Foster 75401, 75403* (MO). **Paraguay.** *Arenas 3294* (MO). **Paraguay.** *Beck & Liberman 9443* (SI); *Brunner 1654* (MO); *Degen 2742, 2793, 3021, 3163* (MO); *Fortunato et al 371* (BAB)*, 8757* (SI); *Gragson 255* (MO); *Mereles 4841, 5460, 7667* (MO); *Rojas 7329* (SI); *Schinini 25716* (MO); *Spichiger 2265* (MO); *Zardini & Basualdo 3533* (MO); *Zardini & Guerrero 42424* (MO). **Argentina.** *Krapovickas 30385* (CTES, MO); *Renvoize 3538* (MO); *Seijo et al. 2729* (CTES, MO).

***Desmanthus tatuhyensis* var. *brevipes***

**Argentina.** *Alonso & Fosatti 832* (SI); *Burkart 5901, 5923* (SI); *Cordini 97* (SI); *Pensiero 7137* (MO), *8482* (SF); *Hunziker A. T. 9411* (CORD, SI); *Pedersen 6956* (SI); *Rodrigo 2475* (SI); *Sayago 1701* (SI); *Schinini 22370* (F); *Venturi 429* (NY), *5444* (SI).

***Desmodium burkartii***

**Argentina.** *Quarín 3513* (CTES); *Pedersen 3107* (CTES).

***Desmodium intermedium***

**Argentina**. *Jörgensen 2685 , 2721* (SI).

***Dolichopsis paraguariensis***

**Paraguay.** *Arenas 544* (BAB); *Hahn 2167* (BAB); *Zardini 38038, 46107* (BAB). **Argentina.** *Cabral 61, 1301* (BAB); *Cabral & Molina 779* (BAB); *Clos 3120, 3281* (BAB); *Cano 673* (BAB); *Cristóbal & Krapovickas 2165* (BAB; *Fortunato et al. 1685, 2022, 2272, 2406, 2968, 3458, 4089, 6476, 6655, 6955* (BAB); *Guaglianone & Múlgura 2218* (BAB; *López et al. 81* (BAB); *Maruñak et al. 400* (BAB); *Molina & Sánchez 2391* (BAB)*; Petetin & Molina 1241* (BAB); *Petetin et al. 1583* (BAB); *Piccinini & Hilfer 3203* (BAB); *Pire & Mroginsky 135* (BAB); *Schulz 7188, 7396, 15879, 17466* (BAB); *Quarín 4215* (BAB); *Solís Neffa 513* (BAB); *Vanni 719* (BAB).

***Erythrostemon argentinus***

**Bolivia.** *Arroyo et al. 3249* (SI); *Brooke 5566* (SI); *Cabrera & Gutiérrez 33647* (SI); *Fuentes 2390* (SI); *Krapovickas 31325* (SI); *Rojas 8354* (SI). Paraguay. **Argentina.** *Guaglianone et al. 2574* (SI).

***Erythrostemon coluteifolius***

**Bolivia.** *Vargas & Navarro 1922* (K). **Paraguay.** *Beck & Lieberman 9442* (SI); *Brandt 17* (SI); *Degen 3029* (MO); *Mereles 4931* (MO); *Rojas 7310* (SI); *Schinini & Palacios 25717* (SI). **Argentina.** *Burkart et al. 30483* (MO); *Fortunato et al. 7474* (BAB, MO); *Galetto 132* (MO); *Lillo 7237* (SI); *Lorentz 1004* (CORD); *Peirano s.n.* (SI); *Rodríguez 225* (SI); *Venturi 1117* (MO), *8477* (SI).

***Galactia glaucophylla***

**Argentina.** *Anderson 3081* (CORD); *Ariza Espinar 423, 1307, 1533* (CORD); *Burkart 1298, 7346* (SI); *Cantero 5374, 5391, 5443, 5478,5643, 5843, 6086, 6119, 6551, 6783, 6817 ; Castellanos s.n.* (SI); *Coccucci 418* (CORD); *Fortunato & Micheli 5082* (BAB); *Giardelli 7364* (SI); *Gillies s.n.* (SI); *Hunziker, A. T. 6243, 7816, 7874, 8028* (CORD), *8028* (SI); *Krapovickas 47254* (CTES); *Luti s.n.* (CORD); *Ragonese 6307* (BAB); *Pastore 50, 363, 2062, 6623* (SI); *Pérez Moreau & Petetin 4142, 4373* (BAB); *Piccinini & Hilfer 3920* (BAB); *Pozner & Belgrano 319* (BAB); *Rentzell s.n.* (SI); *Sayago 521b* (SI); *Stuckert 12108* (CORD).

***Galactia longifolia***

**Argentina.** *Cabral et al. 934* (BAB); *Fortunato et al. 669, 2151, 2797* (BAB); *Ragonese & Castiglioni 6977* (BAB); *Schulz 4100, 12766, 16421, 17745, 17810* (BAB), *Tressens & Keller 6950* (BAB).

***Indigofera guaranitica***

**Argentina.** *Fortunato et al. 2590* (BAB); *Maruñak 613* (MO); *Pedersen 2959* (MO); *Schulz 19078* (BAB).

***Indigofera kurtzii***

**Córdoba.** *Ariza Espinar 2441* (CORD); *Hieronymus 847* (CORD); *Nicora 2495, 2958* (SI); *Stuckert 8223* (LIL).

***Indigofera parodiana***

**Bolivia.** *DeMatteis 2311* (SI). **Paraguay.** *Balcazar 85* (BAB); *Luckow et al. 4488, 4494* (BAB); *Zardini & Duarte 42458, 49881* (BAB). **Argentina*.*** *Biurrun & Pagliari s.n.* (SI); *Cabrera et al. 27840* (BAB); *Correa et al. 4223* (BAB); *Fortunato et al. 426, 1583, 3385, 3407, 3808; 4399, 6828, 6845, 7181* (BAB); *Fortunato & Lamarque 5338* (BAB); *Krapovickas 30787* (SI); *Maldonado 545* (SI); *Nicora 2495* (SI); *Pérez Moreau & Petetin 4372* (BAB); *Pierotti s.n.* (SI); *Ragonese 3305* (BAB); *Renolfi 401* (BAB); *Saravia Toledo 1257* (SI); *Schinini 35337* (BAB); *Schinini & Pire 24185* (BAB); *Seijo & Krapovickas 1951* (BAB); *Venturi 1191, 1678* (SI).

***Lathyrus nigrivalvis***

**Argentina.** *Burkart 23684, 26980* (SI); *Boelcke et al. 13325* (BAB); *Flossdorf s.n.* (BAB24920); *Girola & Lahitte s.n.* (BAB43007); *Fortunato et al. 2059, 2542* (BAB); *Hunziker, A. 9616* (SI); *Jörgensen 2689* (SI); *Meyer 678* (SI); *Schulz 1189, 3137, 7403* (BAB), *17901* (CTES); *Venturi s.n.* (BAB3296).

***Libidibia paraguariensis***

**Bolivia.** *Fuentes Claros 498, 3373* (MO); *Gentry 51773, 75195, 75353* (MO); *Killeen 1293* (MO); *Lazarte 22* (MO); *Nee 39994, 40206, 44660, 51152, 51182, 51259, 53179, 54099, 56115, 60960* (MO, NY); *Peñaranda 54, 207* (MO); *Rico Arce et al. 1595* (MO); *Saldías 1411* (MO); *Seijo et al. 3166* (CTES); *Vargas Caballero 463* (MO). **Paraguay.** *Gragson 169* (MO); *Hahn 1387, 2157, 2181* (MO); *Little 40054* (MO); *Mereles 531, 3576, 3832, 4255, 4575, 4853, 4911, 5104, 5583, 5595, 5635, 5755, 5583, 5595, 5929, 6294, 7380, 8882* (FCQ); *Pérez de Molas 1058, 1215, 1341* (MO)*, 7752* (FCQ); *Schumeda 859* (FCQ); *Spichiger 2159, 2527, 2587* (FCQ); *Wright 14* (MO); *Zardini et al. 57895* (BAB, MO); *Zardini & Guerrero 36350, 37914, 38001, 39222, 40369, 57964* (BAB, MO); *Zardini & Tilería 35353, 39190* (BAB, MO); *Zardini & Velázquez 19907* (MO); *Zardini & Vera 41696* (BAB, MO). **Argentina.** *Fortunato et al. 199, 1305, 5191, 6035, 6197* (BAB); *Hunziker A. T. 17504, 24344* (CORD); *Krapovickas 23761* (CTES); *Morrone 2785, 3097, 4448* (SI); *Novara 3071, 3856* (MO); *Renvoize 3385* (MO); *Schreiter 5095* (SI); *Seigler 10109* (MO); *Venturi 5324, 5650* (MO); *Zuloaga et al. 6271* (MO, SI).

***Lophocarpinia aculeatifolia***

**Paraguay.** *Arenas 1729* (SI); *Fortunato et al. 8650, 8739* (BAB, SI); *Vanni et al. 2137* (SI), *2625* (CTES). **Argentina.** *Burkart 20216* (SI); *Fortunato et al. 1417, 1487, 4523, 6500, 6517, 6526* (BAB); *Ragonese & Castiglioni* s.n. (SI); *Scarpa 697* (SI).

***Mimosa castanoclada***

**Bolivia.** *Fuentes & Navarro 2277, 3638* (CTES); *Mereles & Degen 4507* (CTES); *Saravia Toledo 2862* (CTES); *Saravia Toledo et al. 11754* (CTES). **Paraguay.** *Fortunato et al. 3621* (BAB); *Hahn 1608* (BAB); *Mereles 2849* (CTES), *5484* (FCQ); *Mereles & Degen 4507* (CTES); *Mereles & Ramella 2849* (CTES); *Ramella et al. 2911* (BAB); *Schinini 14995* (CTES); *Schinini & Bordas 15114, 16416* (CTES); *Schinini & Palacios 25725* (BAB, CTES); *Zardini & Godoy 50150* (BAB); *Zardini and Rivas 58521* (BAB).

***Mimosa centurionis***

**Paraguay.** *Fiebrig 4001, 4581* (G).

***Mimosa chacoënsis***

**Bolivia.** *Fuentes 3384* MO); *Satavia Toledo & Nelson Joaquín 10312* (BAB); Pensiero & Marino 283 4379 (NY, SI). **Argentina.** Bordón s.n. (CTES 5719).

***Mimosa cordobensis***

Argentina. *Barboza et al. 4192* (CORD); *Hunziker, A. T. et al. 24372* (CORD).

***Mimosa craspedisetosa***

**Bolivia.** *Ramella & Mereles 2632* (BAB, MO).

***Mimosa detinens***

**Paraguay.** *Hahn 1856* (BAB); *Luckow et al.* 4491 (BAB); *Zardini & Duarte 49916* (BAB)**. Argentina.** *Álvarez 218* (CTES); *Brizuela 763* (CTES); *Duarte 461* (CTES); *Krapovickas 27167* (CTES); *Krapovickas & Cristóbal 46282* (CTES); *Salgado 447* (CTES); *Schinini 19510* (CTES); *Schulz 8292, 11275, 15508, 17457* (CTES); *Venturi 960* (CTES).

***Mimosa morongii***

**Paraguay.** *Fortunato et al. 9187* (BAB); *Morong 728* (NY); *Pedersen 9493* (MBM); *Pérez 230A* (AS).

***Mimosa pseudopetiolaris***

**Paraguay.** *Balansa 1465* (NY); *Hassler 5032* (NY); *Pedersen 9538* (SI); *Zardini 8728, 8688* (BAB); *Zardini & Florentín 9134* (BAB); *Zardini & Guerrero 37623* (BAB); *Zardini & Tillería 28096, 28908* (BAB); *Zardini & Velázquez 15930, 15949, 16812* (MO).

***Mimosa sensibilis***

**Brazil.** *Bueno et al. 65* (COR); *Candido Pereira & Pereira* 24 (COR); *Cabrera & Gutiérrez 33601* (CTES); *Conceição 1567* (NY); *Hatschbach et al. 29510, 49240* (NY), 73010 (MBM270107), *60762* (CTES), *60846* (BAB); *Pott 6466* (COR); *Pott A. et al. 1447* (COR), *4834* (CTES), *8434* (COR); *Salis et al. 974* (COR); *Silva et al. 13, 22* (COR). **Bolivia.** *Beck 27532* (LPB); *De Matteis et al.* *1045* (CTES); *Hatschbach 70067* (NY); *Hieronymus & Lorentz 618* (GOET); *Kempes & Mercado 3* (LIL); *Nee 35129, 38950* (LBP), *41629, 49366, 53077, 53112* (NY); *Krapovickas 36093* (NY); *Kuntze s.n.* (NY);*Saravia Toledo & Nelson Joaquín 10901* (BAB); *Saravia Toledo et al. 11583* (LPB); *Solomon & Nee 18019* (SI); *Seijo & Solís Neffa 3118* (CTES); *Steinbach 8120, 2555* (LIL); *Wood 8390* (LPB); *Wood & Goyder 16810* (LPB). **Paraguay.** *Bernardi 20422* (MO); *Hahn 1542* (MO); *Mereles 6635* (CTES); *Mereles et al. 9055* (CTES); *Pérez de Molas & Navarro 8902* (BAB); *Ramella et al. 2980* (BAB); *Rojas 13619* (LIL); *Schinini et al. 21159* (CTES); *Zardini & Rivas 5851, 58497* (BAB). **Argentina.** *Cabrera et al. 14562* (BAB); *Fortunato et al. 8385* (BAB); *Krapovickas & Cristónal 46331* (NY); *Legname et al. 10169* (CTES); *Medán & Tortosa s.n.* (BAA17335).

***Mimosa tobatiensis***

**Bolivia.** *Mamani & Saucedo 676* (K). **Paraguay.** *Fortunato et al. 9177* (BAB); *Hassler 12501* (G); *Sparre &Vervoorst 1504* (BAB).

***Mimosa troncosoae***

**Paraguay.** *Pérez de Molas & Navarro 8890* (BAB).

***Mimozyganthus carinatus***

**Bolivia.** *Billiet 6022* (MO); *Menacho 71* (MO); *Navarro 260* (MO); *Navarro Sánchez 2148* (MO); *Parada 4111* (MO); *Saravia Toledo 12045* (CTES); *Wood 22460* (MO). **Paraguay.** *Arenas s.n.,3299, 3801* (CTES); *Mereles 8296* (CTES); *Pérez de Molas 1335* (MO);*Verena 21* (CTES); *Zardini & Guerrero 45905* (BAB). **Argentina.** *Arenas 3013* (CTES); *Fortunato et al. 1443, 4521, 6409, 7353, 7475, 7559, 7567* (BAB); *Galetto 173* (CORD); *Gentry 51824, 75188* (MO); *Gragson 197* (BAB); *Marmel et al. 8677C* (CTES); *Múlgura et al. 692* (SI); *Sánchez 54* (CTES); *Scarpa 573* (SI); *Suárez 43* (CTES); *Torres 58* (CTES); *Vargas C.* (USZ); *Venturi 9968* (BAB).

***Neltuma affinis***

**Argentina.** *Arbo et al. 2184, 9337* (CTES); *Arenas BACP587, 1957* (CTES); *Ahumada et al. 2414, 3395* (CTES); *Cano 587, 2132* (BAB); *Carnevali 596, 4193* (CTES); *Cristóbal et al. 1370* (CTES); *Dematteis & Schinini 534* (CTES); *Dirección General de Tierras s.n.* (BAB); *Herbario Sección de Pasturas 2384* (CTES); *Herbst 1201* (CTES); *Jörgensen 361* (BAB); *Krapovickas et al. 11884, 20853, 27741* (CTES); *Morel 987, 1358* (CTES); *Núñez 894* (BAB); *Quarín et al. 354, 2181* (CTES); *Ragonese 2421, 2432, 2433, 2439* (BAB); *Ragonese & Castiglioni 8374* (BAB); *Rumiz 194* (CTES); *Salgado 490* (CTES); *Schinini 14032* (CTES); *Schinini et al. 12305, 13621, 31921* (BAB); *Schinini & Martínez Crovetto 12255* (CTES); *Schinini & Mroginsky 4491* (CTES); *Schinini et al. 31291* (CTES); *Schulz 1364, 1367, 1368, 3208, 13749* (CTES); *Schwarz 9196* (CTES); *Tressens et al. 2327* (CTES).

***Neltuma alba***

**Brazil.** *Pott 5295* (CTES). **Bolivia.** *Aronson 7792b* (CTES); *Beck & Barrientos 31516* (CTES); *Saldías et al. 1173* (CTES); *Seijo et al. 3094* (CTES); *Vargas C. 426, 851* (CTES). **Paraguay. *August 105* (CTES);** *Mereles 4156, 6472* (CTES); *Mereles & Degen 5399, 5842* (CTES); *Mereles et al. 7666* (CTES); *Palacios 1483, 1499, 1500, 1505* (CTES); *Schinini & Bordas 18188* (CTES); *Schulz 7834* (CTES); *Vogt 65* (CTES). **Argentina.** *Ahumada 1049* (CTES); *Arenas 3367* (CTES); *Bacigalupo et al. BAA10216.* (CTES); *Balegno 333* (CTES); *Castellanos s.n.* (CTES); *Cristóbal et al. 1370* (CTES); *Krapovickas et al. 27409, 45624* (CTES); *Legname & Cuezzo 5266C* (CTES); *Luna 343* (CTES); *Maidana 129* (CTES); *Quarín et al. 537* (CTES); *Schinini 12255, 13711* (CTES); *Schinini & Cristóbal 13664* (CTES); *Schinini et al. 19045, 29136* (CTES); *Suárez 289* (CTES).

***Neltuma campestris***

**Argentina.** *Burkart 12078, 13349* (SI); *Hunziker, A. T. 7084* (TEX); *Lorentz 2* (CORD); *Luti 4483, 4530* (SI); *Ragonese & Piccinini 9124* (Ctes); *Sayago 2139* (SI).

***Neltuma elata***

**Bolivia.** *Fuentes & Navarro 2240* (CTES). **Paraguay.** *Arenas 1091, 1797, 3792* (CTES); *Degen & Mereles 2882* (CTES); *Palacios 1481, 1488, 1502* (CTES); *Quintana et al. 668* (CTES); *Rojas 6978* (SI); *Schinini & Bordas 18193, 18134* (CTES); *Timmerman s.n.* (TEX). **Argentina.** *Devoto & Rial Alberti Herb. Forestal 1007* (SI); *Krapovickas & Cristónal 46280* (CTES); *Martínez Crovetto PM29* (CTES); *Ragonese & Castiglioni 7191* (CTES); *Ruiz Leal & Roig 17210* (SI); *Saravia Toledo 12681* (CTES); *Schinini 19580, 19575* (CTES); *Scopa 541* (CTES); *Suárez 80* (CTES).

***Neltuma fiebrigii***

**Paraguay.** *Arenas BACP1548* (CTES); *Caballero Marmori 1569* (CTES), *Fiebrig s.n.* (F1539477), *s.n.* (K000504844)*,* *1254* (K); *Mereles 4083* (CTES); *Pedersen 28534* (G); *Rojas s.n., 7227, 8229* (SI). **Argentina.** *Bacigalupo et al. BAA10211* (CTES); *Krapovickas 1207* (SI); *Maruñak et al. 441* (CTES); *Palacios s.n.* (*484* (SI), *Rojas 7227, 8229* (SI).

***Neltuma flexuosa***

**Argentina.** *Anderson 1441, 1442, 1448* (CORD); *Biurrun 2504* (CTES147938); *Burkart 7578, 15883* (SI); *Cabrera 27104* (CTES), *27086, 29740* (SI); *Cocucci 5954* (CORD); *Covas 957* (SI); *Del Vitto 3317* (CTES); *Hunziker, A. T. 11512, 11519, 12930* (CORD, SI)*, 12926* (CTES), *16288* (CORD); *Hunziker J. H. 2640, 2641* (SI); *Krapovickas & Cristóbal 24503* (CTES); *Martínez 1347* (CORD); *Roig 5658, 5666* (SI); *Ruiz Leal 13838, 18860, 23357* (SI); *Sayago 1480, 1481, 1564, 1575, 1967, 1969* (SI); *Solbrig 178* (SI); *Torres 33* (CTES); *Vila 5* (SI).

***Neltuma hassleri***

**Paraguay.** *Degen & Mereles* *3368, 3419* (CTES); *Florentín Peña & Mola 370* (CTES); *Fortunato et al. 3641* (BAB); *Hahn 1677* (CTES); *Mereles 517* (CTES); *Mereles & Degen 5387, 6173* (CTES); *Mereles & Greissler 3500* (CTES); *Palacios 1507, 1512 (CTES); Pavetti & Rojas 10807* (SI); *Pedersen 14633* (CTES); *Rojas 8450. 8477* (SI); *Schinini 18147* (BAB); *Schinini & Bordas 18147* (BAB); *Vanni et al. 1205* (CTES); *Zardini & Aquino 34283* (BAB); *Zardini & Duarte 49817* (BAB); *Zardini & Vera 46108* (BAB). **Argentina.** *Acuña Herb. Forestal 1884* (SI); *Insfrán 948* (CTES); *Martínez Crovetto PM-25* (CTES); *Meyer 1086* (SI); *Morel 4045* (LIL)*, 4307, 6560, 9091* (CTES); *Schinini 19599* (CTES).

***Neltuma kuntzei***

**Bolivia.** *Beck 7165* (CTES); *Saravia Toledo 12082* (CTES). **Paraguay.** *Arenas 1794* (CTES); *Egea & Peña 248* (CTES); *Fortunato et al. 8656* (BAB, CTES); *Krapovickas 44292* (CTES); *Mereles & Degen 5404* (CTES); *Schinini & Bordas 16570* (CTES); *Verena 30* (CTES); *Vogt &Mereles 211* (CTES). Argentina. *Alvarez 72* (CTES); *Bordón s.n.* (CTES); *Del Castillo 1156* (CTES); *Di Diacomo 537* (CTES); *Duarte 471* (CTES); *Krapovickas 17296,46851* (CTES); *KDTP 24* (CTES); *Medina 815* (CTES); *Pedersen 10730* (CTES); *Piccinini & Hilfer 4177* (CTES); *Salgado 449* (CTES); *Schinini 19571. 19991, 20016, 21659, 24240* (CTES); *Schinini & Pires 24917* (CTES); *Schulz 4074, 8937, 18309* (CTES); *Seijo & De Matteis 1149* (CTES); *Valla 213* (CTES); *Vanni et al. 4338* (CTES); *Vaca & Villa Carenzo 2806* (CTES).

***Neltuma nigra* var. *nigra***

**Bolivia.** *Saldías & Grupo de Dendrología 4052* (CTES); *Saravia Toledo et al. 11468, 11470* (CTES). **Paraguay.** *Arenas BACF623* (CTES); *de Egea et al. 127, 159* (CTES); *Degen & Mereles 2998, 3008, 3397, 3424, 3429* (CTES); *Florentín Peña & Molas 367* (CTES); *Hahn 693, 1852* (CTES); *Mereles 1410, 2984, 2990, 3011, 4166* (CTES); *Mereles & Degen 4690, 4848, 4888, 5288, 5817* (CTES); *Neiff 1920* (CTES); *Palacios 1486, 1487, 1510, 1511* (CTES); *Peña Chocarro et al. 1689* (CTES); *Quintana et al. 721* (CTES); *Schinini et al. 21075, 21226* (CTES); *Vanni et al. 1832, 2127* (CTES). **Argentina.** *Ahumada 5717* (CTES); *Ahumada et al. 1302, 1359* (CTES); *Arenas 3123* (CTES); *Bacigalupo et al. BAA9506, BAA10214* (CTES); *Biurrun & Pagliari 2485* (CTES); *Bordón 365* (CTES); *Carnevali 3336* (CTES); *Castellanos s.n.* (CTES); *Cristóbal et al. 1255, 1379* (CTES); *Fortunato et al. 4447* (BAB, CTES); *Huidobro 59* (CTES); *Krapovickas et al. 26457, 27633, 45623, 46891* (CTES); *Krapovickas & Cristóbal 20450* (CTES); *Keller 6182* (CTES); *Maturo & Prado 52, 238* (CTES); *Meyer 12811* (CTES); *Pedersen 13401* (CTES); *Placci & Arditi 155* (CTES); *Quarín & Tressens 1351* (CTES); *Salgado 410* (CTES); *Saravia Toledo 1483* (CTES); *Saravia et al. 10340* (CTES); *Schinini et al. 3517, 12274, 17283, 35017* (CTES); *Seijo & Dematteis 1148* (CTES); *Schulz 1354, 1357, 1358, 1359, 1360, 2000, 2008, 6732, 8367, 11308, 17458, 18160* (CTES); *Tressens et al. 2325* (CTES): *Vanni et al. 101* (CTES).

***Neltuma nigra* var. *longispina***

**Argentina.** *Fortunato et al. 3955* (BAB); *Pedersen 2808* (SI).

***Neltuma nigra* var. *ragonesei***

**Argentina.** *Burkart 5933* (SI).

***Neltuma nuda***

**Bolivia.** *Fuentes & Navarro 2409* (CTES, MO); *Gentry et al. 75174* (MO). **Paraguay.** *Arenas 1733* (CTES, SI); *Fortunato et al. 8654* (BAB, CTES); *Gragson 290* (MO); *Keller 6182* (CTES); *Mereles 3329* (FCQ); *Pedersen 13401* (CTES); *Quarín 3231* (CTES); *Schinini & Bordas 15222, 18151* (CTES); *Schinini et al. 11705* (CTES); *Tressens et al. 2326* (CTES); *Verena 67* (CTES), *Vogt & Mereles 257* (CTES).

***Neltuma pugionata***

**Argentina.** *Anderson 1399* (SI); *Bartlett 20541* (SI); *Biurrun 2479* (CTES); *Biurrun & Blanco 5413* (CTES); *Hunziker 12255, 12930* (CORD, SI); *Ocampo s.n.* (CORD); *Ragonese 6599, 6607* (BAB); *Ragonese & Caso s.n.* (BAB75478, CTES); *Sayago 1442, 1443* (SI), *1685* (CTES, SI); *Ulibarri 493* (SI).

***Neltuma rojasiana***

**Paraguay.** *Luckow et al. 4486* (BAB); *Pedersen 4171* (SI); *Rojas 8310, 8314* (SI); *Schinini & Bordas 16371, 18152* (CTES); *Vanni et al. 2026* (CTES); *Zardini & Guerrero 40405* (BAB).

***Neltuma rubriflora***

**Brazil.** *Alves et al. 65* (CGMS); *Hatschbach et al. 51648* (MO); *Laitart 81* (CGMS); *Martins et al. 210* (CGMS); *Matos-Alves et al. 65, 98, 345, 520* (CGMS); *Pott, A. 6872* (CGMS); *Pott, A. et al. 15408* (CGMS); *Pott, A. & Pott, V. J. 16849* (CGMS); *Praderi 346* (LIL); *Seleme et al. 268* (CGMS); *Terra 1* (CGMS). **Paraguay.** *Fiebrig 5348* (GH); *Palacios 1986* (MO); *Schulz 8841* (CTES).

***Neltuma sericantha***

**Paraguay.** *Angust 235* (CTES); *Arenas 483* (CTES); *Mereles 5455* (FCQ); *Palacios 1503* (CTES); *Rojas 7102* (SI). **Argentina.** *Arenas 483* (CTES); *Ariza Espinar 548* (CORD); *Baer s.n.* (BAB10007); *Balegno 17* (CTES); *Barboza 4425, 4598* (CORD); *Bartlett 19735* (SI), *19786* (CTES, SI); *Biurrun 490* (CORD); *Bordón 41* (CTES); *Brizuela 15, 712* (CTES); *Burkart 13973, 20361* (SI), *30486* (CTES,. SI); *Coccucci 362* (CORD); *Demaio 397* (CORD); *Dimitri s.n.* (BAB); *Fortunato 304* (BAB); *Fortunato & Micheli 5119* (BAB); *Fortunato et al. 1389, 6411* (BAB); *Giacomelli 1639* (BAB); *Guiñazú s.n.* (BAB); *Hieronymus 483* (CORD); *Hun ziker, A. T. 13586, 14097, 17840, 20565* (CORD); *Hunziker, J. A. 16660* (CTES); *Insfrán 855, 856* (CTES); *Jaime 15* (CTES); *Krapovickas 6072* (BAB), *46881 (*CTES*)*; *Krapovickas & Cristóbal 14504* (CTES); *Legname 84, 141* (CTES); *Luna 99* (CTES); *Martínez 1217, 1229, 1245* (CORD); *Martínez Crovetto PM105* (CTES); *Meyer 12784* (CTES); *Molina et al. 403* (BAB); *Múlgura 657* (SI); *Parodi 15022* (SI); *Piccinini & Leguizamón 1741* (BAB, CTES); *Piccinini & Petetin 3006* (BAB); *Ragonese s.n.* (BAB72882); *Ragonese & Caso 7411* (BAB, CTES); *Ragonese & Piccinini 6099, 6294* (BAB); *Rodríguez 184* (SI); *Roig 428* (BAB); *Romero 4* (BAB); *Ruiz Leal 8792* (SI); *Saravia Toledo 1481, 1801* (CTES); *Sayago 45* (CTES, SI)*, 56b* (SI); *Scarpa 539* (CTES); *Schinini 19595* (CTES); *Schreiter 299, 6715* (CTES); *Schulz 6544* (CTES); *Stramigioli 67, 73* (SI); *Suárez 195* (CTES); *Venturi 2682* (BAB); *Vulleumier 1029 8* (CORD).

***Piptadeniopsis lomentifera***

**Bolivia.** *Saravia Toledo 2683, 11755* (CTES). **Paraguay.** *Fortunato et al. 8795* (SI); *Hahn 1619* (CTES); *Luckow 4505* (BAB); *Rojas et al. 7033, 8457a* (SI); *Saravia Toledo 10049* (CTES); *Vanni et al. 2025, 2279* (CTES).

***Senegalia emilioana***

**Bolivia.** *Cabrera 33610, 33613* (SI); *Fuentes 2505* (CTES); *Fuentes & Navarro 2233, 2507* (CTES); *Saravia Toledo 2219, 10481* (CTES). **Paraguay. *Brandt 19* (SI);** *Fortunato et al. 8651. 8798* (BAB, CTES, SI); *Krapovickas 45515* (CTES); *Mereles 2142, 8772* (CTES); *Quintana et al. 560* (CTES); *Saravia Toledo 10046, 11776* (CTES); *Spichiger 2588* (CTES); *Vanni & Kurtz 3681* (CTES); *Vanni et al. 2102, 3681* (CTES); *Zardini 39254* (BAB, MO).

***Senna chacoensis***

**Argentina.** *Burkart 22053* (SI); *Cialdella et al. 579* (SI, MO); *Fortunato et al., 1552* (BAB, MO); *Mereles 5866* (MO); *Meyer 2037, 23386* (SI); *Rojas 7031* (MO); *Palacios & Branco 509* (SI); *Saravia Toledo 1264* (SI); *Sayago 386B* (SI); *Schulz 10148* (SI); *Schreiter s.n.* (SI); *Venturi 5831* (SI).

***Senna chloroclada***

**Bolivia.** *Beck 92* (SI); *Beck & Liberman 9389* (SI); *Chiraye 28* (MO); *De Matteis et al. 1976* (SI); *Gentry 75158* (MO); *Pflantz 947* (SI). **Paraguay.** *Basualdo 1261* (MO); *Degen 2755, 2795, 3101* (FCQ, MO); *Fortunato et al. 8637, 8649, 8652* (BAB, SI); *Mereles 662* (SI); *Rojas 7015, 7031, 7200, 8365, 8436* (SI); *Vanni et al. 1880, 2101* (CTES, SI). **Argentina.** *Arenas 2337* (BAB); *Cabral 746* (BAB); *Cordini 64* (SI); *Correa et al., 7668* (BAB); *Fortunato et al. 250, 269, 1398, 1402, 1507, 3411, 4481, 4482, 4576. 6395* (BAB); *Gragson 22* (MO); *Hahn 1450* (MO); *Krapovickas 1699* (SI); *Meyer 18180* (SI); *Molina et al. 244, 663* (BAB); *Molina & Sánchez 2499* (BAB); *Palacios 5722* (SI); *Piccinini & Hilfer 4109* (BAB); *Ragonese & Castiglioni 7864* (BAB); *Schulz 17440* (BAB); *Schinini 2331* (BAB); *Seijo et al. 2716* (BAB); *Suárez 6* (SI); *Vanni et al. 4330* (BAB).

***Senna subulata***

**Argentina.** *Brooks MS134* (mo); *Cantero 7505* (BAB); *Cocucci et al. 2605* (MO); *Correa Luna 9106* (BAB); *Clos 7299* (BAB); *D’Arcy 511* (SI); *Deginani 911* (MO); *Deletang & Guiñazú s.n.* (BAB); *Gez 1922* (SI); *Hieronymus s.n.* (SI); *Jorgensen Hansen 1335* (BAB); *Monetti 1936* (SI); *Múlgura et al. 1484* (MO); *Pastore s.n.* (SI); *Pozner & Belgrano 427* (BAB); *Rossow &Canale 40* (BAB); *Sayago 627* (MO), *1953* (SI); *Schreiter s.n.* (SI); *Spegazzini s.n.* (BAB 28945, 28662); *Venturi 2871* (BAB); *Vignatii 20* (SI).

***Stenodrepanum bergii***

**Argentina.** *Ariza 914* (CORD); *Fortunato et al. 9144, 9629* (BAB); *Molina et al. 1861* (BAB); *Piccinini & Leguizamón 1810, 1970* (BAB); *Soriano 787* (BAB);

***Strombocarpa abbreviata***

**Argentina.** *Brunch & Carette 100, 211* (SI); *Biurrun 4359* (CORD)*, 5011* (SI); *Devoto & Rial Alberti s.n.* (SI); *Hunziker et al. 21020, 22299, 25220, 25224* (CORD); *Lorentz 339* (SI); *Schreiter s.n.* (SI); *Soriano 793* (SI); *Tinto Herb. Dir. Forestal 2018* (SI).

***Stylosanthes recta***

**Paraguay.** *Charpin & RFamella AC21545* (G); *Krapovickas 44219* (CTES); *Pérez et al.2531, 2995* (CTES); *Vanni et al. 2173, 2448, 2495* (CTES).

***Tephrosia hassleri***

**Paraguay.** *Zardini & Duarte 49673* (BAB). **Argentina.** *Beccaceci & Vanni 4247* (CTES); *Carnevali 1054* (CTES).

***Tephrosia chaquenha***

**Paraguay.** *Hassler 2591* (G)*, 12410* (BM, G, SI); *Queiroz 1439* (UEC); *Schinini & Vanni 19043* (IAC).

***Vachellia astringens***

**Argentina.** *Beck et al. 11500* (SI); *Burkart 7374, 7676, 13368* (SI); *Cano 4410* (BAB); *Carnevali 3901* (CTES); *Castellanos 5612* (SI); *Dinelli 688* (BAB26847); *Hunziker 8944* (SI); *Kiesling et al. 9513* (SI); *Krapovickas 27743* (CTES); *Lamarque s.n.* (BAB91932); *Lanfranchi s.n.* (BAB); *Martínez Crovetto 6118* (BAB); *Pensiero 6166* (SI); *Ragonese 2401, 2378* (SI); *Ragonese & Castiglioni 8620* (BAB); *Ragonese & Piccinini 6564* (BAB71209); *Ruiz Leal 11651, 12094* (SI); *Salgado 35* (CTES); *Sayago 748, 1295, 1675, 1709, 2150,* (SI); *Schinini et al. 18731* (CTES); *Schreiter s.n., s.n.-LIL68667* (SI), *Tortorelli 16949* (SI); *Venturi 2466* (BAB40874)

***Vachellia caven* var. *microcarpa***

**Paraguay.** *Arenas CEFAPRIN200* (SI); *Mereles 1625* (SI); *Rojas 7056, 7697* (SI), *Zardini & Velázquez 16627, 18087, 25121, 28620* (BAB). **Argentina.** *Guaglianone & Múlgura s.n.* (SI); *Krapovickas 1283* (SI); *Ragonese & Cozzo s.n.* (SI).

***Vachellia curvifructa***

**Paraguay.** *Caballero Marmori 1510* (CTES); *Degen & Mereles 3378, 3406* (CTES); *Fortunato et al. 3767* (BAB); *Mereles & Degen 5814* (CTES); *Mereles et al. 8310* (CTES); *Schinini & Palacios 25873* (CTES); *Vanni et al. 1944* (CTES); *Zardini & Vera 46105* (BAB). **Argentina.** *Bordón s.n., 526* (CTES); *Fortunato et al. 4308* (BAB); *Krapovickas 934* (SI); *Morel 8930* (CTES); *Salgado 320, 505* (CTES).

***Vicia epetiolaris***

**Paraguay.** *Burkart 18359* (SI); *Pavetti & Rojas 9210* (SI). **Argentina.** *Ahumada 1175* (SI); *Bissio 289* (BAB); *Boelcke et al. 13298* (BAB); *Burkart 25982, 26604* (SI); *Pire 691, 1526* (SI); *Fortunato et al. 532, 717* (BAB)*; Herb. Secc. Pasturas 501* (BAB); *Krapovickas 13067, 19625* (SI); *Kurtz et al. 1* (BAB); *Piccinini & Leguizamón 2453* (BAB); *Schinini 11837, 32692* (SI); *Schulz 15674* (CTES); *Spegazzini, R. A. 31 (*BAB64520*); Vanni 12* (BAB); *Vegetti 221* (SI).

***Vicia graminea***

**Argentina.** *Ariza 1943* (MO); *Cult 938* (BAB); *Fortunato 195* (BAB); *Jörgensen 1121. 3204* (MO); *Pedersen 773* (MO); *Piccinni & Leguizamón 2676* (BAB); *Schulz 10229* (SI); *Valencia s.n.* (MO).
